# Anticancer Activity and Safety Profile of Novel 1-(4-Fluorophenoxyacetyl)-4-substituted Thio/Semicarbazide Derivatives

**DOI:** 10.3390/molecules30071576

**Published:** 2025-03-31

**Authors:** Paweł Kozyra, Ewelina Humeniuk, Zbigniew Karczmarzyk, Adrian Borzęcki, Grzegorz Adamczuk, Agnieszka Korga-Plewko, Waldemar Wysocki, Monika Pitucha

**Affiliations:** 1 Independent Radiopharmacy Unit, Faculty of Pharmacy, Medical University of Lublin, 20-093 Lublin, Polandadrian.borzecki@o2.pl (A.B.); 2 Independent Medical Biology Unit, Faculty of Pharmacy, Medical University of Lublin, 20-093 Lublin, Poland; ewelinahumeniuk@umlub.pl (E.H.); grzegorz.adamczuk@umlub.pl (G.A.); agnieszka.korga-plewko@umlub.pl (A.K.-P.); 3 Institute of Chemistry, University of Siedlce, 3 Maja 54, 08-110 Siedlce, Poland; zbigniew.karczmarzyk@uws.edu.pl (Z.K.); waldemar.wysocki@uws.edu.pl (W.W.)

**Keywords:** thiosemicarbazide, semicarbazide, cancer, X-ray, pharmacokinetics, prooxidation

## Abstract

Compounds with thiosemicarbazide and semicarbazide scaffolds are among the most promising structures in medicinal chemistry due to the possibility of forming multiple hydrogen bonds. Therefore, six new derivatives of 4-fluorophenoxyacetylthiosemicarbazide and 4-fluorophenoxyacetylthiosemicarbazide were designed to compare their physicochemical properties, biological activity, and in silico pharmacokinetic parameters. All compounds were characterized by ^1^H, ^13^C NMR, ^19^F, IR spectra. For selected derivatives (**AB2** and **AB5**), X-ray studies were performed to confirm their synthetic route and identify the tautomeric forms and intra- and intermolecular interactions occurring in the crystalline state. In the in silico pharmacokinetic study, a clear difference in lipophilicity was observed between thiosemicarbazide and semicarbazide derivatives. In vitro biological studies have shown the promising activity of thiosemicarbazides against prostate cancer cell line LNCaP, with a higher safety profile than semicarbazides. The most active compound **AB2** showed IC_50_ = 108.14 μM against LNCaP. Based on biological studies, topoisomerase IIα was proposed as a potential molecular target, which was confirmed by molecular docking studies.

## 1. Introduction

**Figure 1:**
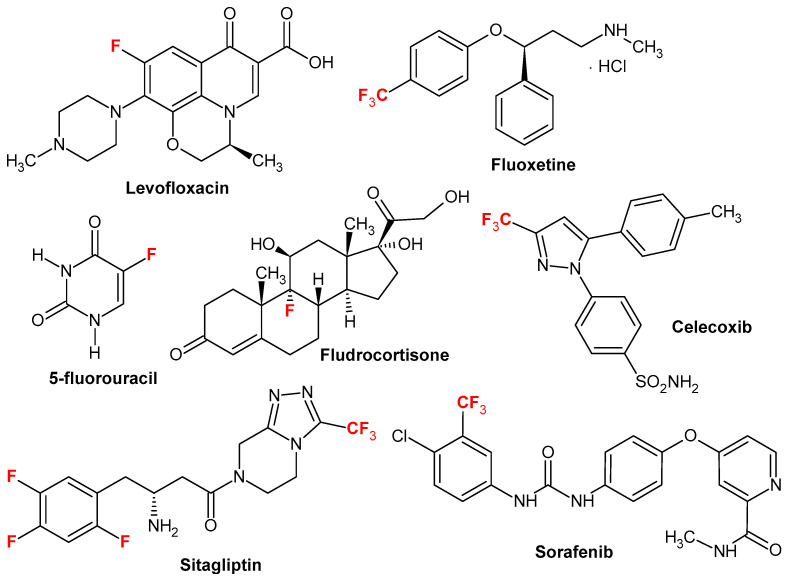
Selected FDA-approved drugs bearing fluorine atom.

Thiosemicarbazide and semicarbazide systems are promising structural fragments in medicinal chemistry. They owe their unique properties to the ability to form hydrogen bonds, which are the most important interactions of a potential drug with a biological target [1,2]. In fact, they are an important element for various drugs and bioactive compounds endowed with a wide range of therapeutic and pharmacological properties. Semicarbazide derivatives exhibit anticancer [3,4,5,6,7,8], antimicrobial [9,10], antioxidant [11,12,13], anticonvulsant [14,15,16,17,18], antiviral [19], antituberculosis [9,20], antimalarial [21], analgesic [22], and anti-inflammatory [22,23] activity. In turn, thiosemicarbazide derivatives exhibit anticancer [24,25,26], antimicrobial [9,27,28,29], antiviral [30,31], antioxidant [32], anticonvulsant [15,33,34], antitubercular [9,20,29,35], analgesic [28], anti-inflammatory [28] and antifungal [29] activity. Structurally, thio/semicarbazide derivatives differ only in the presence of sulfur or oxygen. This is a phenomenon called isosteric in medicinal chemistry, and the exchanged groups are isosteric groups. Replacing sulfur atom with an oxygen atom results in a change in physicochemical properties, molecular size, polarity, and electron distribution. This is crucial in creating a hydrogen bond network that stabilizes ligand-receptor interactions and recognizes bioactive sites. All these elements undoubtedly affect both the biological activity of compounds and their toxicity. Parks et al. reported that semicarbazide is about 1/20 as toxic as thiosemicarbazide [36]. Szopa et al. mentioned that replacing sulfur in thiosemicarbazide derivatives with an oxygen atom increases antitumor activity but also toxicity in zebrafish embryos [8]. On the other hand, 1-(4-nitrobenzoyl)-4-ethylsemicarbazide and 1-[(4-nitrophenyl)acetyl]-4-hexylsemicarbazide do not cause morphological abnormalities, including cardiotoxicity [8]. Moreover, the presence of a fluorine atom in the molecular structure of biologically active compounds may have a significant effect on their physicochemical properties. The introduction of a fluorine atom to the parent compound causes significant changes in the properties due to differences in the energy levels of HOMO and LUMO [37]. Morgenthaler et al. reported that the introduction of fluorine atom to drugs containing amino groups increases their bioavailability by regulating the pKa value [38]. It is worth mentioning that fluorine can be used as bioisostere of hydroxyl groups (-OH), because it acts as a weak hydrogen bond acceptor [39]. Moreover, the introduction of fluorine substituent, due to the high stability of C-F bond and electronegativity of fluorine [37,40,41], provides an increase in the metabolic stability of the drug [39,42,43]. Fluoridation also increases bioavailability by increasing the lipophilicity of the compound and thus increases the potency of the drug [37,44]. Fluorine atoms add inter- and intramolecular spaces that provide appropriate strong and selective interactions with enzymes and receptors [45,46]. Currently, 20% of pharmaceutical products contain fluorine compounds [37]. Figure 1 shows selected FDA-approved drugs that have fluorine atom in their structure.

This indicates a further need for optimization of thio/semicarbazide derivatives structure in order to optimize biological activity and toxicity profile. For this purpose, new thiosemicarbazide and semicarbazide derivatives containing 4-fluorophenoxyl fragment were designed. Their anticancer potential and cytotoxicity of obtained derivatives toward normal BJ fibroblast cells were investigated. Analysis of gene expression related to antioxidant defense, cell cycle regulation, DNA damage detection and repair, and apoptosis was performed. Molecular docking was performed to determine potential molecular target.

## 2. Results and Discussion

### 2.1. Chemistry

**Scheme 1:**
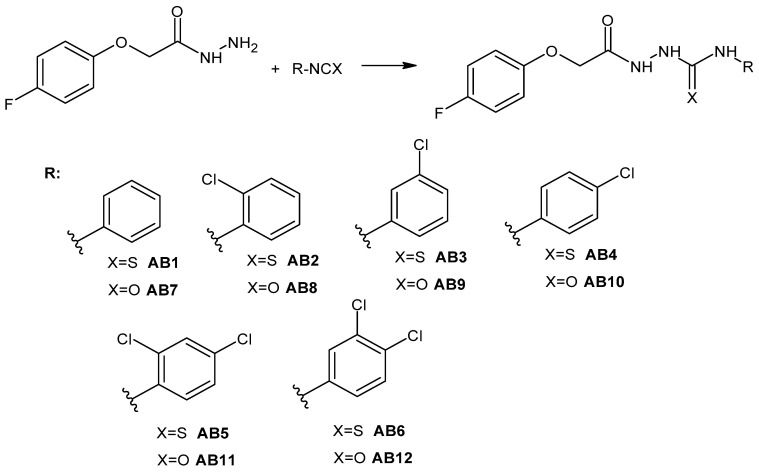
Synthesis pathway of 4-fluorophenoxy(thio)semicarbazide derivatives **AB1**–**AB12**.

The phenoxy group is a privileged scaffold in medicinal chemistry [47]. In our studies and previous works, we have shown promising anticancer activity of thiosemicarbazide derivatives with 2,4-dichlorophenoxy [25,26], phenoxy [24] and 4-fluorophenoxy [48] groups. Considering the valuable role of fluorine as well as reports on the activity and toxicological profile of thiosemicarbazide and semicarbazide, we designed new 1,4-disubstituted thiosemicarbazide (**AB1**–**AB6**) and semicarbazide (**AB7**–**AB12**) derivatives. The title compounds were obtained by the reaction of 4-fluorophenoxyacetic acid hydrazide with the following isothiocyanates: phenyl (**AB1**), 2-chlorophenyl (**AB2**), 3-chlorophenyl (**AB3**), 4-chlorophenyl (**AB4**), 2,4-dichlorophenyl (**AB5**), 3,4-dichlorophenyl (**AB6**) and with the following isocyanates: phenyl (**AB7**), 2-chlorophenyl (**AB8**), 3-chlorophenyl (**AB9**), 4-chlorophenyl (**AB10**), 2,4-dichlorophenyl (**AB11**), 3,4-dichlorophenyl (**AB12**). The reactions were carried out at the boiling point of methyl alcohol. The yield for thiosemicarbazide derivatives was in the range of 62–80%, while for semicarbazide derivatives it was 26–58%. Scheme 1 shows the synthetic path of the mentioned phenoxyacetyl(thio)semicarbazide derivatives.

The structures of the obtained compounds were confirmed by NMR and MS spectra. In the ^1^H NMR spectra, characteristic signals were observed for NH groups in the range of 9.56–10.36 ppm (for thiosemicarbazide derivatives) and 8.27–10.00 ppm, respectively, for semicarbazide derivatives. The presence of the carbonyl group (C=O) is confirmed by peaks in the range of about 181–182 ppm and the thion group (C=S) in the range of 167–168 ppm for thiosemicarbazides. For semicarbazide derivatives, a signal from the C=O group is visible in the range of 172 ppm. The presence of the above functional groups was also confirmed by ^19^F and infrared spectra. The purity of the obtained compounds was confirmed by MS spectrometry.

### 2.2. X-Ray Structure Determination

**Figure 2:**
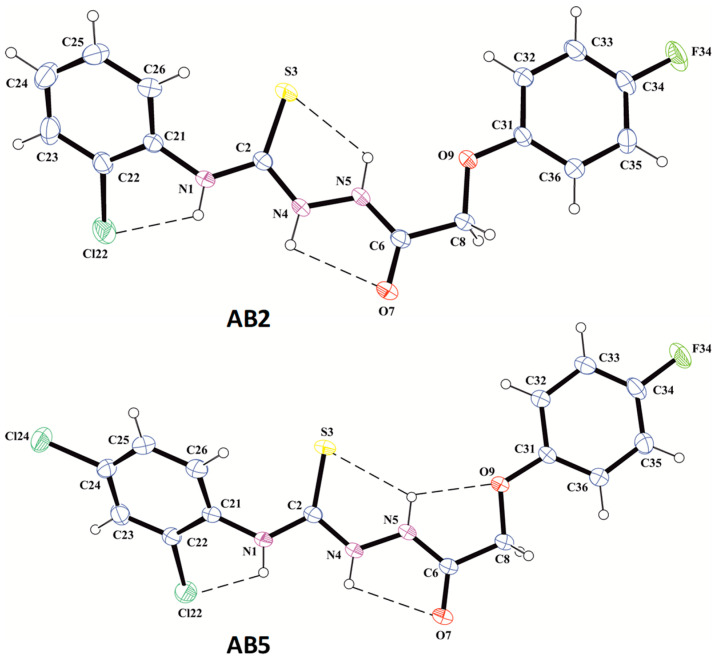
The molecular structures of compounds **AB2** and **AB5** in conformation observed in the crystal with atom labeling and displacement ellipsoids drawing at the 30% probability level. The dashed lines show the intramolecular hydrogen bonds.

X-ray analysis was carried out for **AB2** and **AB5** in order to confirm the synthetic route, the assumed their molecular structures, including the observed tautomeric form in the crystalline state, and to examine the crystal structure for the identification of intra- and intermolecular interactions. The molecular structures of **AB2** and **AB5** in the conformation observed in the crystal is shown in Figure 2.

X-ray analysis revealed that the compounds **AB2** and **AB5** exist in N1-amino/S3-thione/N4-amino/N5-amino/O9-keto tautomeric form with the position of the hydrogen atoms on the differential electron density maps in the immediate vicinity of the N atoms and bond lengths C2–S3 of 1.666(3) and C6–O7 of 1.237(3) Å in **AB2** and 1.668(3) and 1.236(3) Å in **AB5**, respectively. These bond lengths are typical for the thione (1.671(24) Å) and carbonyl (1.234(12) Å in amides) groups [49].

**Figure 3:**
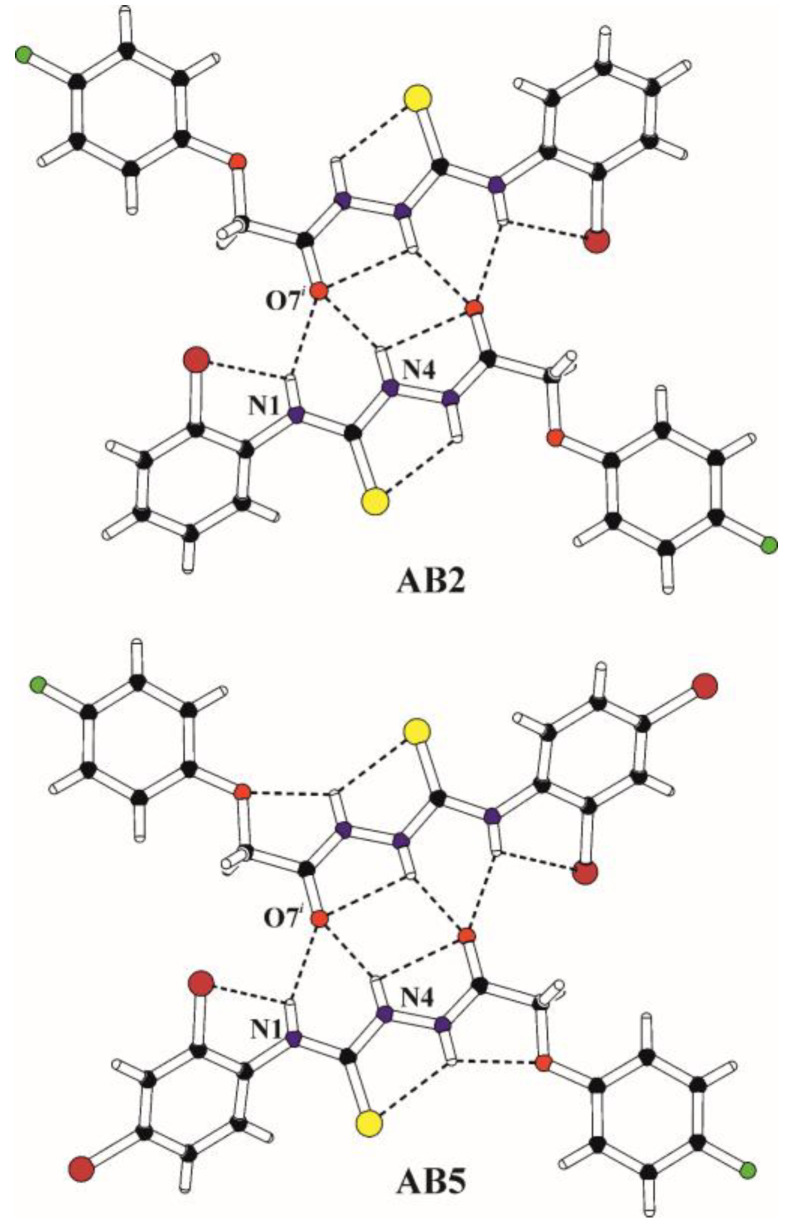
The structural motif (molecular dimer) formed by inversion-related molecules in the crystals of **AB2** (*i* = −*x*, 1−*y*, −*z*) and **AB5** (*i* = −*x*, 1−*y*, −*z*). The intra- and intermolecular hydrogen bonds are marked with dashed lines.

In both molecules, the acethylthiosemicarbazide chain connecting terminal phenyl substituents adopts almost planar conformation, and both benzene rings are arranged coplanar with respect to the plane of their chain linker. The appropriate torsion angles are presented in Table S1 (Supplementary Material). The conformations of molecules are stabilized by the intramolecular hydrogen bonds N1–H1…Cl22, N4–H4…O7 and N5–H5…S3 in **AB2** and N1–H1…Cl22, N4–H4…O7, N5–H5…S3 and N5–H5…O9 in **AB5** (Figure 3, Table S2 Supplementary Material) creating systems of five-membered rings designated as S(5) in graph set notation [50].

The molecular packing in the crystals of **AB2** and **AB5** is influenced by the bifurcated intermolecular N1–H1…O7 and N4–H4…O7 and hydrogen bonds with the amino N–H groups as proton donors and the carbonyl O atom as the protons acceptor strictly connected with the tautomeric form observed in the crystalline state (Figure 3, Table S2 Supplementary Material). Inversion-related molecules form molecular dimers via these hydrogen bonds in which the S(4) ring and two S(6) rings are conjugated to rings formed by intramolecular hydrogen bonds. The molecular dimers observed in the crystal structures of **AB2** and **AB5** are shown in Figure 3.

**Figure 4:**
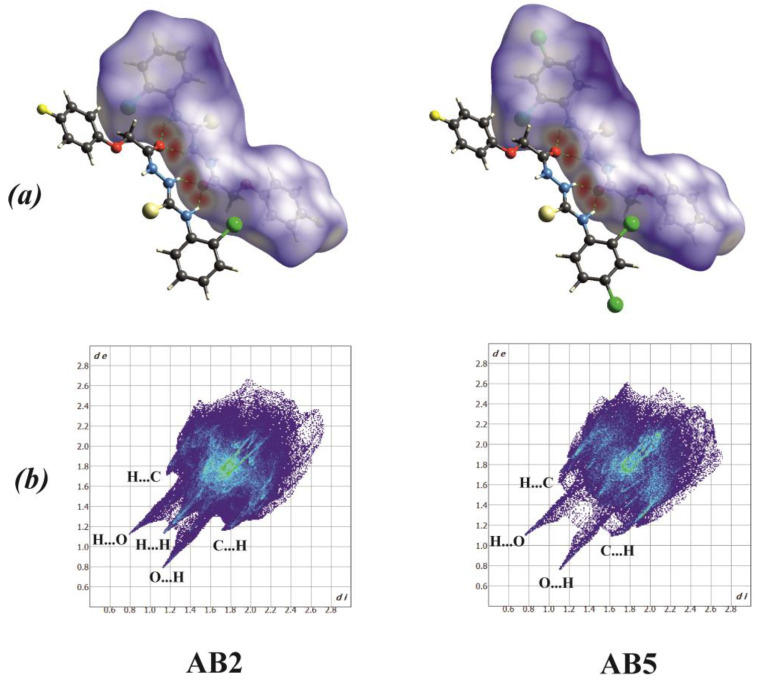
(**a**) Hirshfeld surfaces plotted over d_norm_; the hydrogen bonds are indicated by the red spots, (**b**) fingerprint plots of the nearest internal distance *d_i_* versus the nearest external distance d_e_.

A detailed characterization of intermolecular interactions was performed in crystal structures of **AB2** and **AB5** by the Hirshfeld method using CrystalExplorer Version 17 software [51]. This method included analysis of Hirshfeld surface and two-dimensional fingerprint plots presented in Figure 4a,b, where the distances shorter than the sum of the van der Waals radii of the nearest atoms are shown in red while the blue color indicates the low-intensity contacts.

The only similarity for **AB2** and **AB5** fingerprints is this presence of sharp H…O/O…H contacts with values of 8.9% and 8.3%, respectively. Fingerprint **AB2** has more distinct H…C/C…H (16.00%) contacts, as compared to **AB5**, where they are quite blurred and rounded and have a slightly higher value of 16.70%. Moreover, for the **AB2** fingerprint there is a clear sharp H…H contact (30.50%), which is poorly visible for **AB5** (22.90%).

**Figure 5:**
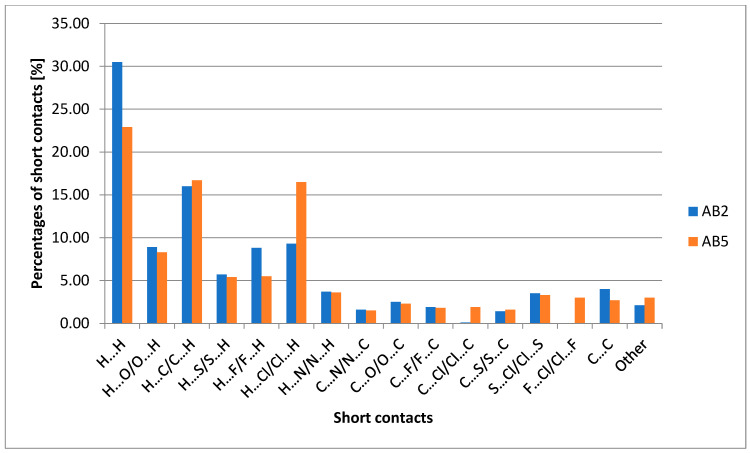
Chart comparing the percentage of short contacts for **AB2** and **AB5**.

It is worth mentioning that H…H interactions contributed the most for both structures but are associated with rather repulsive interactions. A graph comparing the percentage of short contacts for **AB2** and **AB5** is presented in Figure 5.

### 2.3. ADMET Analysis

**Figure 6:**
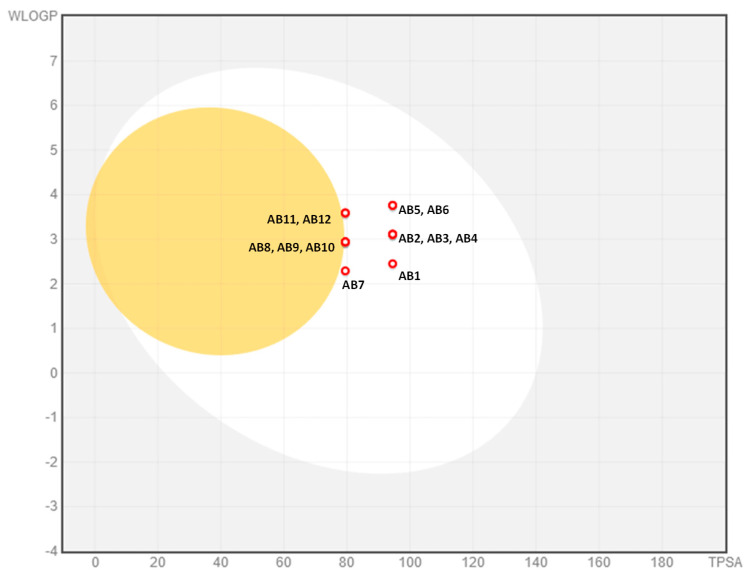
Dependence of logp (WLOGP) on the topological polar surface (TPSA). BOILED − Egg diagram for **AB1**–**AB12**.

High therapeutic effectiveness is ensured by the properties of ADMET. Therefore, predictive characterization of Absorption, Distribution, Metabolism, Elimination, and toxicity (ADMET) was performed. Guan et al. reported that both high effectiveness in relation to the therapeutic target and appropriate properties of ADMET characterize a good drug candidate [52]. The BOILED-Egg diagram indicated that both thiosemicarbazides (**AB1**–**AB6**) and semicarbazides (**AB7**–**AB12**) are passively absorbed in the gastrointestinal tract (Figure 6). This property makes them potentially effective drugs. None of the designed compounds cross the blood–brain barrier. The compounds obtained by us are not substrates for P-glycoprotein (P-gp). It should be mentioned that not all anticancer drugs are substrates for Pgp [53].

The Figure 6 shows the dependence of logp (WLOGP) on topological polar surface area (TPSA). The grey zone indicates compounds practically not absorbed by the gastrointestinal tract, the white sphere indicates compounds passively absorbed by the gastrointestinal tract, and the yellow sphere indicates compounds penetrating the blood–brain barrier.

**Figure 7:**
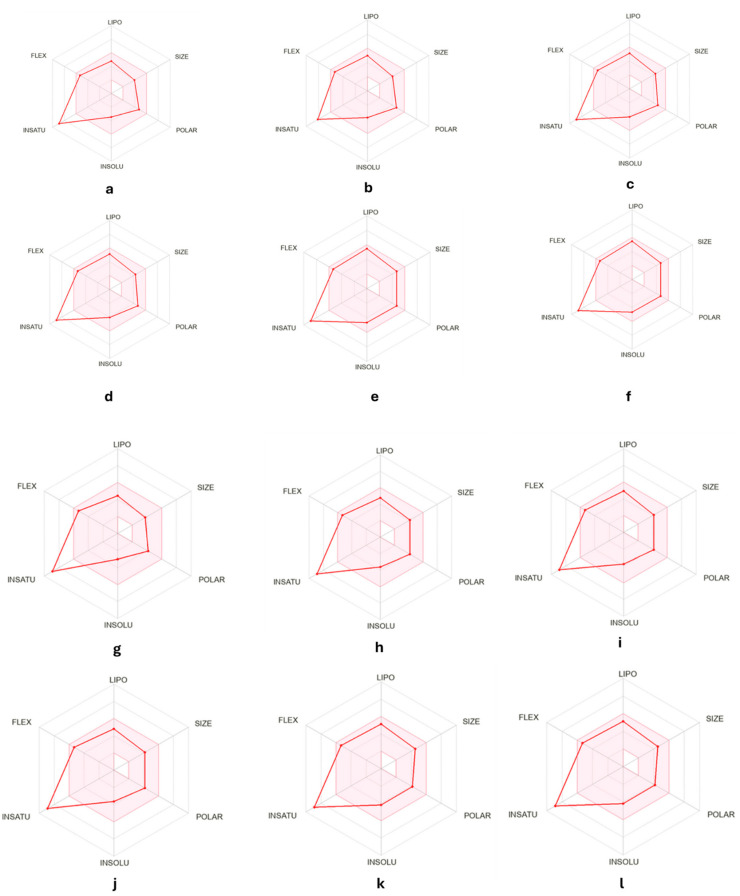
Bioavailability radar for (**a**). **AB1**, (**b**). **AB2**, (**c**). **AB3**, (**d**). AB4, (**e**). **AB5**, 208 (**f**). **AB6**, (**g**). **AB7**, (**h**). **AB8**, (**i**). **AB9**, (**j**). **AB10**, (**k**). **AB11**, (**l**). **AB12**. (lipophility (LIPO) are within the range 0.7 < XlogP3 < +5.0; molecular weight (SIZE) is 150 g mol 1 < MW < 500 g mol 1; polarity (POLAR) is 20 Å^2^ < Topological Polar Surface (TPSA) < 130 Å^2^; insolubility (INSOLU) is 0 < logS < 6; insaturation (INSATU) are 0.25 < Fraction Csp^3^ < 1; and Flexibility (FLEX) are 0 < Num. rotatable bonds < 9).

A bioavailability radar for the mentioned compounds was presented in Figure 7. As a result of an insufficient ratio of carbon atoms in sp^3^ hybridization in relation to the total number of carbon atoms in the molecule, all compounds are characterized by an exceeded saturation parameter. However, the remaining physicochemical parameters are adequate for oral bioavailability. All of the compounds were found to have a good bioavailability score (0.55) [54]. Druglikeness compliance analysis showed that the designed compounds were consistent with the Lipiński [55], Ghose [56], Egan [57], Veber [58] and Muegge rule [59]. Additionally, we present the putative prediction drug–drug interactions via cytochrome P450 (CYP) inhibition (Table 1). There is a clear difference in potential interactions with CYP1A2, where all semicarbazides do not have inhibitory properties, and for thiosemicarbazides, most of them have inhibitory properties, except for **AB1** and **AB2**. Similarly, this is the case for CYP2C9, where all thiosemicarbazides have inhibitory properties, and in the case of semicarbazides, only **AB8**, **AB11** and **AB12**. In the case of CYP2C19, only **AB7** does not have inhibitory properties. **AB6** has inhibitory properties for CYP2D6, and **AB4**, **AB5**, and **AB6** have inhibitory properties for CYP3A4 among the series of designed derivatives.

In order to confirm the safety profile of the **AB1**–**AB12**, prediction of toxicity was performed using the in silico method. Pro Tox-II classified **AB1**–**AB6** and **AB12** to toxicity class 4 (predicted lethal dose [LD_50_] = 500 mg/kg for **AB1**–**AB6** and [LD_50_] = 850 mg/kg for **AB12**) and **AB7**–**AB11** to class 5 (predicted [LD_50_] = 2500 mg/kg). This indicates that the designed semicarbazides have a substantially lower toxicity, as compared to thiosemicarbazides. Moreover, all compounds showed no immunotoxic activity.

### 2.4. Biological Activity

#### 2.4.1. Cytotoxicity Assessment

The biological activity of thiosemicarbazide derivatives (**AB1**–**AB6**) and semicarbazide derivatives (**AB7**–**AB12**) was evaluated in a panel of human cell lines, including non-cancerous BJ fibroblasts as normal cells and cancer cell lines representing different types of malignancies. These cancer cell lines comprised prostate cancer (PC-3, DU-145, LNCaP), melanoma (SK-MEL28, A375, G-361), breast cancer (MCF-7, MDA-MB-231), and lung cancer (NCI-H1563). The MTT assay was carried out across a wide range of concentrations for 48 h. Thiosemicarbazide derivatives were tested at concentrations ranging from 500 to 100 μM, whereas semicarbazide derivatives were evaluated at a more limited range of 100 μM to 1 μM due to their low solubility. The semicarbazides assessed at the maximum concentration of 100 µM did not achieve IC_50_ values in any of the tested cancer cell lines and showed a low level of cytotoxicity toward cancer cells, which led to their exclusion from further studies.

To determine the selectivity of the thiosemicarbazide derivatives (**AB1**–**AB6**), toxicity to normal cells (BJ fibroblasts) was carried out. Among all tested derivatives, IC_50_ values ranged from 497.71 µM (**AB1**) to below 100 µM (**AB6**), indicating varying levels of toxicity toward healthy cells (Table 2). Compound **AB6** exhibited the highest level of toxicity to BJ cells, with an IC_50_ value of less than 100 μM, suggesting the possibility of off-target effects. In contrast, the remaining thiosemicarbazide derivatives showed moderate to low toxicity, with IC_50_ values ranging from 302.68 μM (**AB5**) to 497.71 μM (**AB1**), which was close to the maximum tested concentration. The toxicity of thiosemicarbazides toward healthy BJ fibroblasts increases depending on the chlorine atom’s location in the chlorophenyl substituent and the quantity of chlorine atoms. The cytotoxicity is augmented as chlorine atoms occupy various locations on the phenyl ring. Furthermore, an augmentation in the quantity of chlorine atoms in the chlorophenyl moiety is associated with an increased level of toxicity. This indicates that both the spatial configuration and the extent of halogenation substantially affect the biological activity of these chemicals.

The anti-cancer activity screening of thiosemicarbazides revealed that **AB1** showed the lowest activity, with IC_50_ values exceeding the highest evaluated concentration of 500 µM in seven out of the nine tested cancer cell lines. The only two cell lines that achieved an IC_50_ value in the tested concentration range were LNCaP (252.14 µM) and G-361 (369.37 µM). **AB2** exhibited lower IC_50_ values, with the greatest activity also in LNCaP (108.14 μM) and G-361 (222.74 μM) cell lines. Similar results were obtained with the other investigated thiosemicarbazides (**AB3**–**AB6**), again showing the lowest IC_50_ and therefore the greatest anti-tumor activity in the LNCaP and G-361 lines. Despite the fact that the lowest IC_50_ values for the above-mentioned cell lines were observed in the case of **AB6**, this compound was excluded from further studies due to the low selectivity index (SI) (<1), indicating high toxicity of **AB6** against normal cells. The selectivity index (SI) is a measure of a compound’s ability to target cancer cells over normal cells, calculated by dividing the IC_50_ value for normal cells (such as fibroblasts) by the IC_50_ value for cancer cells, with higher SI values indicating greater selectivity for cancer cells. Out of all investigated thiosemicarbazides, the highest SI values were obtained for **AB2** in the G-361 melanoma cell line (2.04) and LNCaP prostate cancer cell line (4.19). Therefore, the above-mentioned compound and cell lines were selected for further in-depth studies.

This study investigated the antitumor efficacy of chlorophenyl-containing thiosemicarbazides, which have demonstrated activity across several cancer cell lines, especially G-361 melanoma cells and LNCaP prostate cancer cells. Our team’s previously published research papers have demonstrated the anticancer potential of thiosemicarbazides in various cancer types, including the MKN74 gastric cancer cell line [26] and G-361 malignant melanoma cells [25]. Research conducted by Malki et al. demonstrated that 4-(4-chlorophenyl)thiosemicarbazides displayed significant anticancer activity against MCF-7 breast cancer cells [60]. Many other studies have shown that compounds possessing a privileged thiosemicarbazide scaffold are potential anticancer agents i.a., acridine thiosemicarbazides derivatives exhibited significant anticancer activity against acute lymphoblastic leukemia MT-4 cells [61] and chalcone thiosemicarbazide derivatives have antiproliferative activity against HepG2 human hepatocellular liver carcinoma [62]. Therefore, thiosemicarbazides require additional investigation about their anticancer potential.

#### 2.4.2. Cell Cycle Analysis and Apoptosis Detection

**Figure 8:**
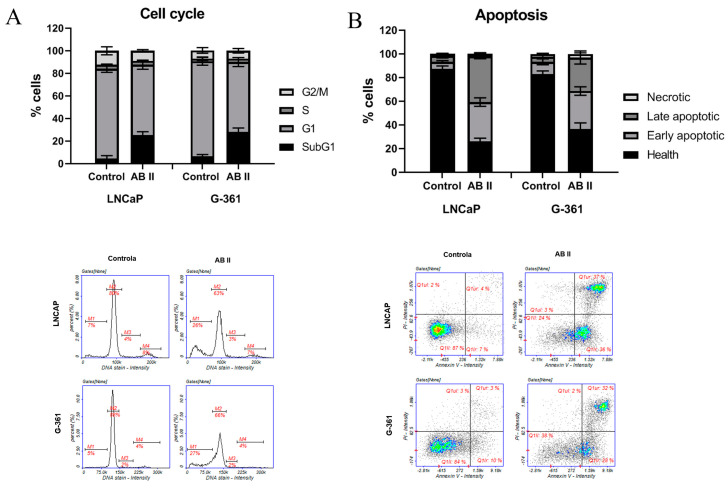
(**A**) Analysis of cell cycle of LNCaP prostate cancer cell line and G-361 melanoma cell line. The cells were treated for 48 h with **AB2** in concentration approximate to IC_50_ value. Results of three independent experiments are shown as mean ± SD. Representative LNCaP and G-361 cell cycle histograms (M1—subG1, M2—G1, M3—S, M4—G2/M phase) are presented below the graph. (**B**) Cell apoptosis/necrosis of LNCaP prostate cancer cell line and G-361 melanoma cell line treated for 48 h with **AB2** in concentration approximate to IC_50_ value. Results of three independent experiments are shown as mean ± SD. Representative LNCaP and G-361 cell cycle histograms (Q1II: live cells; Q1Ir: early apoptotic cells; Q1ur: late apoptotic cells; Q1uI: necrotic cells) are presented below the graph.

To further evaluate the anticancer activity of thiosemicarbazide derivative **AB2** in G-361 and LNCaP cell lines, cell cycle analysis by image cytometry and detection of apoptosis using annexin V and propidium iodide was performed. Analyses of the action of **AB2** on the distribution of cells in different phases of the cell cycle indicated a significant increase in the population of cells in the sub-G1 phase compared to the control cultures, in both tested prostate and melanoma cells. This population increased from 7% and 5% in control cells to 26% and 27% after exposure to **AB2** in LNCaP and G-361 cells, respectively (Figure 8A). The increase in the cell population in the sub-G1 phase indicates the presence of apoptosis or necrosis in the investigated cells and not a significant inhibition of cell proliferation, i.e., a cytostatic effect. In another study evaluated by our research team, thiosemicarbazide derivatives PK2, PK6, and PK9 also caused an increase in a population of cells in the sub-G1 phase on G-361 melanoma cells [25]. Surprisingly, another research established that the other thiosemicarbazides derivatives compounds 1 and 2 caused the arrest of the cell cycle in the S and G2 phases on the MKN74 gastric cell line [26].

Following treatment with **AB2**, a significant increase in the population of cells in both early and late apoptosis was observed in both the LNCaP and G-361 cell lines. In prostate cancer cells, the percentage of early apoptotic cells substantially elevated after **AB2** treatment compared with the control group, indicating the initial activation of the apoptotic pathway. A similar trend was seen in the melanoma cell line, where the proportion of early apoptotic cells also rose significantly. The percentage of cells in the early phase of apoptosis is approximately 36% and 28% in LNCaP and G-361 cells, respectively (Figure 8B). At the same time, the population of cells in late apoptosis increased in both cell lines, implying a progression of the apoptotic process after **AB2** exposure. This increase in late apoptotic cells was particularly pronounced in LNCaP cells (37%).

#### 2.4.3. Analysis of Gene Expression Related to Antioxidant Defense, Cell Cycle Regulation, DNA Damage Sensing and Repair and Apoptosis

**Figure 9:**
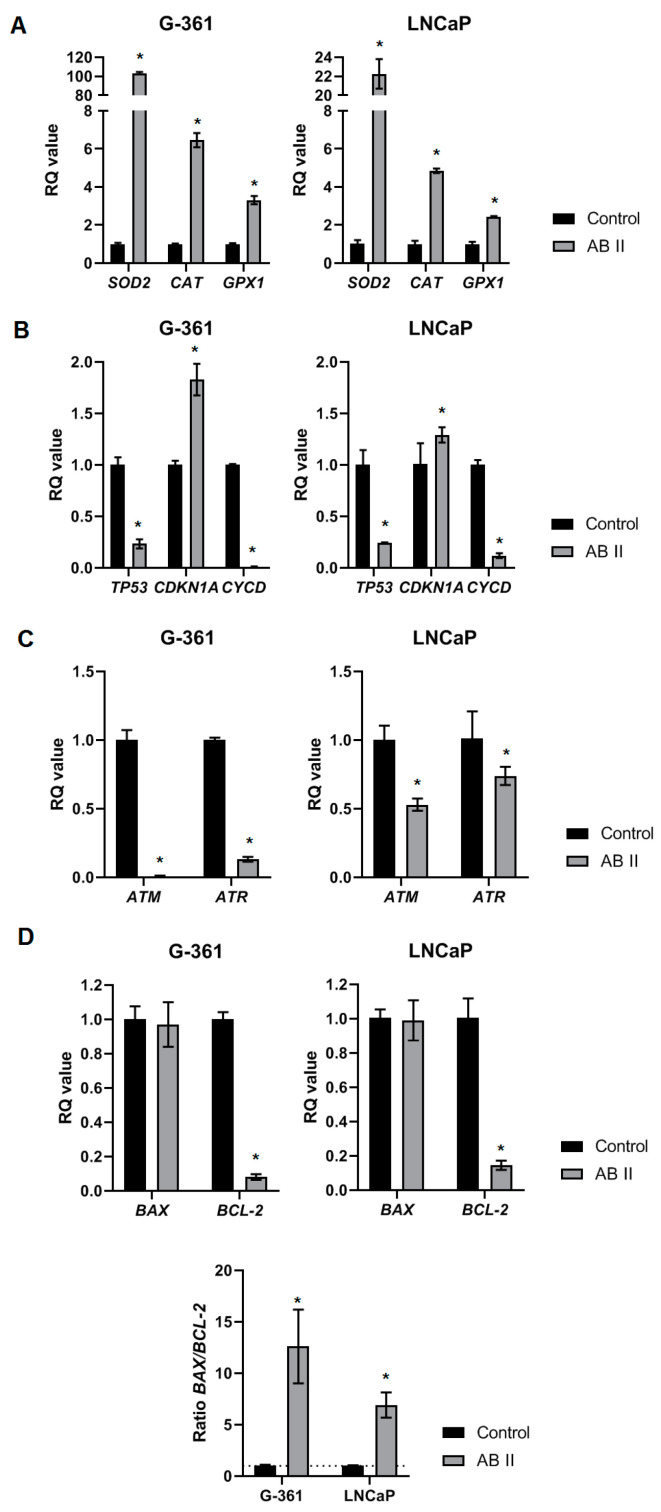
Relative mRNA expression level of genes related to (**A**) antioxidant defense, (**B**) cell cycle regulation, (**C**) DNA repair and (**D**) apoptosis in G-361 melanoma cell line and LNCaP prostate cancer cell line after 24h treatment with **AB2** in concentration approximate to IC_50_ value. *BACT* and *RNA18SN5* were used as reference genes. Results have been determined as RQ values and are expressed as means ± SDs. Compound vs. Control * *p* < 0.05.

An analysis was conducted to examine the impact of the thiosemicarbazide **AB2** on LNCaP prostate cancer cells and G-361 melanoma cells, focusing on the expression of genes associated with oxidative stress, cell cycle regulation, DNA repair, and apoptosis. Superoxide dismutase (SOD2), catalase (CAT), and glutathione peroxidase 1 (GPX1) are critical antioxidant enzymes that protect cells from oxidative stress by neutralizing reactive oxygen species (ROS) and mitigating potential cellular damage [63]. The expression of the *SOD2* gene increased significantly following **AB2** exposure in both cell lines, with G-361 cells exhibiting over a 100-fold rise and LNCaP cells showing more than a 20-fold increase compared to control cultures, indicating a robust activation of antioxidant defense mechanisms (Figure 9A). **AB2** significantly increased the expression of *CAT* and *GPX1* in both tested cell lines. *CAT* showed an approximately 8-fold increase in G-361 and a 6-fold increase in LNCaP, while *GPX1* exhibited a moderate but significant rise in both cell lines. In the next group of analyzed genes, **AB2** treatment resulted in a significant downregulation of *TP53* expression in both tested cell lines, suggesting a potential suppression of the p53-mediated DNA damage response pathway [64]. *CDKN1A* (p21) expression was significantly upregulated in assessed melanoma and prostate cancer cells after **AB2** treatment. This indicates heightened activation of cell cycle arrest mechanisms, given that p21 acts as an important inhibitor of cyclin-dependent kinases essential for cell cycle progression [65]. Conversely, *CYCD* (cyclin D), which facilitates the transition from G1 to S phase, was notably downregulated in both cell lines. This suggests that **AB2** may impede cell proliferation by inhibiting cyclin D-mediated progression through the cell cycle. The contrasting alterations in *CDKN1A* and *CYCD* indicate a transition toward cell cycle arrest in response to **AB2** (Figure 9B). The next assessed panel of genes includes those involved in DNA damage sensing and repair, specifically *ATM* and *ATR*. ATM and ATR are fundamental components of the DNA damage response, responsible for the detection and repair of double-strand and single-strand DNA breaks, respectively [66]. Both of the evaluated genes’ expression is downregulated after **AB2** treatment in both tested cell lines, but a decrease in expression of the above-mentioned genes is stronger in the G-361 cell line (Figure 9C). The decrease in their expression indicates that **AB2** could interfere with the ability of cells to accurately detect and repair DNA damage.

The last assessed panel of genes includes genes involved in the process of apoptosis. BAX is a protein that enhances apoptosis, a natural to eliminate damaged or cancer cells. On the other hand, BCL-2 prevents apoptosis, helping cancer cells survive and resist therapy [67]. After **AB2** treatment, the expression levels of the pro-apoptotic gene *BAX* did not differ from the control cultures in both G-361 and LNCaP cell lines. The expression of the anti-apoptotic gene *BCL-2* was significantly downregulated in both cell lines. The decrease in *BCL-2* expression, along with stable *BAX* levels, led to a higher *BAX/BCL*-2 ratio in comparison to the control. The increased *BAX/BCL-2* ratio indicates a transition toward pro-apoptotic signaling, suggesting that **AB2** may augment apoptotic potential in these cell lines by diminishing the protective role of *BCL-2*, hence promoting cell death.

Gene expression analysis enhances the understanding of the possible mechanism of action of thiosemicarbazide **AB2** regarding cancer cells. The elevated expression of *CAT*, *SOD2*, and *GPX1* genes indicates the occurrence of oxidative alterations in cancer cells induced by the **AB2**. Oxidative stress induced by Reactive Oxygen Species (ROS) results in DNA damage, requiring an efficient functioning of the DNA damage repair mechanism (DDR) within the cell. In response to oxidative stress, key DNA damage response regulators, including ATM and ATR, should be activated [68,69]. The experiment reveals a notable reduction in *ATM* and *ATR* expression, indicating that the **AB2** disrupts the DDR pathway. The decreased expression of *ATM* and *ATR* likely results in an accumulation of DNA damage, contributing to heightened genomic instability and possibly playing an essential part in the pro-apoptotic effects of **AB2** [70]. The presence of apoptotic cells in the Sub-G1 phase in cell cycle analysis may be a consequence of irreversible DNA damage that triggered cell cycle arrest and subsequent activation of apoptotic pathways in response to the inability of cells to repair this damage. This investigation confirmed the presence of apoptotic cells in response to **AB2**, revealing an increased population of both early and late apoptotic cells in both of the investigated cell lines. In summary, biological studies suggest a possible mechanism of action for thiosemicarbazide **AB2** in LNCaP prostate cancer cells and G-361 melanoma cells, associated with the concurrent induction of oxidative stress and disruption of DNA repair, leading to the initiation of apoptosis in cancer cells. Nonetheless, additional comprehensive research is required to identify the precise molecular target of the **AB2**.

### 2.5. Molecular Docking

Taking into account the results of biological activity obtained in in vitro tests, it is highly probable that a potential molecular target may be topoisomerase IIα, which is responsible for the control of the topological state of the DNA strand [71]. Topoisomerase II occurs in two main isoforms: alpha (IIα) and beta (IIβ), but only isoform α is crucial for cell proliferation [72]. Moreover, the expression of topoisomerase IIα has been associated with the progression of prostate cancer [71,73]. Therefore, it was selected by our team as a potential target for molecular docking. The crystal structure of human topoisomerase II alpha in complex with DNA and etoposide as a molecular target for investigated ligands was downloaded from Protein Data Bank (PDB ID: 5GWK [74]).

**Figure 10:**
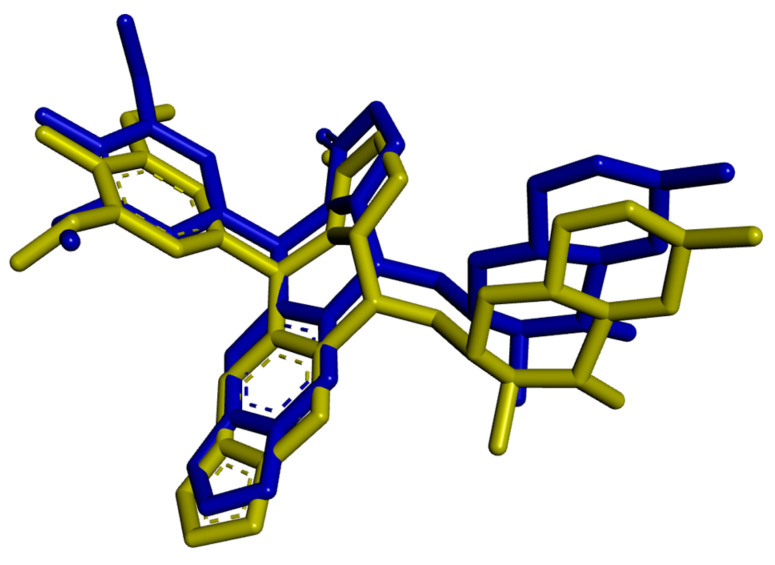
Superimpose of native ligand from protein (PDB ID: 5GWK) [74] (yellow) and redox (blue) with RMS = 1.022 Å. (Only non-hydrogen atoms are shown).

For the validation of the docking method, redocking of the native ligand was performed, which gave the best match to the binding site with a ChemScore value of 112.58 and RMS value of 1.022 Å, indicating the correctness of the docking method (Figure 10).

Molecular docking was performed only for thiosemicarbazide derivatives, due to the low cytotoxicity toward cancer cells of semicarbazide derivatives. Molecular docking study showed that all tested ligands bind to the active site of the indicated molecular target. The ChemScore scoring function values and amino acids involved in hydrogen bonds are presented in Table 3.

The ChemScore values for all designed ligands are within a narrow range, with the highest value of 75.42 obtained for **AB2** and the lowest value of 70.91 estimated for **AB1**. These values are significantly lower than those obtained for the native ligand (112.58). However, etoposide is an officially approved chemotherapeutic drug and eukaryotic topoisomerase II poison [72]. Therefore the best result in molecular docking is a confirmation of its high potential.

**Figure 11:**
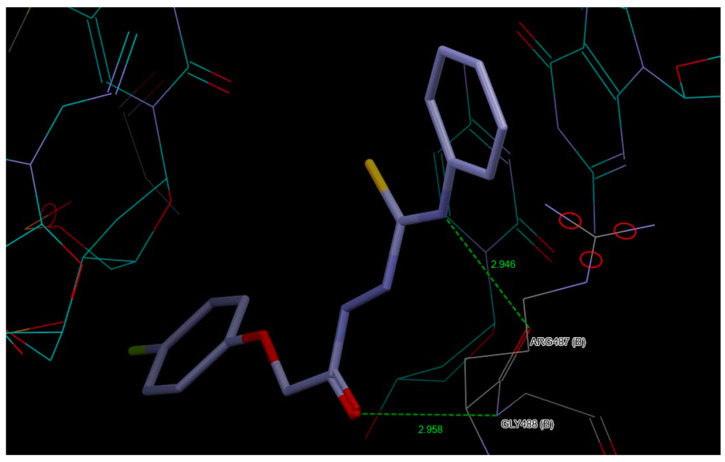
Interactions of **AB2** with the active site of topoisomerase IIα.

It is worth noting that the highest ChemScore value was obtained for compound **AB2**, which correlates with the highest activity obtained in biological studies for this compound. Figure 11 shows the interactions of **AB2** with the active site of topoisomerase IIα (interactions for other ligands are presented in Figures S25–S29, Supplementary Materials).

The most active ligand **AB2** forms a hydrogen bond between the aromatic hydrogen atom of the 4-fluorophenoxyacetyl moiety and the nitrogen atom of the DA12(D) DNA chain and this hydrogen bond is also present for **AB3, AB4** and **AB6**. **AB5** is the only compound that interacts with the molecular target via two chlorine atoms of the phenyl ring derived from isothiocyanate.

However, the **AB1** compound, unlike others, interacts with the target molecule via hydrogen bonds formed by the oxygen of the carbonyl group and the nitrogen of the amino group of the thiosemicarbazide chain. For the **AB4** compound, the formation of one hydrogen bond is observed by the amino group of the thiosemicarbazide chain, while for the **AB3** and **AB6** compounds, the thione group from the thiosemicarbazide chain participates in the formation of the hydrogen bond.

## 3. Experimental

### 3.1. Chemical Reagents and General Method

The chemicals and reagents were commercially available and were purchased from Sigma-Aldrich (Sigma-Aldrich, Saint Louis, MO, USA), AlfaAesar (Haverhill, MA, USA), POCH (Haverhill, MA, USA), Fluorochem (Fluorochem, GBR) and used without further purification. The crude compounds were purified by crystallization from methanol. ^1^H and ^13^C NMR spectra were performed on a Bruker Avance 600 spectrometer (Bruker BioSpin GmbH, Rheinstetten, Germany). TMS (tetramethylsilane) was used as an internal standard and DMSO-*d_6_* was used to dissolve the samples. The chemical shifts (d) are indicated in ppm. For accurate mass measurements, an Agilent Technologies 1290 liquid chromatograph coupled to an Agilent Technologies 6550 iFunnel Q-TOF LC/MS was employed. Flow injection analyses utilized a mobile phase of 0.1% formic acid in water:acetonitrile (50:50 *v*/*v*) with a flow rate of 0.5 mL/min. Compounds were ionized by electrospray and detected in positive polarity. Mass correction using two reference ions of *m*/*z* 121.0509 and 922.0098 was enabled. Instrument control, data acquisition, and analysis were conducted using Agilent MassHunter Workstation B.07 software. For all compounds single-charged protonated ions were observed.

### 3.2. General Procedure for the Synthesis of 1-(4-Fluorophenoxyacetyl)-4-substituted Thiosemicarbazide (***AB1***–***AB6***)

2-(4-Fluorophenoxy)acetohydrazide (0.5 g, 2.71 mmol) and the appropriate isothiocyanate (dissolved in approximately 15–20 mL of methanol) were mixed in a round-bottomed flask.

**Figure 12:**
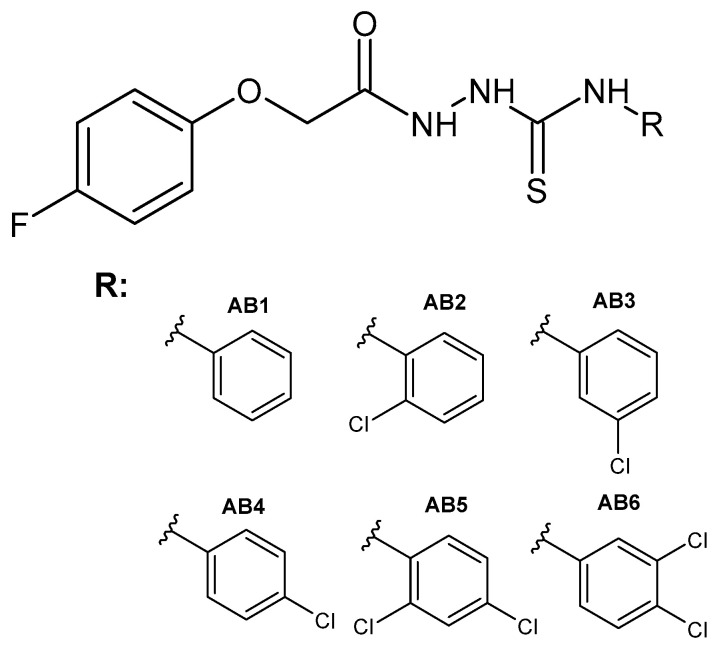
The general formula of the designed thiosemicarbazides **AB1**–**AB6**.

The solution was heated for an hour and then cooled to precipitate. The precipitate was filtered off, recrystallized from methanol. Figure 12 shows the general formula of 4-fluorophenoxyacetylthiosemicarbazides **AB1**–**AB6**. Spectroscopic data of the obtained compounds can be found in the Supplementary Material.

1-(4-Fluorophenoxy)-4-(phenyl)acetylthiosemicarbazide (**AB1**)

Molecular formula: C_15_H_14_FN_3_O_2_S (319.36g/mol), white solid, yield 80% (0.69g); m.p. 179–181 °C. ^1^H NMR (600 MHz, DMSO-*d_6_*): 4.62 (s, 2H, CH_2_); 7.03–7.42 (m, 9H, CH_ar_), 9.68 (s, 2H, NH); 10.28 (s, 1H, NH). ^13^C NMR (300 MHz, DMSO): 67, 116, 116, 125.70–126.51 (^1^*J*C–F = 243 Hz), 129, 140, 1554, 1564, 158, 168, 181. ^19^F NMR (400 MHz, CFCL_3_-*d_8_*): −122.8 (m, 1F). IR γ_max_ (cm^−1^): 3227 (NH), 3099 (NH), 2934 (NH), 1701 (C=O), 1201 (C=S). LC-QTOF MS (*m*/*z*): calculated monoisotopic mass: 319.0790, measured monoisotopic mass: 320.0862.

1-(2-Chlorophenyl)-4-(4-fluorophenoxy)acetylthiosemicarbazide (**AB2**)

Molecular formula: C_15_H_13_ClFN_3_O_2_S (353.79 g/mol), white solid yield 73% (0.70 g); m.p. 166–168 °C. ^1^H NMR (600 MHz, DMSO-*d_6_*): 4.63 (s, 2H, CH_2_); 6.96–7.50 (m, 8H, CH_ar_); 9.56 (s, 1H, NH); 9.82 (s, 1H, NH); 10.36 (s, 1H, NH) ppm. ^13^C NMR (300 MHz, DMSO): 67, 116, 116, 128, 129, 130, 131.54–131.99 (^1^*J*C–F = 135 Hz), 137, 1554, 156, 158, 167, 182. ^19^F NMR (400 MHz, CFCL_3_-*d_8_*): −122.9 (m, 1F). IR γ_max_ (cm^−1^): 3290 (NH), 3228 (NH), 3130 (NH), 1664 (C=O), 1217 (C=S). LC-QTOF MS (*m*/*z*): calculated monoisotopic mass: 353.0401, measured monoisotopic mass: 354.0472.

1-(3-Chlorophenyl)-4-(4-fluorophenoxy)acetylthiosemicarbazide (**AB3**)

Molecular formula: C_15_H_11_ClFN_3_O_2_S (353.79 g/mol), white solid yield 53% (0.51 g), m.p. 163–165 °C. ^1^H NMR (600 MHz, DMSO-*d_6_*): 4.63 (s, 2H, CH_2_); 7.04–7.60 (m, 8H, CH_ar_); 9.80 (s, 2H, 2xNH); 10.32 (s, 1H, NH) ppm. ^13^C NMR (300 MHz, DMSO): 67, 116, 116, 124.66–125.37 (^1^*J*C–F = 213 Hz), 126, 130, 133, 141, 154, 156, 158, 168, 181. ^19^F NMR (400 MHz, CFCL_3_-*d_8_*): −122.8 (m, 1F). IR γ_max_ (cm^−1^): 3265 (NH), 3186 (NH), 3020 (NH), 1684 (C=O), 1210 (C=S). LC-QTOF MS (*m*/*z*): calculated monoisotopic mass: 353.0401, measured monoisotopic mass: 354.0473.

1-(4-Chlorophenyl)-4-(4-fluorophenoxy)acetylthiosemicarbazide (**AB4**)

Molecular formula: C_15_H_13_ClFN_3_O_2_S (353.79 g/mol), white solid yield 42% (0.40 g), m.p. 168–169 °C. ^1^H NMR (600 MHz, DMSO-*d_6_*): 4.62 (s, 2H, CH_2_); 7.05–7.47 (m, 8H, CH_ar_); 9.79 (s, 1H, NH); 9.77 (s, 1H, NH); 10.30 (s, 1H, NH) ppm. ^13^C NMR (300 MHz, DMSO): 67, 116, 116, 128.07–128.46 (^1^*J*C–F = 117 Hz), 129, 138, 154, 155, 158, 167, 181. ^19^F NMR (400 MHz, CFCL_3_-*d_8_*): −122.8 (m, 1F). LC-QTOF MS (*m*/*z*): calculated monoisotopic mass: 353.0401, measured monoisotopic mass: 354.0470.

1-(2,4-Dichlorophenyl)-4-(4-fluorophenoxy)acetylthiosemicarbazide (**AB5**)

Molecular formula: C_15_H_12_Cl_2_FN_3_O_2_S (388.24 g/mol), white solid yiled 63% (0.66 g), m.p. 181–183 °C. ^1^H NMR (600 MHz, DMSO-*d_6_*): 4.62 (s, 2H, CH_2_); 7.02–7.70 (m, 7H, CH_ar_); 9.59 (s, 1H, NH); 9.91 (s, 1H, NH); 10.37 (s, 1H, NH) ppm.^13^C NMR (300 MHz, DMSO): 67, 116, 116, 128, 129, 131, 132.12–132.86 (^1^*J*C–F = 222 Hz), 136, 1554, 157, 158, 168, 182. ^19^F NMR (400 MHz, CFCL_3_-*d_8_*): −122.8 (m, 1F). IR γ_max_ (cm^−1^): 3285 (NH), 3235 (NH), 3116 (NH), 1667 (C=O), 1204 (C=S). LC-QTOF MS (*m*/*z*): calculated monoisotopic mass: 387.0011, measured monoisotopic mass: 3.88.0082.

1-(3,4-Dichlorophenyl)-4-(4-fluorophenoxy)acetylthiosemicarbazide (**AB6**)

Molecural formula: C_15_H_12_Cl_2_FN_3_O_2_S (388.24 g/mol), white solid yield 76% (0.80 g), m.p. 176–178 °C. ^1^H NMR (600 MHz, DMSO-*d_6_*): 4.63 (s, 2H, CH_2_); 7.03–7.81 (m, 7H, CH_ar_); 9.87 (s, 2H, 2xNH); 10.33 (s, 1H, NH) ppm. ^13^C NMR (300 MHz, DMSO): 67, 116, 116, 126, 127, 130.35–131.06 (^1^*J*C–F = 213 Hz), 139, 154, 155, 158, 168, 181. ^19^F NMR (400 MHz, CFCL_3_-*d_8_*): −122.7 (m, 1F). IR γ_max_ (cm^−1^): 3287 (NH), 3252 (NH), 3102 (NH), 1666 (C=O), 1212 (C=S). LC-QTOF MS (*m*/*z*): calculated monoisotopic mass: 387.0011, measured monoisotopic mass: 388.0082.

### 3.3. General Procedure for the Synthesis of 1-(4-Fluorophenoxyacetyl)-4-substituted Semicarbazide (***AB7***–***AB12***)

2-(4-Fluorophenoxy)acetohydrazide (0.5 g, 2.71 mmol) and the appropriate isocyanate (dissolved in approximately 15–20 mL of ethanol) were mixed in a round-bottomed flask.

**Figure 13:**
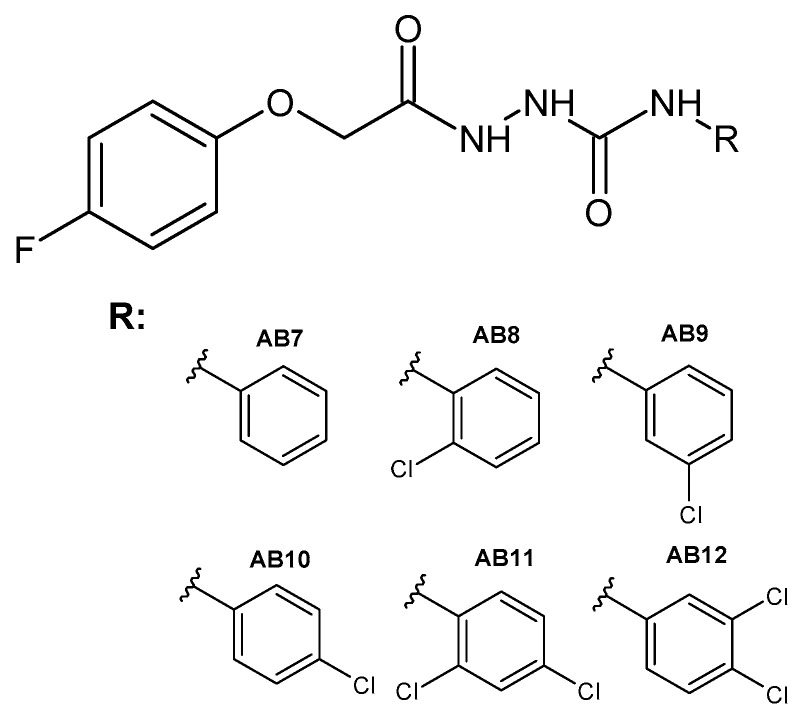
The general formula of the designed semicarbazides **AB7**–**AB12**.

The solution was heated for an hour and then cooled to precipitate. The precipitate was filtered off and recrystallized from ethanol. Figure 13 shows the general formula of 4-fluorophenoxyacetylsemicarbazides **AB7**–**AB12**. Spectroscopic data of the obtained compounds can be found in the Supplementary Material.

1-(4-Fluorophenoxy)-4-(phenyl)acetylsemicarbazide (**AB7**)

Summary formula: C_15_H_14_FN_3_O_3_ (303.29 g/mol), white solid yield 37% (0.30 g), m.p. 222–223 °C. ^1^H NMR (600 MHz, DMSO-*d_6_*): 4.60 (s, 2H, CH_2_); 6.96–7.44 (m, 9H, CH_ar_); 8.14 (s, 1H, NH); 8.75 (s, 1H, NH); 10.00 (s, 1H, NH) ppm. ^13^C NMR (300 MHz, DMSO): 67, 116, 119.05–119.72 (^1^*J*C–F = 231 Hz), 122, 129, 140, 154, 156, 159, 168, 172. ^19^F NMR (400 MHz, CFCL_3_-*d_8_*): −122.8 (m, 1F). IR γ_max_ (cm^−1^): 3268 (NH), 3020 (NH), 2920 (NH), 1682 (C=O), 1644 (C=O). LC-QTOF MS (*m*/*z*): calculated monoisotopic mass: 303.1019, measured monoisotopic mass: 304.1085.

1-(2-Chlorophenyl)-4-(4-fluorophenoxy)acetylsemicarbazide (**AB8**)

Summary formula: C_15_H_13_ClFN_3_O_3_ (337.73 g/mol), white solid yield 26% (0.23 g), m.p. 225–227 °C. ^1^H NMR (600 MHz, DMSO-*d_6_*): 4.61 (s, 2H, CH_2_); 7.00–8.05 (m, 8H, CH_ar_); 8.27 (s, 1H, NH); 8.83 (s, 1H, NH); 10.134 (s, 1H, NH) ppm. ^13^C NMR (300 MHz, DMSO): 67, 116, 121.42–122.24 (^1^*J*C–F = 246 Hz), 124, 128, 129, 136, 154, 155, 156, 158, 168, 172. ^19^F NMR (400 MHz, CFCL_3_-*d_8_*): −122.7 (m, 1F). IR γ_max_ (cm^−1^): 3308 (NH), 3258 (NH), 2913 (NH), 1679 (C=O), 1657 (C=O). LC-QTOF MS (*m*/*z*): calculated monoisotopic mass: 337.0629, measured monoisotopic mass: 338.0696.

1-(3-Chlorophenyl)-4-(4-fluorophenoxy)acetylsemicarbazide (**AB9**)

Summary formula: C_15_H_13_ClFN_3_O_3_ (337.73 g/mol), white solid yield 28% (0.25 g), m.p. 212–214 °C. ^1^H NMR (600 MHz, DMSO-*d_6_*): 4.60 (s, 2H, CH_2_); 7.01–7.67 (m, 8H, CH_ar_); 8.32 (s, 1H, NH); 8.99 (s, 1H, NH); 10.01 (s, 1H, NH) ppm. ^13^C NMR (300 MHz, DMSO): 67, 116, 116.99–117.94 (^1^*J*C–F =285) 122, 130, 133, 141, 154, 155, 156, 158, 168, 172. ^19^F NMR (400 MHz, CFCL_3_-*d_8_*): −122.8 (m, 1F). IR γ_max_ (cm^−1^): 3236 (NH), 3060 (NH), 2909 (NH), 1679 (C=O), 1642 (C=O). LC-QTOF MS (*m*/*z*): calculated monoisotopic mass: 337.0629, measured monoisotopic mass: 338.0695.

1-(4-Chlorophenyl)-4-(4-fluorophenoxy)acetylsemicarbazide (**AB10**)

Summary formula: C_15_H_13_ClFN_3_O_3_ (337.73 g/mol), white solid yield 34% (0.31 g), m.p. 212–214 °C. ^1^H NMR (600 MHz, DMSO-*d_6_*): 4.59 (s, 2H, CH_2_); 7.00–7.50 (m, 8H, CH_ar_); 8.24 (s, 1H, NH); 8.92 (s, 1H, NH); 10.00 (s, 1H, NH) ppm. ^13^C NMR (300 MHz, DMSO): 67, 116, 120, 125.50–126.30 (^1^*J*C–F =240), 128, 139, 154, 156, 158, 168, 172. ^19^F NMR (400 MHz, CFCL_3_-*d_8_*): −122.8 (m, 1F). IR γ_max_ (cm^−1^): 3265 (NH), 3005 (NH), 2910 (NH), 1677 (C=O), 1648 (C=O). LC-QTOF MS (*m*/*z*): calculated monoisotopic mass: 337.0629, measured monoisotopic mass: 338.0696.

1-(2,4-Dichlorophenyl)-4-(4-fluorophenoxy)acetylsemicarbazide (**AB11**)

Summary formula: C_15_H_12_Cl_2_FN_3_O_3_ (372.18 g/mol), white solid yield 58% (0.52 g), m.p. 211–213 °C. ^1^H NMR (600 MHz, DMSO-*d_6_*): 4.61 (s, 2H, CH_2_); 6.9–8.08 (m, 7H, CH_ar_); 8.36 (s, 1H, NH); 8.87 (s, 1H, NH); 10.14 (s, 1H, NH) ppm. ^13^C NMR (300 MHz, DMSO): 67, 116, 122.11–122.95 (^1^*J*C–F =252), 126, 128, 129, 135, 154, 1554, 156, 158, 168, 172. ^19^F NMR (400 MHz, CFCL_3_-*d_8_*): −122.7 (m, 1F). IR γ_max_ (cm^−1^): 3308 (NH), 3251 (NH), 2914 (NH), 1681 (C=O), 1655 (C=O). LC-QTOF MS (*m*/*z*): calculated monoisotopic mass: 371.0239, measured monoisotopic mass: 372.0306.

1-(3,4-Dichlorophenyl)-4-(4-fluorophenoxy)acetylsemicarbazide (**AB12**)

Summary formula: C_15_H_12_Cl_2_FN_3_O_3_ (372.18 g/mol), white solid yield 40% (0.39 g), m.p. 232–234 °C. ^1^H NMR (600 MHz, DMSO-*d_6_*): 4.59 (s, 2H, CH_2_); 7.01–7.86 (m, 7H, CH_ar_); 8.42 (s, 1H, NH); 9.11 (s, 1H, NH); 10.02 (s, 1H, NH) ppm. ^13^C NMR (300 MHz, DMSO): 67, 116, 118.68–119.68 (^1^*J*C–F =300), 123, 130, 131, 140, 154, 155, 156, 158, 168, 172. ^19^F NMR (400 MHz, CFCL_3_-*d_8_*): −122.8 (m, 1F). IR γ_max_ (cm^−1^): 3256 (NH), 3025 (NH), 2910 (NH), 1679 (C=O), 1645 (C=O). LC-QTOF MS (*m*/*z*): calculated monoisotopic mass: 371.0239, measured monoisotopic mass: 372.0304.

### 3.4. X-Ray Structure Determinations

The experimental and crystal data of **AB2** and **AB5** are presented in Table 4.

Both structures were solved using direct methods with SHELXS-2013/1 [76] and refined by full matrix least squares with SHELXL-2014/7 [76]. The H atoms connected to N atoms were located on differential electron density maps and refined freely. The remaining H atoms positioned geometrically were treated as riding on their parent C atoms with C-H distances of 0.93 Å (aromatic) and 0.97 Å (CH_2_). All H atoms were refined with isotropic displacement parameters taken as U_iso_(H) = 1.5*U*_eq_(C,N). All calculations were performed using WINGX version 2014.1 package [77]. CCDC- 2423249 (**AB2**) and CCDC- 2423250 (**AB5**) contain the supplementary crystallographic data for this paper. These data can be obtained free of charge at www.ccdc.cam.ac.uk/conts/retrieving.html (URL accessed on 27 March 2025) [or from the Cambridge Crystallographic Data Centre (CCDC), 12 Union Road, Cambridge CB2 1EZ, UK; fax: +44(0) 1223 336,033; email: deposit@ccdc.cam.ac.uk].

### 3.5. ADMET Analysis

BOILED-Egg [78] and SwissADME service (Swiss Institute of Bioinformatics 2021): A free web tool to evaluate pharmacokinetics, drug likeness, and the medicinal chemistry friendliness of small molecules [79] was used to predict absorption in the gastrointestinal tract and ADME analysis, respectively. Potential toxicity was assessed using the ProTOX II service [80].

### 3.6. Biological Evaluation

#### 3.6.1. Cell Culture and Treatment

The research was conducted on one primary cell line: BJ (fibroblast) and four types of cancerous ones: three prostate cancer cell lines (PC-3, DU-145, LNCaP); 3 Melanoma cell lines (MEL-28, A375, G-361); 2 breast cancer cell lines (MCF-7, MDA-MB-23) and one lung cancer cell line (NCI-H1563). All cell lines were obtained from the American Type Culture Collection (ATCC, Manassas, VA, USA) and were grown in media (Corning, New York, NY, USA) according to ATCC guidelines. The culture media was enriched with 10% heat-inactivated fetal bovine serum (Life Technologies, Carlsbad, CA, USA) and antibiotics: penicillin (100 units) and streptomycin (100 μg/mL) from Sigma-Aldrich (St. Louis, MO, USA). The cells were incubated at 37 °C with 5% CO_2_ in air.

The cytotoxic analysis of thiosemicarbazides (TSC) (**AB1**–**AB6**) and semicarbazides (SC) (**AB7**–**AB12**) was conducted in various concentrations. In the case of TSC, it was from 500 to 100 µM, while for SC, it was from 100 to 1 µM (due to its low solubility). The cells were treated with compounds or DMSO as a vehicle in control cultures for 48h. IC_50_ values were determined using the AAT Bioquest IC_50_ calculator (AAT Bioquest, Inc., Pleasanton, CA, USA, Quest Graph™ EC_50_ Calculator. https://www.aatbio.com/tools/ec50-calculator) (accessed on 7 October 2024). According to the lowest obtained IC_50_ value and the highest Selective Index of tested compounds on selected cancer cell lines, the **AB2** (thiosemicarbazide) was chosen for further depth research on LNCaP and G-361 cell lines.

#### 3.6.2. MTT Assay

The cytotoxic activity of the thiosemicarbazides and semicarbazides was measured using the MTT colorimetric assay (MTT Cell Proliferation Assay Kit—Invitrogen, Waltham, MA, USA). The cells were seeded in the 96-well plates at a density of 1.5 × 10^5^ cells/mL. After reaching 70–80% of confluence, the tested compounds in a wide range of concentrations were added. Plates were incubated for 48 h at 37 °C in 5% CO_2_ air. Then, orange tetrazolium salt (3-(4,5-dimethylthiazol-2-yl)-2,5-diphenyltetrazolium bromide)—MTT reagent (0.5 mg/mL) dissolved in PBS was added to each well for 4 h. The MTT solution was then removed from above the water-insoluble purple formazan crystals, which were then dissolved in DMSO (dimethyl sulfoxide, 200 mL/well; POCH, Gliwice, Poland). The absorbance value of obtained solutions was measured using a PowerWave ™ microplate spectrophotometer (Bio-Tek Instruments, Winooski, VT, USA) at a wavelength of 570 nm. Each cytotoxicity assay was repeated three times in triplicates.

#### 3.6.3. Cell Cycle Analysis

Cell cycle analysis was assessed with the NucleoCounter NC-3000 (ChemoMetec, Allerod, Denmark) following the 2-Step Cell Cycle Assay protocol recommended by the manufacturer. The research was performed after 48 h incubation of prostate cancer cells (LNCaP) and melanoma cells (G-361) with **AB2** compound in a concentration corresponding to IC_50_ value. Analysis was performed in line with the previously described methodology.

#### 3.6.4. Apoptosis Detection

Detection of apoptosis in tested cells was conducted with the NucleoCounter NC-3000 (ChemoMetec, Allerod, Denmark) following the Annexin V Apoptosis Assay recommended by the manufacturer. The research was performed after 48 h incubation of prostate cancer cells (LNCaP) and melanoma cells (G-361) with **AB2** compound in a concentration corresponding to IC_50_ value. Analysis was performed in line with the previously described methodology.

#### 3.6.5. Quantitative Real-Time PCR Analysis (qRT-PCR)

The relative gene expression was quantitatively assessed using real-time PCR. The cells were seeded in 6-well plates after reaching 70–80% of confluency the compound **AB2** in a concentration corresponding to IC_50_ was added for 24 h. Next, for cell lysis the 1 mL of TRIzol™ reagent (Invitrogen, Carlsbad, CA, USA) was added. RNA was isolated according to the Chomczynski and Sacchi method [81]. The RNA was reverse transcribed using NG dART RT-PCR reagents (EURx, Gdańsk, Poland) according to the manufacturer’s instructions. Following the manufacturer’s recommendation, the qPCR reaction was conducted in triplicate in a 7500 fast real-time PCR machine (ThermoFisher, Waltham, MA, USA) using Fast SG/ROX qPCR Master Mix (2×) (EURx, Gdansk, Poland). *18SRNA* and *BACT* were used as reference genes. Gene expression was evaluated using qRT-PCR and ΔΔCt. Statistical analysis was conducted using RQ values (RQ = 2∆ΔCt).

The information on the genes and primer sequences used in the research are summarized in Table 5.

#### 3.6.6. Statistical Analysis

Statistical analysis was performed using STATISTICA 13 software (StatSoft, Kraków, Poland). Data were compared using one-way analysis of variance (ANOVA), followed by post hoc multiple comparisons with Tukey’s honest significant difference test (Tukey’s HSD test). The results are presented as mean ± SD. Statistical significance was set at a *p*-value of <0.05.

### 3.7. Molecular Docking

The crystal structure of human topoisomerase IIα in complex with DNA and etoposide was downloaded from Protein Data Bank (PDB ID: 5GWK; resolution 3.15 Å) [74]. GOLD Suite v.5.6.3 was used to perform molecular docking analysis [82]. Receptor preparation including addition of hydrogen atoms, extraction of the original ligand, and removal of water molecules from the active site of the protein was performed using GOLD according to default settings. Binding site was determined using the position of native ligand (etoposide). Redocking of native ligand (etoposide) to its binding site with RMSD values of 1.022 Å for ChemScore confirmed the correctness of the selected molecular docking parameters. In docking, it was assumed that ligands are flexible and amino acid residues of the receptor binding site are rigid. In case of simulation runs, default parameter values were used. The selection of active site atoms within 6 Å of the original ligands was chosen as default. ChemScore was chosen as the scoring function to rank the compounds to be studied. Hermes v1.8.2 was used to analyze protein–ligand interactions [82]. The superimpose of redocked etoposide with crystal ligand coordinates from the protein (PDB ID: 5GWK) [74] was analyzed by Discovery Studio Visualizer [83].

## 4. Conclusions

In this study, six new derivatives of 4-fluorophenoxyacetylthiosemicarbazide and 4-fluorophenoxyacetylsemicarbazide were obtained. X-ray structural analysis was performed for **AB2** and **AB5**, which confirmed the assumed molecular structures and the synthetic route. The compounds studied occurred in the N1-amino/S3-thione/N4-amino/N5-amino/O9-keto tautomeric form and were characterized by the occurrence of inter- and intramolecular hydrogen bonds closely related to the presented tautomeric form in the crystalline state. Hirshfeld analysis showed high similarity of fingerprints and short interactions for **AB2** and **AB5**. Clear differences are observed for the percentage of short contacts H…H and H…Cl/Cl…H. In silico analysis of pharmacokinetic parameters showed that all obtained compounds are absorbed from the gastrointestinal tract and do not cross the blood–brain barrier. There is a visible difference in lipophilicity between the group of thiosemicarbazide and semicarbazide derivatives. Furthermore, semicarbazide in in silico studies had lower toxicity than thiosemicarbazide. All designed compounds meet the main rules of druglikeness. Biological studies showed clear differences in the activity profile of thiosemicarbazide and semicarbazide. The semicarbazide were poorly soluble and had low cytotoxicity to cancer cells, which is why they were omitted from further studies. For thiosemicarbazide derivatives, activity was determined against nine cancer cell lines: prostate cancer (PC-3, DU-145, LNCaP), melanoma (SK-MEL28, A375, G-361), breast cancer (MCF-7, MDA-MB-231), and lung cancer (NCI-H1563). All tested thiosemicarbazides were characterized by moderate activity against the prostate cancer line LNCaP, the highest activity was possessed by **AB2** (IC_50_ = 108.14 μM), bearing a 2-chlorophenyl group. A large population of early apoptotic cells was observed in the LNCaP cell line treated with AB2. Moreover, gene expression analysis showed that **AB2** induced an increase in the level of *SOD2*, *CAT*, *GPX1*, *CDKN1A* and a decrease in the level of *TP53*, *CYCD*, *ATM*, *ATR*, and *BCL-2*, which indicates that the tested compound induces oxidative DNA damage and impairs the DNA repair mechanism, which leads to apoptosis. This point to the tested compounds may be potential topoisomerase IIα inhibitors, which was evaluated in molecular docking analysis. Molecular docking analysis confirmed the promising character of **AB2**, which showed the highest ChemScore value among the thiosemicarbazide tested. Molecular docking indicated that for **AB2**, the hydrogen bond formed with topoisomerase IIα is created by the hydrogen atom of the 4-fluorophenoxyacetyl group, which highlights the key role of this moiety for biological activity. It is also important to emphasize that replacing the sulfur atom with an oxygen atom in our compounds reduces the cytotoxicity toward cancer cell lines, which is a valuable clue for further potential optimization of the scaffold.

## Figures and Tables

**Table 1:** The putative prediction drug–drug interactions via cytochrome P450 (CYP) for given compounds.

Compound	CYP1A2	CYP2C19	CYP2C9	CYP2D6	CYP3A4
	Inhibitor	Inhibitor	Inhibitor	Inhibitor	Inhibitor
**AB1**	No	Yes	Yes	No	No
**AB2**	No	Yes	Yes	No	No
**AB3**	Yes	Yes	Yes	No	No
**AB4**	Yes	Yes	Yes	Yes	Yes
**AB5**	Yes	Yes	Yes	No	Yes
**AB6**	Yes	Yes	Yes	No	Yes
**AB7**	No	No	No	No	No
**AB8**	No	Yes	Yes	No	No
**AB9**	No	Yes	No	No	No
**AB10**	No	Yes	No	No	No
**AB11**	No	Yes	Yes	No	No
**AB12**	No	Yes	Yes	No	No

**Table 2:** IC_50_ values were obtained for evaluated thiosemicarbazides (**AB1**–**AB6**) based on MTT test results after 48 h treatment of BJ fibroblasts, prostate cancer cell lines, melanoma cancer cell lines, breast cancer cell lines, and lung cancer cell line.

Compound	Cell Lines									
	Fibroblast	Prostate Cancer			Melanoma			Breast Cancer		Lung Cancer
	BJ	PC-3	DU-145	LNCaP	SK-MEL28	A375	G-361	MCF-7	MDA-MB-231	NCI-H1563
IC_50_ (μM)										
**AB1**	497.71	>500	>500	252.14	>500	>500	369.37	>500	>500	>500
**AB2**	453.31	>500	>500	108.14	469.96	428.8	222.74	>500	>500	>500
**AB3**	421.67	467.13	267.67	175.42	302.11	400.17	247.55	455.76	495.32	437.25
**AB4**	312.55	355.29	297.31	257.12	321.71	398.54	265.32	342.92	421.27	293.01
**AB5**	302.68	395.24	401.2	154.31	297.33	383.66	276.39	421.31	444.78	371.59
**AB6**	<100	387.79	342.67	121.11	195.28	257.48	210.24	239.77	334.12	241.58
**Cisplatin** **(reference)**	-	52.24	64.13	31.52	25.79	18.22	12.45	22.64	38.66	2.78

**Table 3:** The results of the molecular docking of ligands **AB1**–**AB6** to topoisomerase IIα.

Compounds	ChemScore Value	Ligand–Amino Acids Interactions
**AB1**	70.91	(C=)O6…H-N(Gly488)(B) N1-H…O(=C)(Arg487)(B)
**AB2**	75.42	C33-H…N(DA12)(D)
**AB3**	74.18	(C=)S3…H-C(DC8)(E) C33-H…N(DA12)(D)
**AB4**	73.96	(C=)34…H-C(ARG487)(B) N1-H…H-C(DC8)(E) C33-H…N(DG13)(D) C33-H…N(DA12)(D)
**AB5**	74.55	C22-Cl…H-N(DG13)(D) C24-Cl…H-O(ASP463)(B)
**AB6**	72.16	(C=)S3…H-C(DC8)(E) C33-H…N(DA12)(D)

**Table 4:** Experimental details of **AB2** and **AB5** crystals.

Data Collection/Crystal Data	AB2	AB5
Diffractometer	KM4 CCD four-circle diffractometer	
Crystal size (mm)	0.40 × 0.30 × 0.20	0.30 × 0.20 × 0.10
Radiation type	Mo *K*α (λ = 0.71073 Å)	Mo *K*α (λ = 0.71073 Å)
Collected data scan	ω	ω
Absorption correction	multi-scan CrysAlisPro [75]	multi-scan CrysAlisPro [75]
Transmission factors, T_min_, T_max_	0.609/1.000	0.730/1.000
Chemical formula	C_15_H_13_ClFN_3_O_2_S	C_15_H_12_Cl_2_FN_3_O_2_S,
M_r_ (g mol^−1^)	353.79	388.24
Crystal system, space group	$\mathrm{triclinic},\;P\bar{1}$	$\mathrm{triclinic},\;P\bar{1}$
Lattice constant, a, b, c (Å), α, β, γ (°)	8.6773 (15), 9.3488 (16), 10.6905 (15), 89.835 (13), 70.387 (14), 76.972 (15)	8.7626 (8), 9.5122 (6), 10.7018 (9), 86.446 (6), 72.915 (7), 77.803 (6)
V (Å^3^)	793.4 (2)	833.40 (12)
Z	2	2
Calculated density, D_calc._ (g cm^−1^)	1.481	1.547
F(000)	364	396
Absorption coefficient, µ (mm^−1^)	0.395	0.538
Temperature (K)	293 (2)	293 (2)
No. of measured reflections	6495	5484
θ range (°)	2.029–28.744	2.190–28.517
No. of unique reflections	6495	3654
R[F^2^ > 2σ(F^2^)], wR(F^2^), S	0.052, 0.119, 0.861	0.050, 0.117, 1.041
No. of observed reflections with I > 2σ(I),	3395	2650
Δρ_max_, Δρ_min_ (eÅ^−3^)	+0.290, –0.062	+0.316, –0.069

**Table 5:** Genes and primer sequences used in the research.

Gene Symbol	Gene Name	Forward Sequence (3′→5′)	Reverse Sequence (3′→5′)
*ATM*	Ataxia telangiectasia mutated protein kinase	GCCGCGGTTGATACTACTTTG	GCAGCAGGGTGACAATAAACA
*ATR*	ATR serine/threonine kinase	AATGGTTGGAGAATGCTGGC	ACATCACCCTTGGACCAGAG
*BAX*	Bcl-2-like protein 4	TCAGGATGCGTCCACCAAGAAG	TGTGTCCACGGCGGCAATCATC
*BCL-2*	BCL-2 apoptosis regulator	ACTGAGTACCTGAACCGGCA	TACAGTTCCACAAAGGCATCCCAG
*SOD2*	Superoxide dismutase 2	CTTCAGGGTGGTATGGCTGT	TGGCCAGACCTTAATGTTCC
*CAT*	Catalase	GCTCCGCAATCCTACACCAT	GGACATCGGGTTTCTGAGGG
*GPX1*	Glutathione peroxidase 1	CAATCAGTTCGGACATCAGGAGA	TAAAGAGCGGGTGAGCCTTC
*TP53*	Tumor protein p53	CCTCAGCATCTTATCCGAGTGG	TGGATGGTGGTACAGTCAGAGC
*CDKN1A*	Cyclin dependent kinase inhibitor 1A/CDKN1A (p21)	CCTCATCCCGTGTTCTCCTTT	GTACCACCCAGCGGACAAGT
*CYCD*	D-type cyclins	CCGTCCATGCGGAAGATC	GAAGACCTCCTCCTCGCACT
*RNA18SN5*	18S ribosomal N5	GAAACTGCGAATGGCTCATTAAA	CACAGTTATCCAAGTGGGAGAGG
*BACT*	Beta-actin	AGAGCTACGAGCTGCCTGAC	AGCACTGTGTTGGCGTACAG

## Data Availability

Data will be made available on request.

## Supplementary Materials

The following supporting information can be downloaded at: www.mdpi.com/article/10.3390/molecules30071576/s1.
