# Supplementary material for: Anticancer Activity and Safety Profile of Novel 1-(4-Fluorophenoxyacetyl)-4-substituted Thio/Semicarbazide Derivatives
Source: Molecules. 2025 Mar 31;30(7):1576. doi: 10.3390/molecules30071576 (PMC11990103; doi:10.3390/molecules30071576)
Supplement: Supplementary file 1 [file molecules-30-01576-s001.zip › molecules-3520391-supplementary.pdf]

Anticancer Activity and Safety Profile of Novel 1-(4-Fluorophenoxyacetyl)-4-Substituted Thio/Semicarbazide Derivatives

Paweł Kozyra [1], Ewelina Humeniuk [2], Zbigniew Karczmarzyk [3], Adrian Borzęcki [1], Grzegorz Adamczuk [2], Agnieszka Korga-Plewko [2], Waldemar Wysocki [3] and Monika Pitucha [1,*]

[1] Independent Radiopharmacy Unit, Faculty of Pharmacy, Medical University of Lublin, 20-093 Lublin, Poland; adrian.borzecki@o2.pl (A.B.)
[2] Independent Medical Biology Unit, Faculty of Pharmacy, Medical University of Lublin, 20-093 Lublin, Poland; ewelinahumeniuk@umlub.pl (E.H.); grzegorz.adamczuk@umlub.pl (G.A.); agnieszka.korga-plewko@umlub.pl (A.K.-P.)
[3] Institute of Chemistry, University of Siedlce, 3 Maja 54, 08-110 Siedlce, Poland; zbigniew.karczmarzyk@uws.edu.pl (Z.K.); waldemar.wysocki@uws.edu.pl (W.W.)
* Correspondence: monika.pitucha@umlub.pl

Table S1. Selected torsion angles (°) for AB2 and AB5.

Torsion Angle	AB2	AB5
C22-C21-N1-C2	142.1(3)	137.9(3)
C21-N1-C2-N4	178.9(3)	-175.2(3)
N1-C2-N4-N5	-177.5(3)	-177.0(3)
C2-N4-N5-C6	163.9(3)	167.4(3)
N4-N5-C6-C8	-177.8(3)	-178.1(3)
N5-C6-C8-O9	11.2(4)	9.2(4)
C6-C8-O9-C31	-178.2(3)	-179.1(2)
C8-O9-C31-C32	177.2(2)	176.3(2)
C21-N1-C2-S3	-2.0(5)	3.8(5)
N4-N5-C6-O7	3.8(5)	2.1(4)

Table S2. Hydrogen-bond geometry (Å, °).

**D-H…A**	**D-H**	**H…A**	**D…A**	**D-H…A**
AB2				
N1-C1…Cl22	0.82(4)	2.59(4)	2.964(3)	109(3)
N4-H4…O7	0.90(3)	2.40(3)	2.719(3)	101(2)
N5-H5…S3	0.94(3)	2.38(3)	2.919(3)	116(2)
N1-H1…O7$^{(i)}$	0.82(4)	2.21(4)	2.934(3)	148(4)
N4-H4…O7$^{(i)}$	0.90(3)	2.02(4)	2.834(3)	151(3)
(*i*) = -*x*,1-*y*,-*z*				
AB5				
N1-C1…Cl22	0.82(4)	2.62(4)	2.958(3)	107(3)
N4-H4…O7	0.84(4)	2.42(4)	2.704(3)	101(3)
N5-H5…S3	0.88(3)	2.45(3)	2.903(3)	112(3)
N5-H5…O9	0.88(3)	2.20(3)	2.563(3)	104(2)
N1-H1…O7$^{(i)}$	0.82(4)	2.18(4)	2.932(3)	152(4)
N4-H4…O7$^{(i)}$	0.84(4)	2.01(4)	2.797(3)	155(3)
(*i*) = -*x*,1-*y*,-*z*				

SYNTHESIS

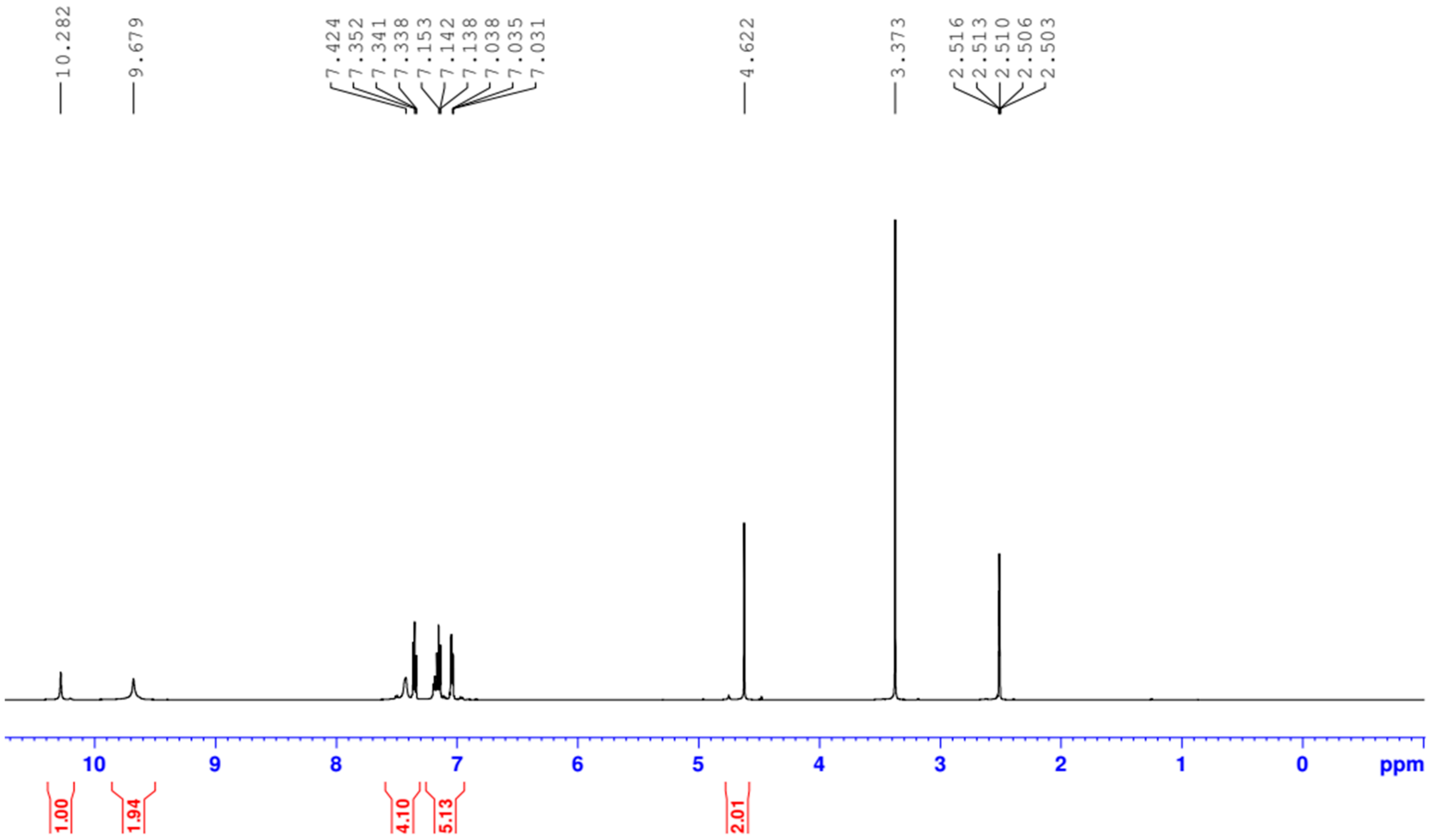

Figure S1: ^{1}H-NMR spectrum of compound **AB1**.

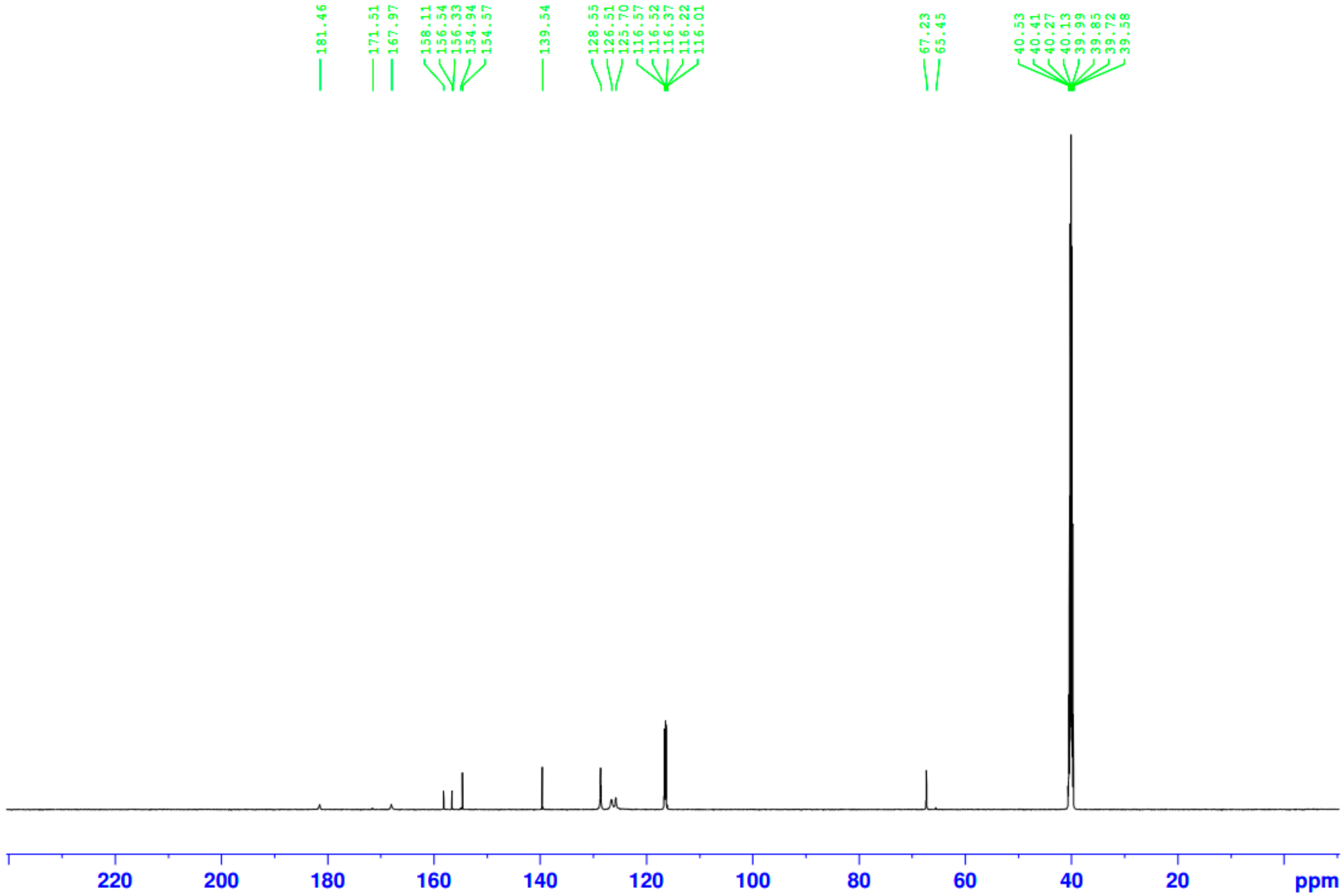

Figure S2: ^{13}C-NMR spectrum of compound **AB1**.

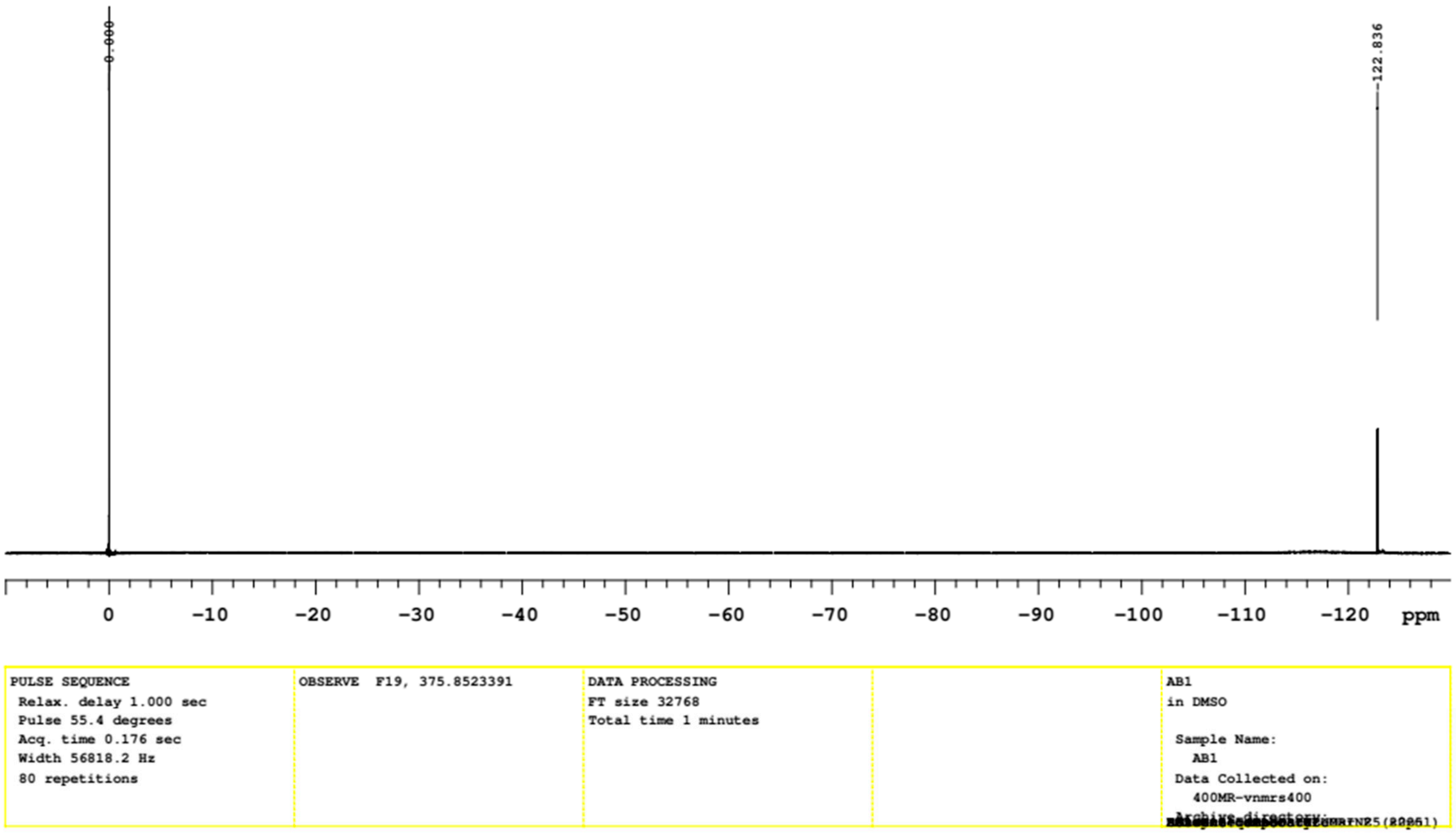

Figure S3: ^{19}F-NMR spectrum of compound **AB1.**

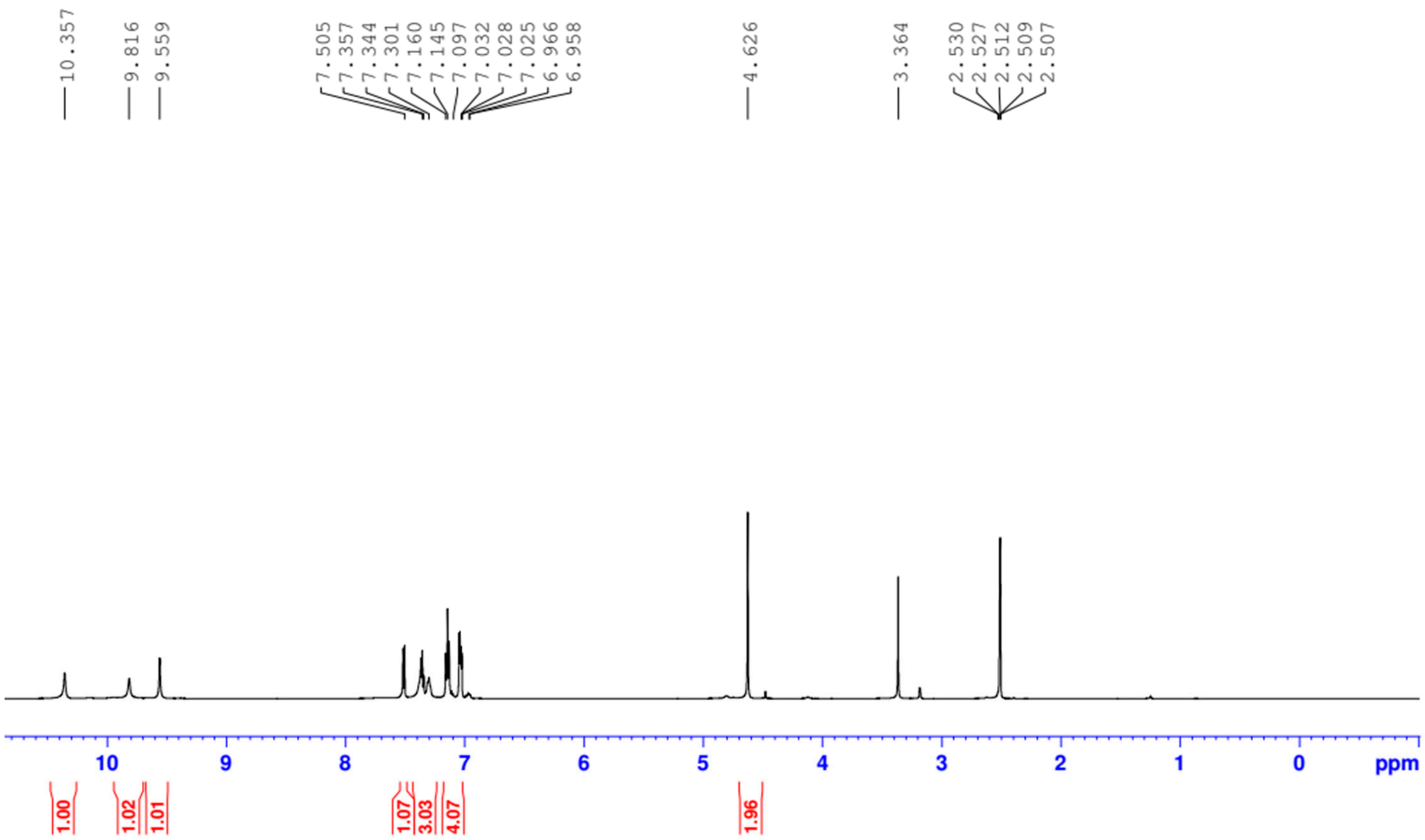

Figure S4: ^{1}H-NMR spectrum of compound **AB2**.

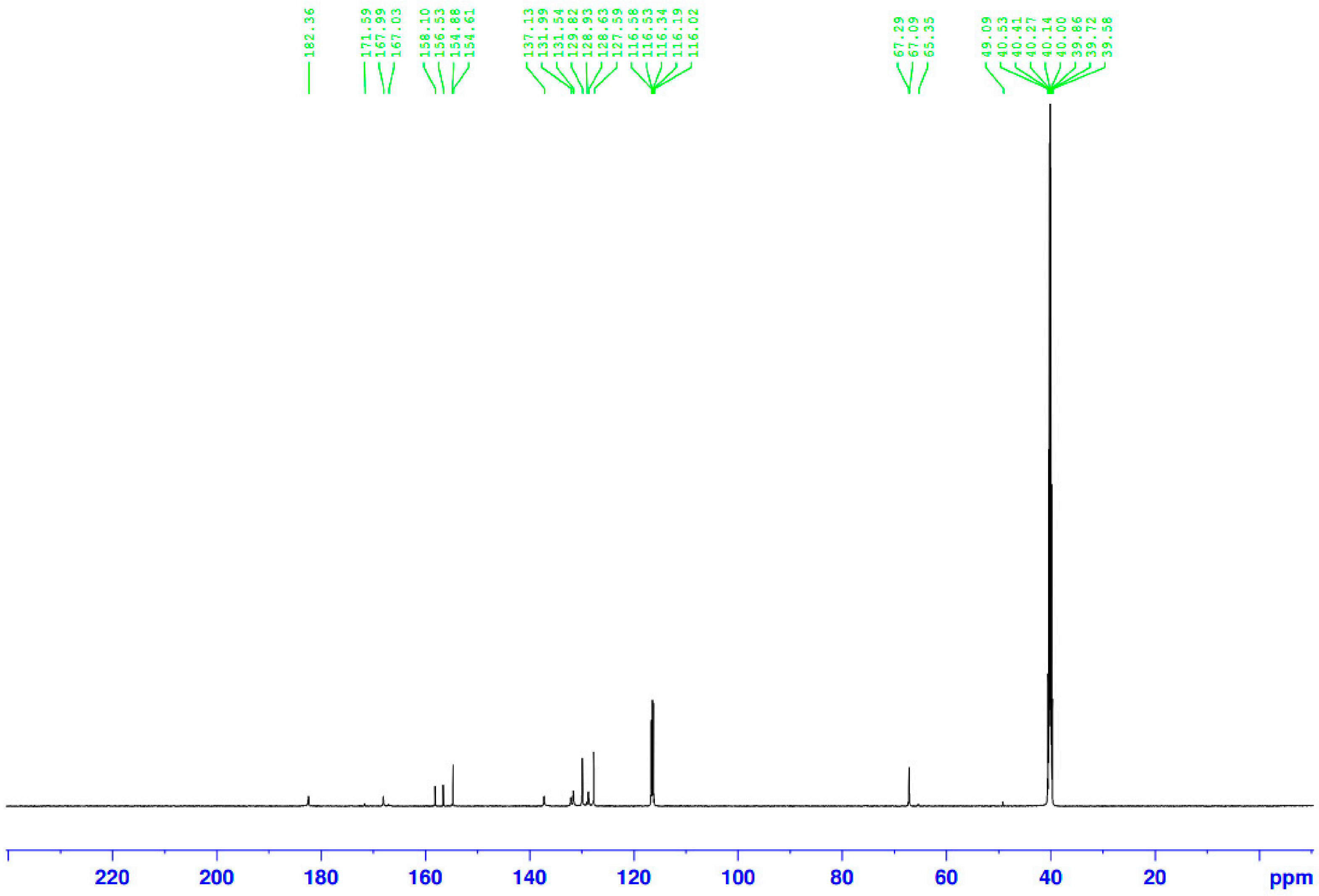

Figure S5: ^{13}C-NMR spectrum of compound **AB2**.

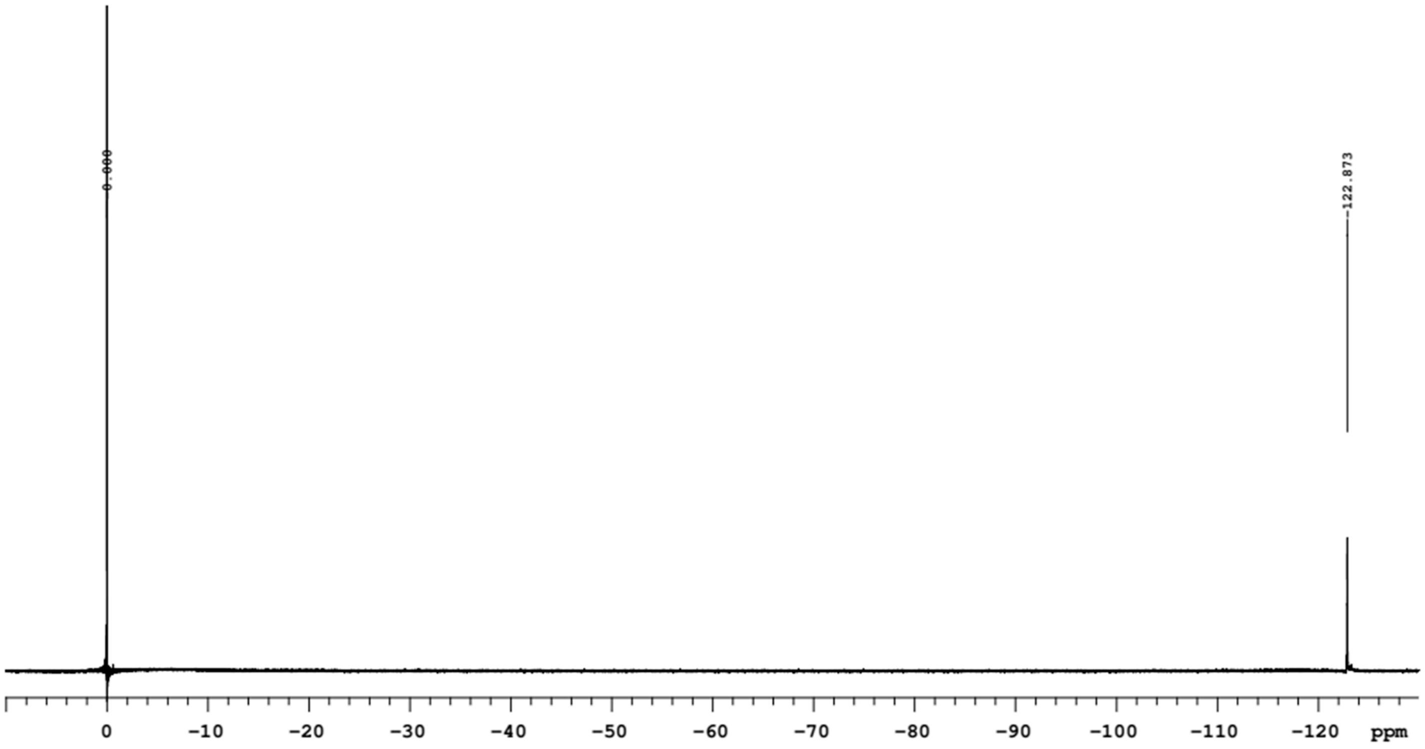

PULSE SEQUENCE	OBSERVE F19, 375.8523391	DATA PROCESSING		AB2
Relax. delay 1.000 sec<br>Pulse 55.4 degrees<br>Acq. time 0.176 sec<br>Width 56818.2 Hz<br>96 repetitions		FT size 32768<br>Total time 1 minutes		in DMSO<br><br>Sample Name:<br>AB2<br>Data Collected on:<br>400MR-vnmrs400<br>[illegible]

Figure S6: ^{19}F-NMR spectrum of compound **AB2.**

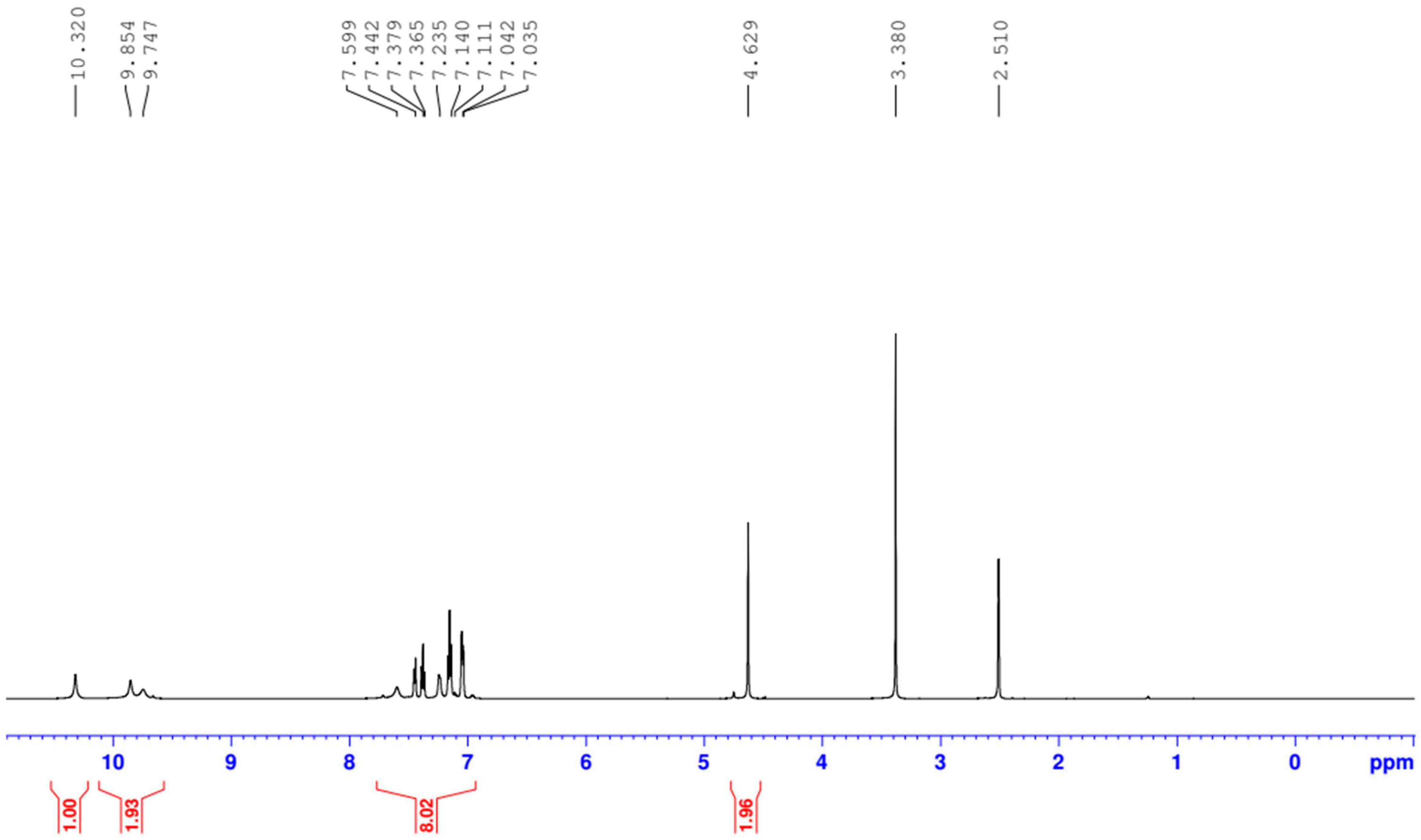

Figure S7: ^{1}H-NMR spectrum of compound **AB3**.

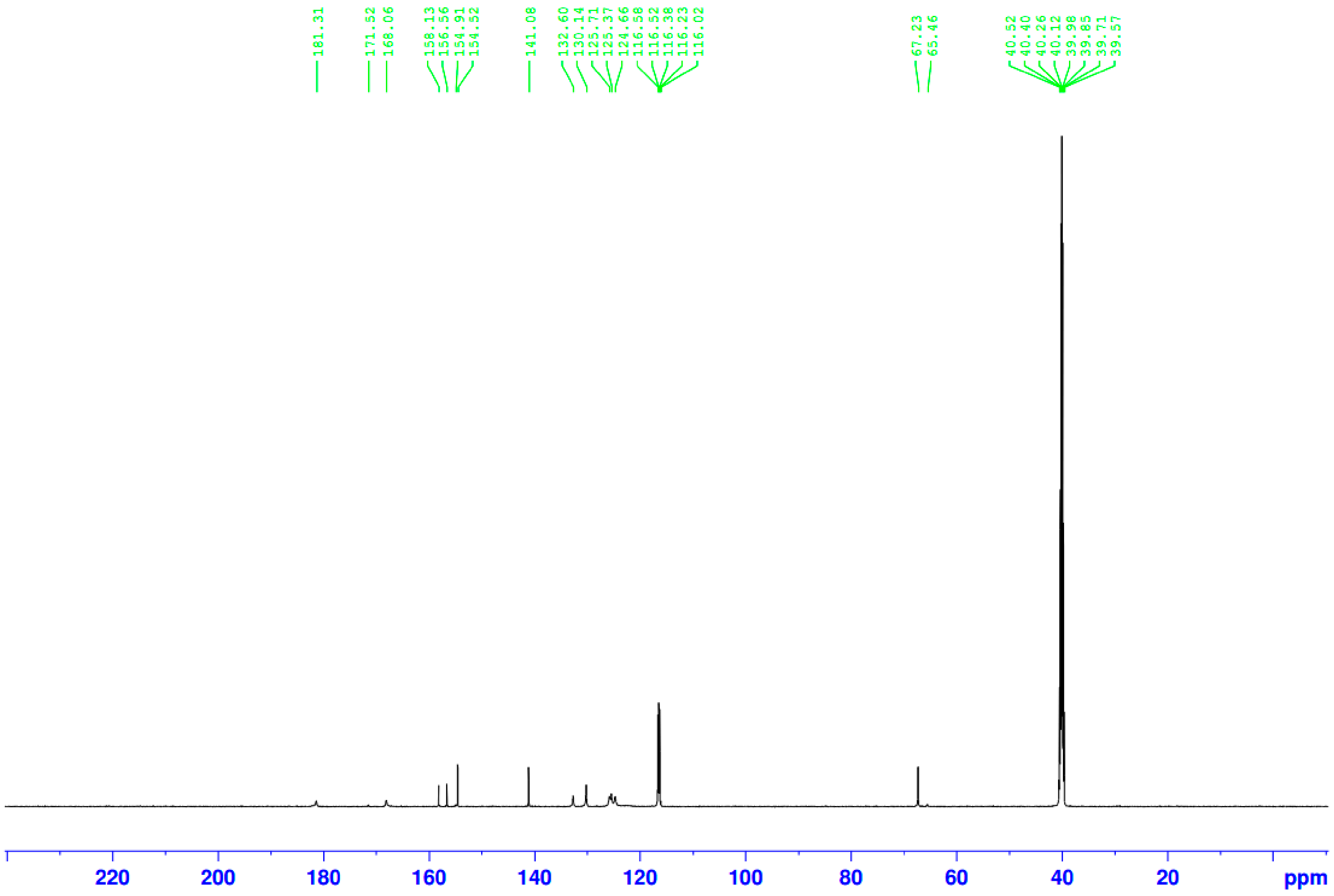

Figure S8: ^{13}C-NMR spectrum of compound **AB3**.

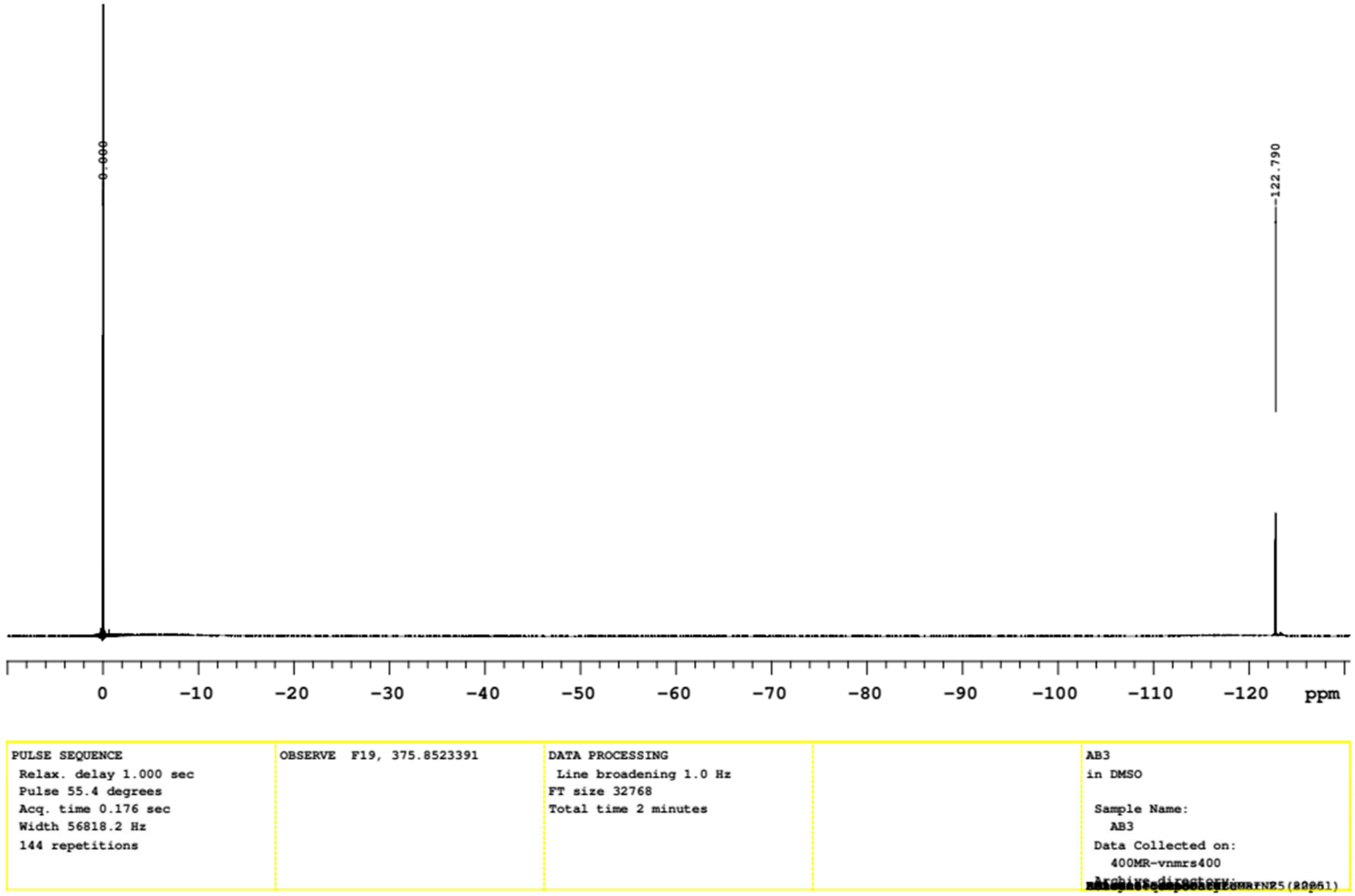

Figure S9: ^{19}F-NMR spectrum of compound **AB3.**

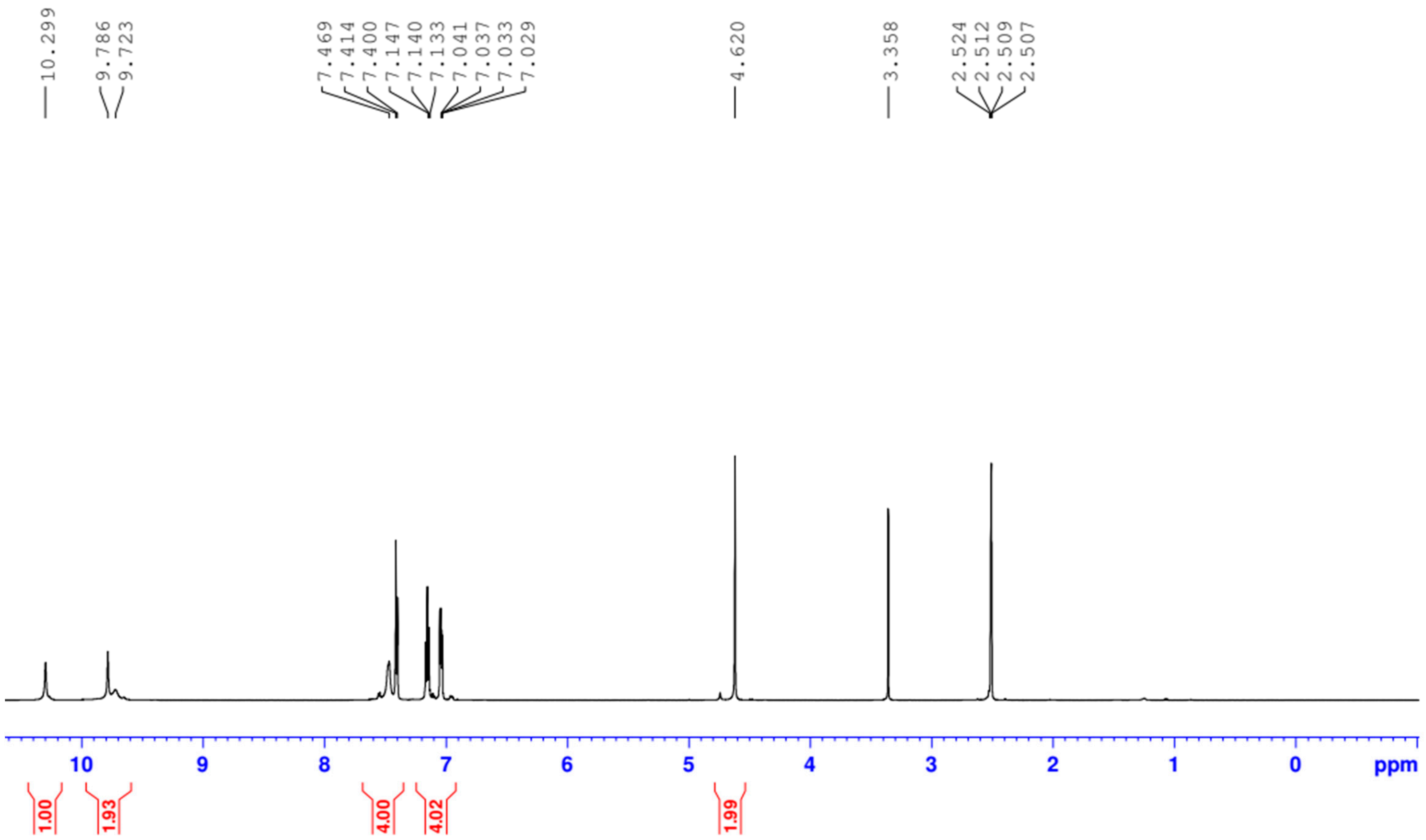

Figure S10: ^{1}H-NMR spectrum of compound **AB4**.

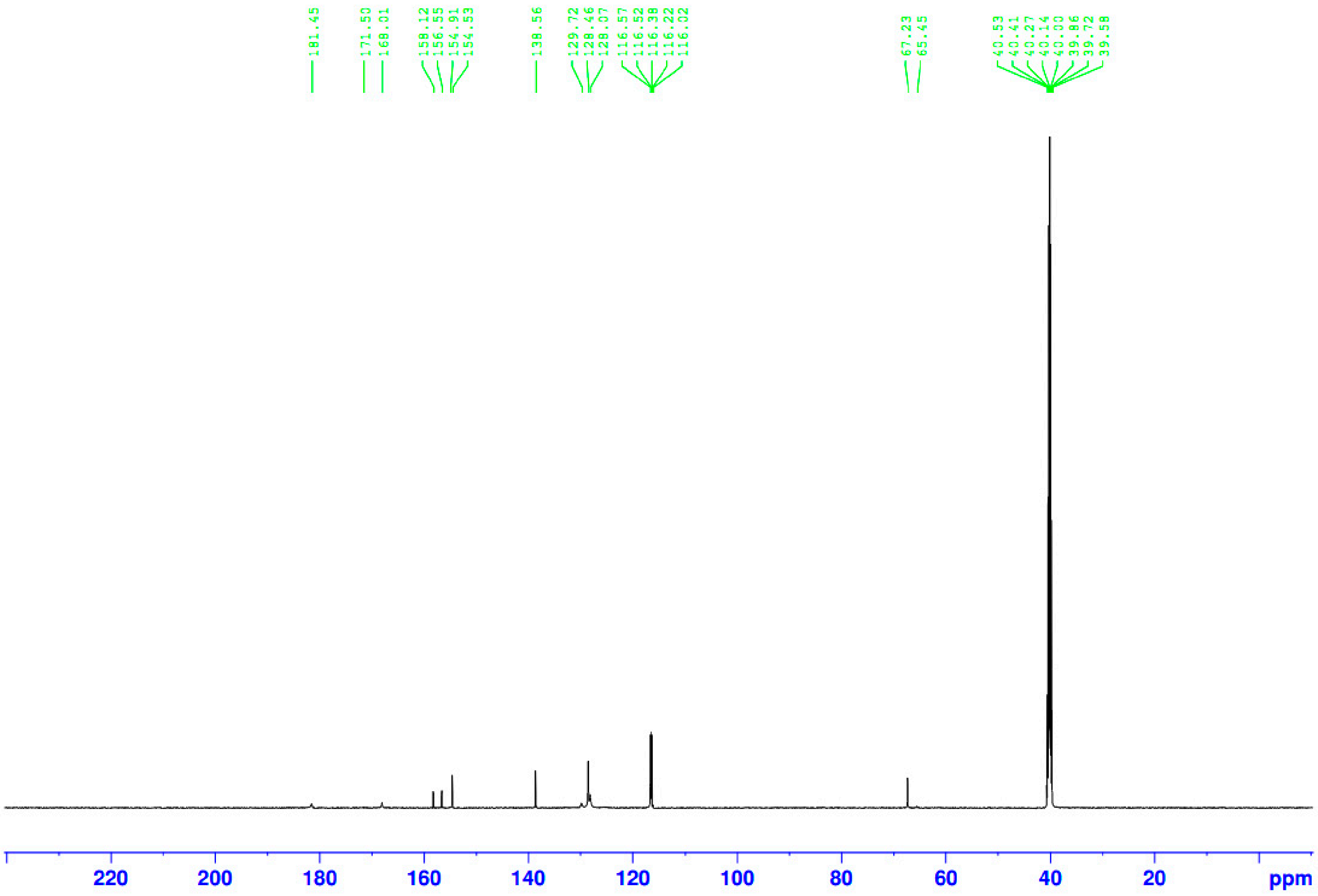

Figure S11: ^{13}C-NMR spectrum of compound **AB4**.

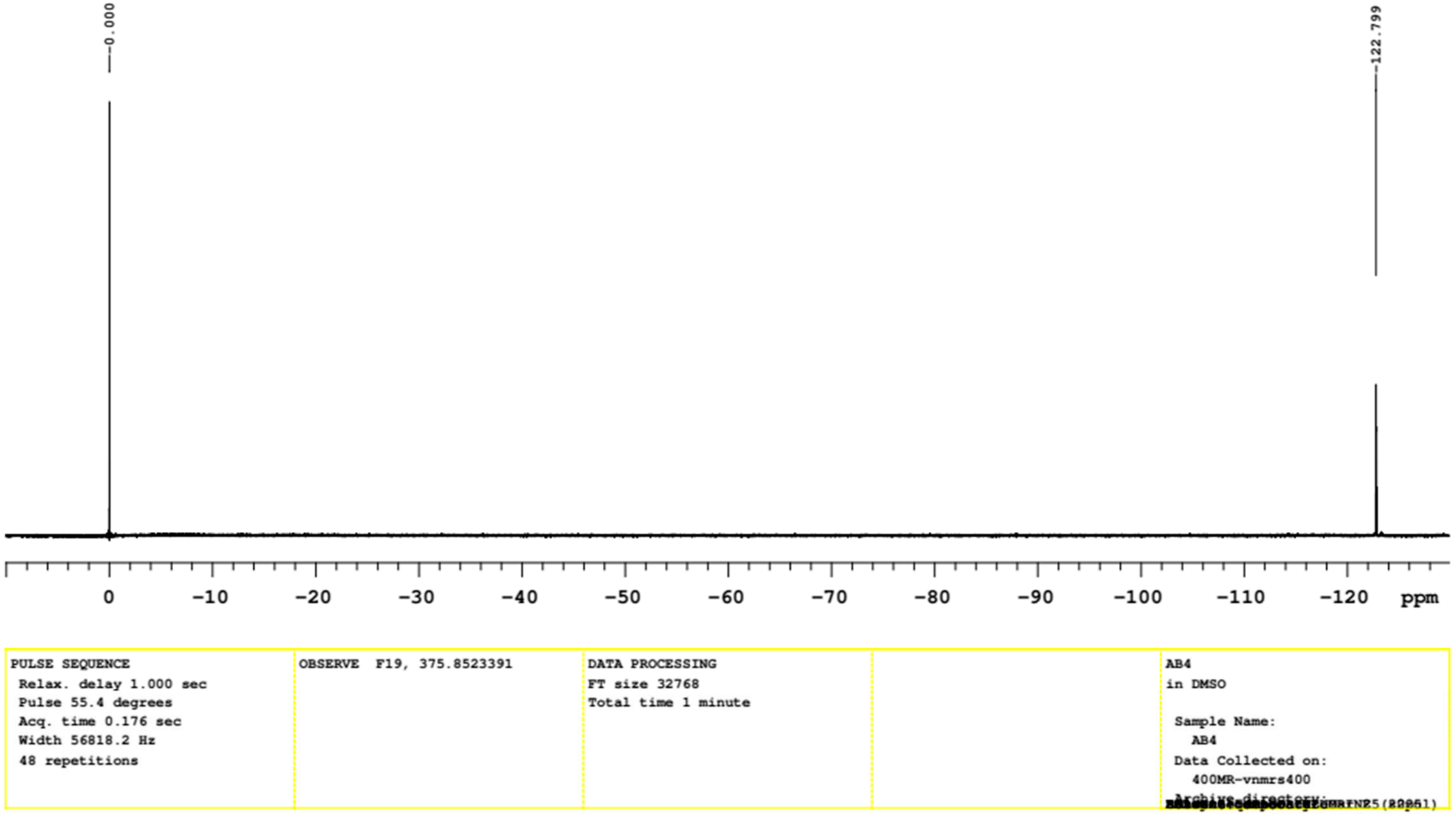

Figure S12: ^{19}F-NMR spectrum of compound **AB4.**

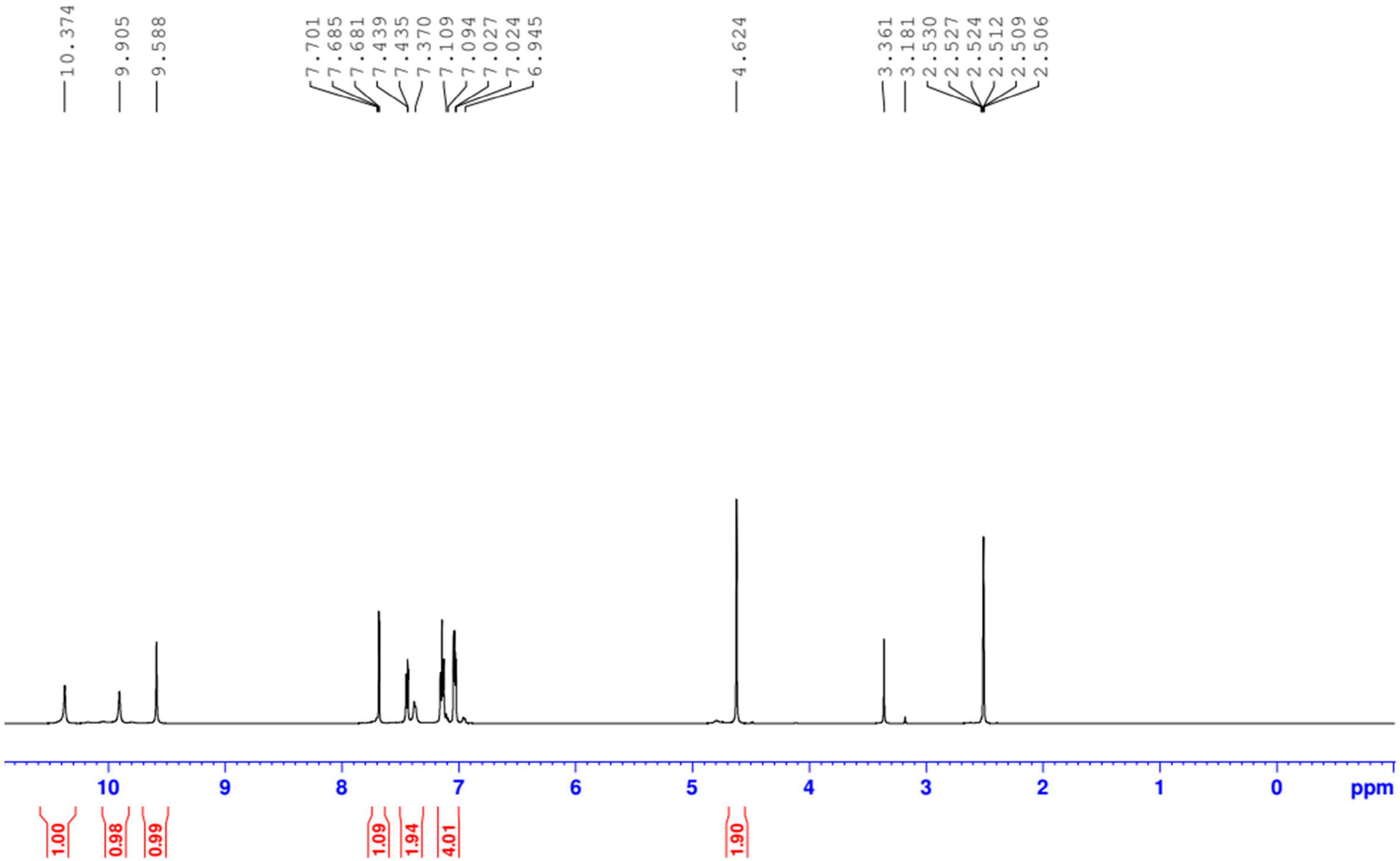

Figure S13: ^{1}H-NMR spectrum of compound **AB5**.

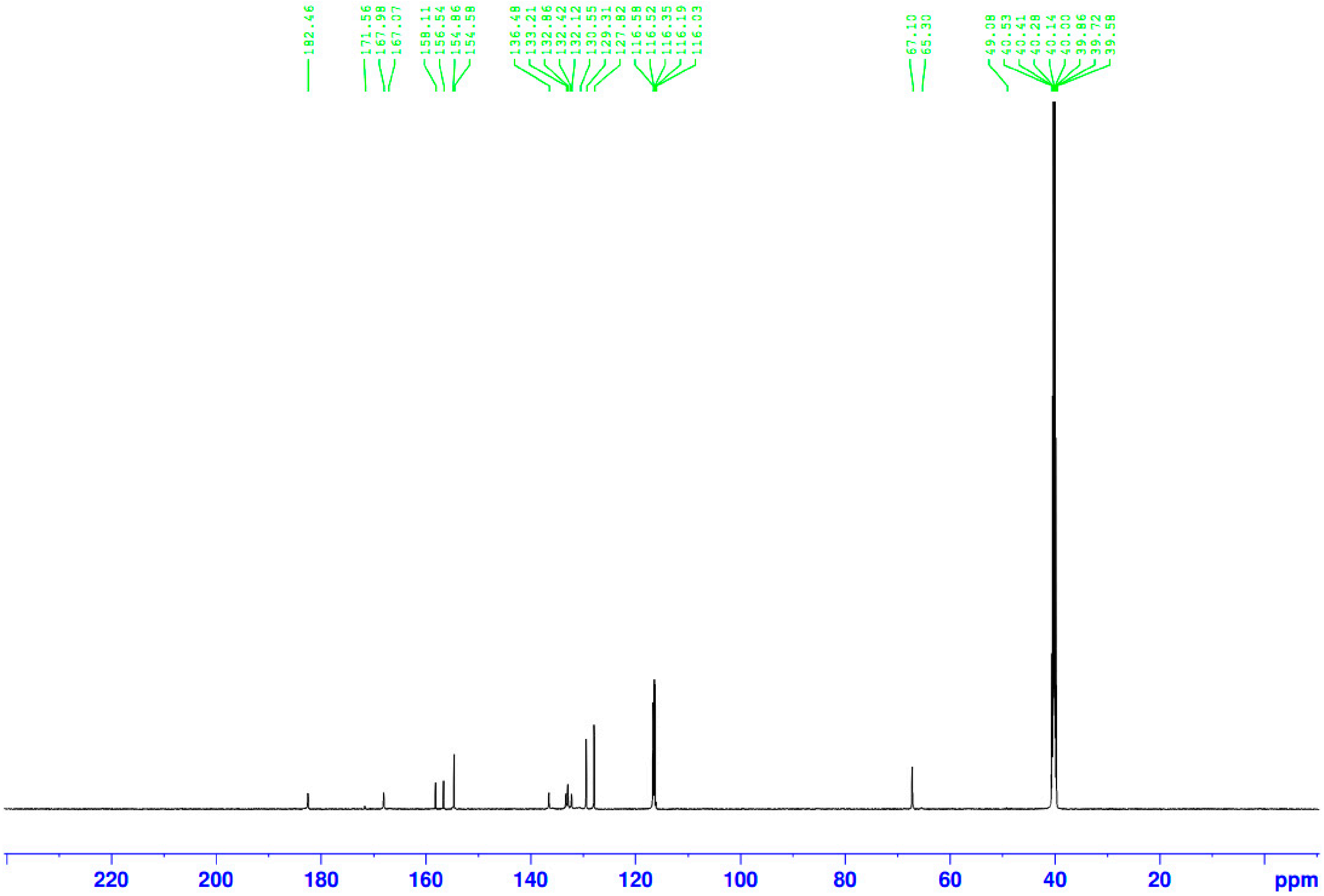

Figure S14: ^{13}C-NMR spectrum of compound **AB5**.

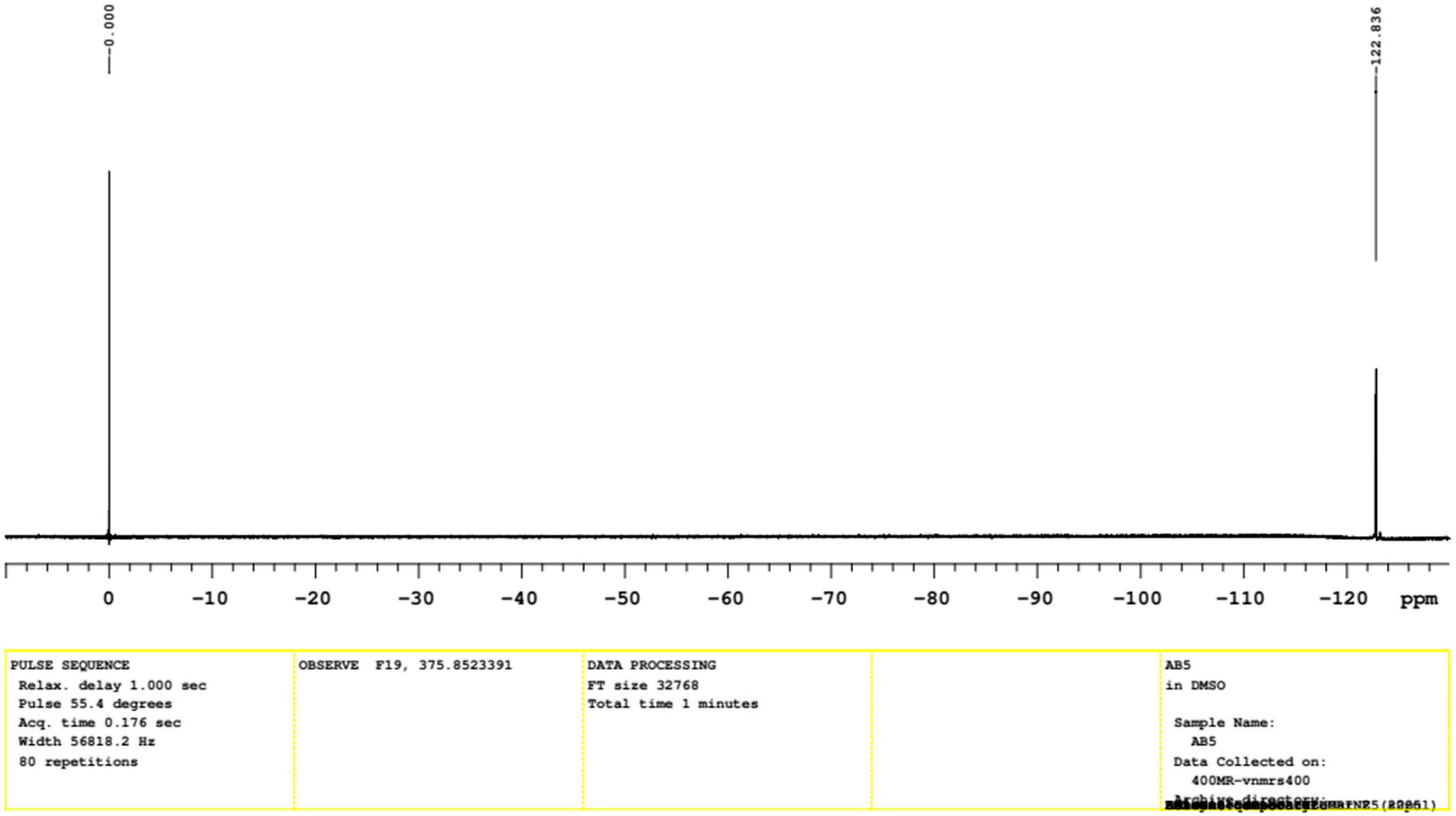

Figure S15: ^{19}F-NMR spectrum of compound **AB5.**

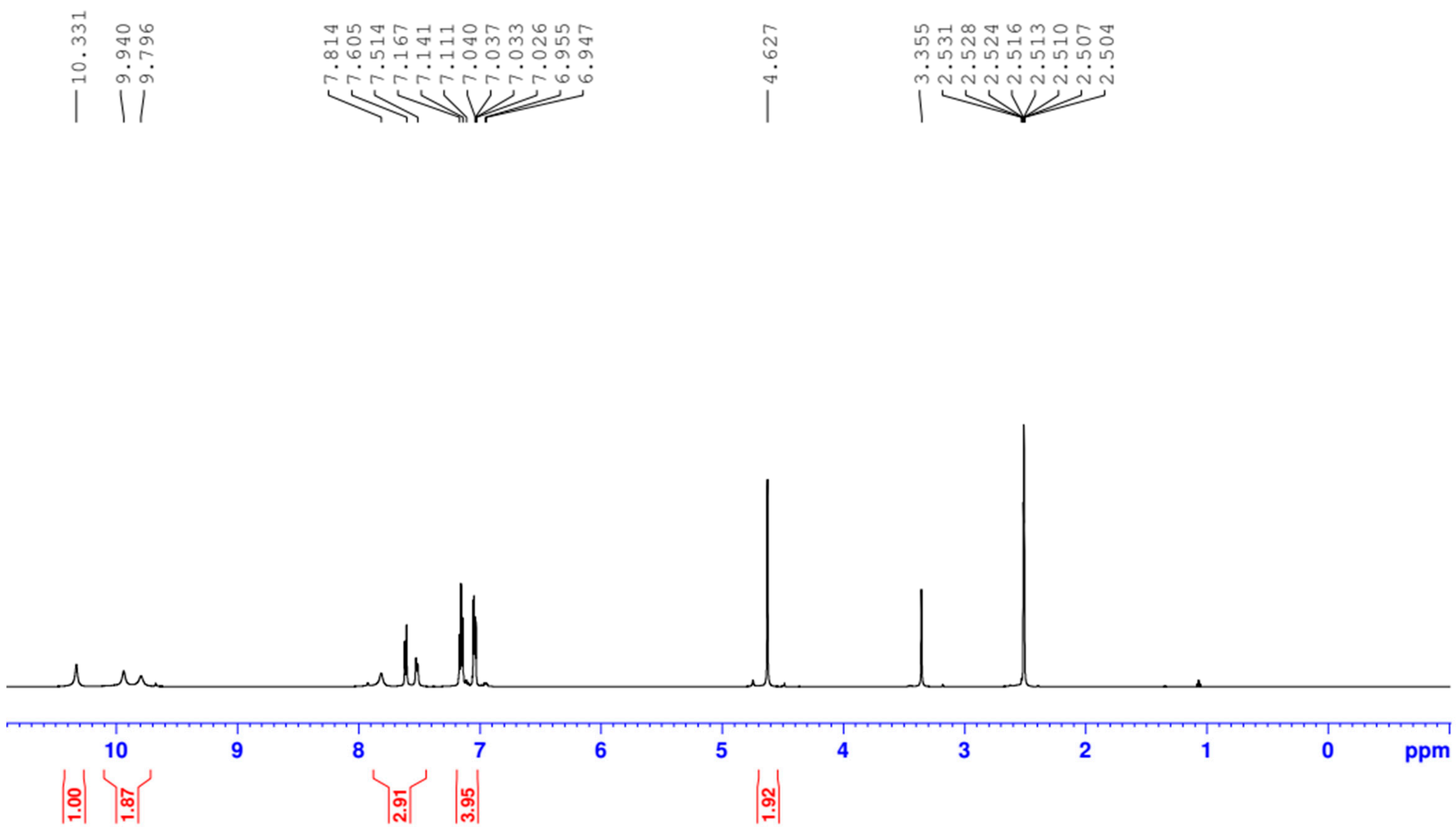

Figure S16: ^{1}H-NMR spectrum of compound **AB6**.

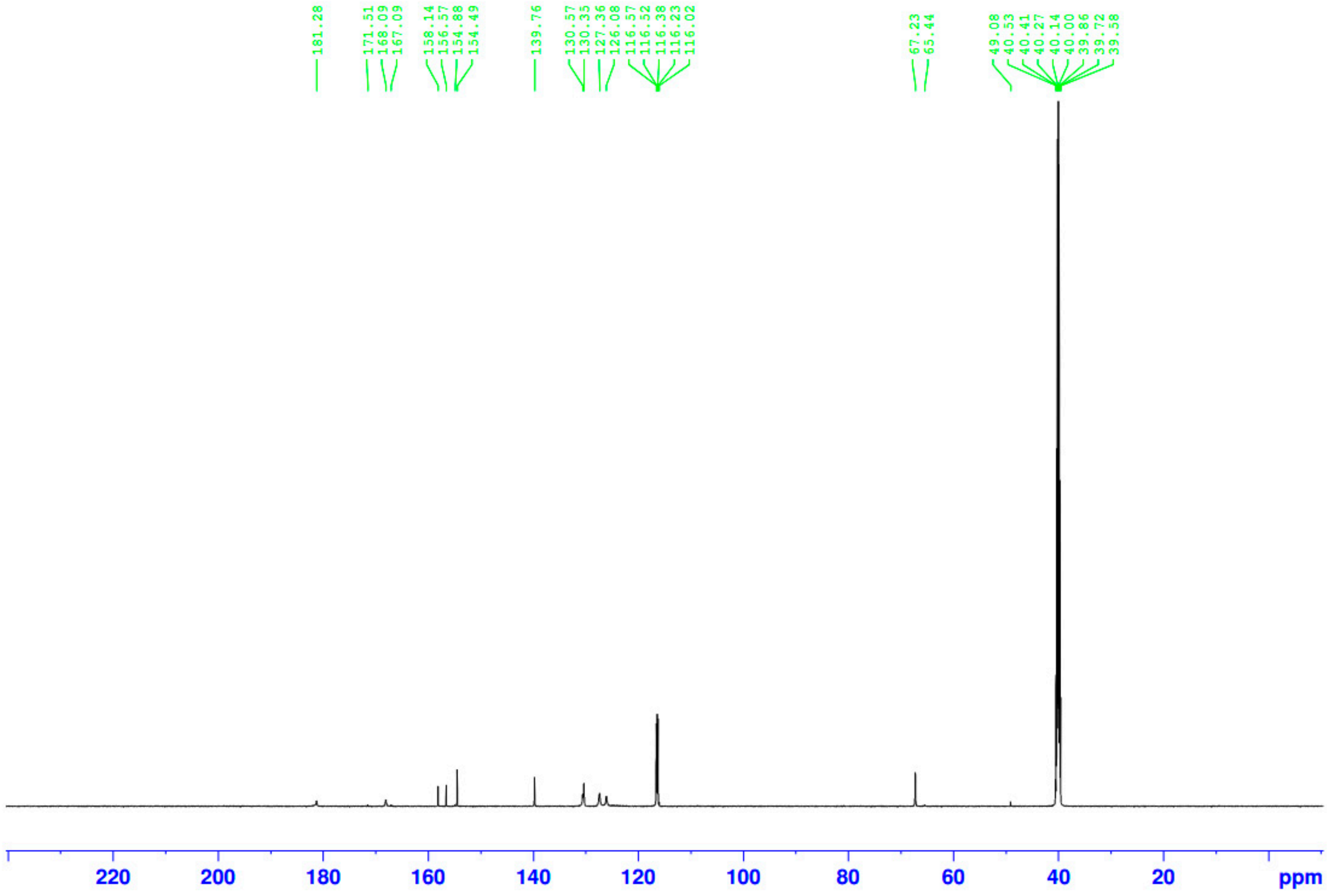

Figure S17: ^{13}C-NMR spectrum of compound **AB6**.

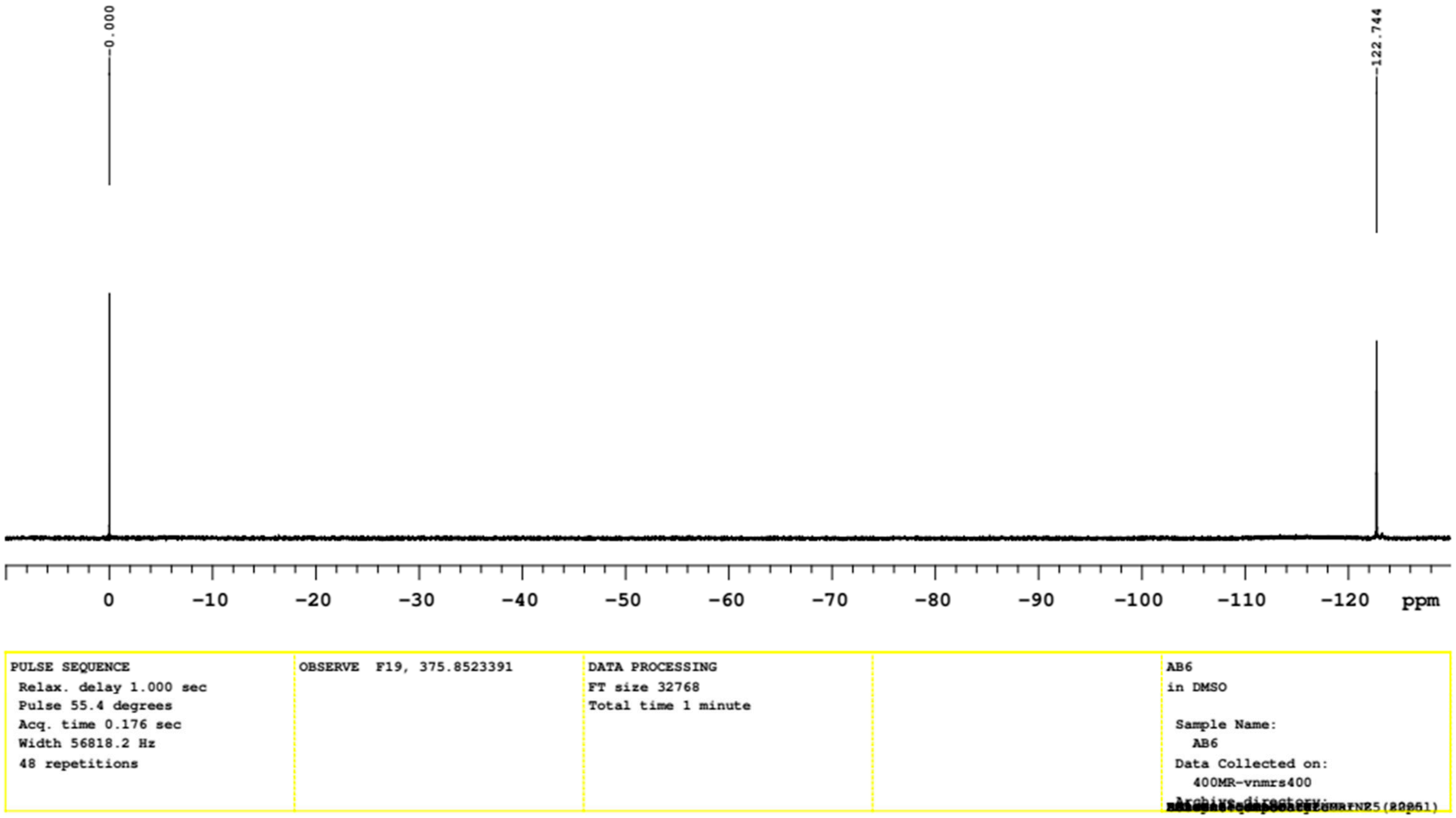

Figure S18: ^{19}F-NMR spectrum of compound **AB6.**

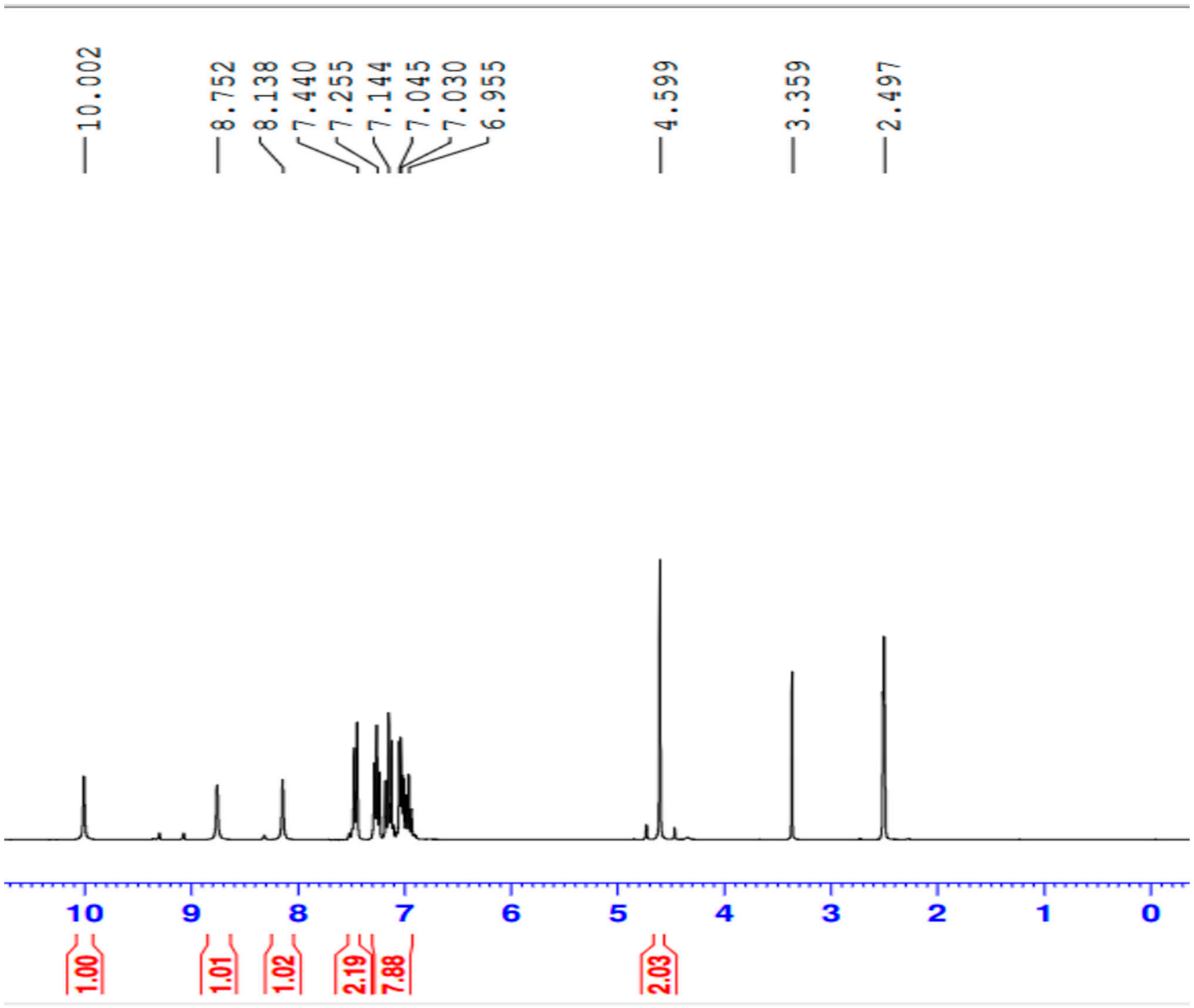

Figure S19: ^{1}H-NMR spectrum of compound **AB7**.

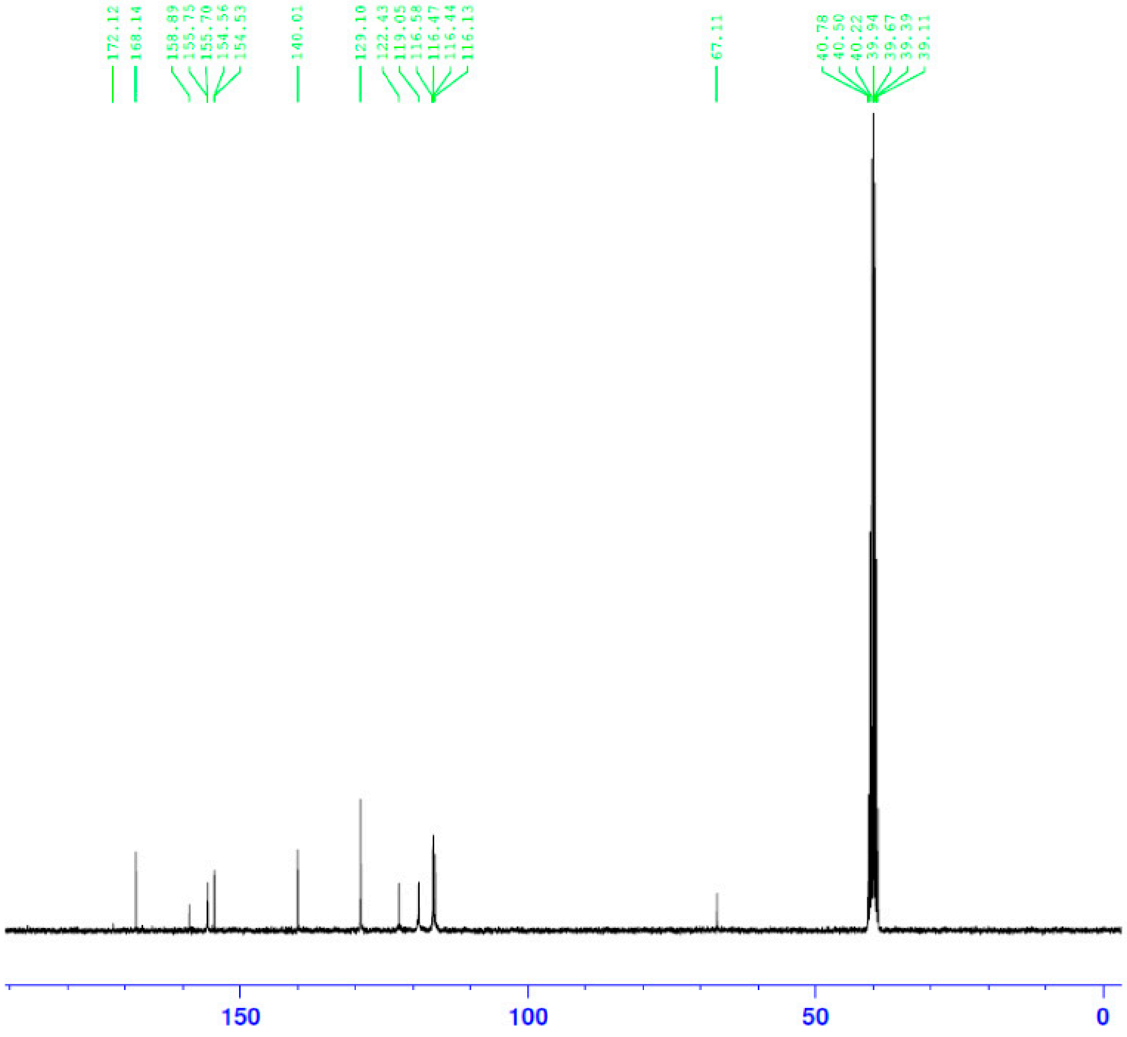

Figure S20: ^{13}C-NMR spectrum of compound **AB7**.

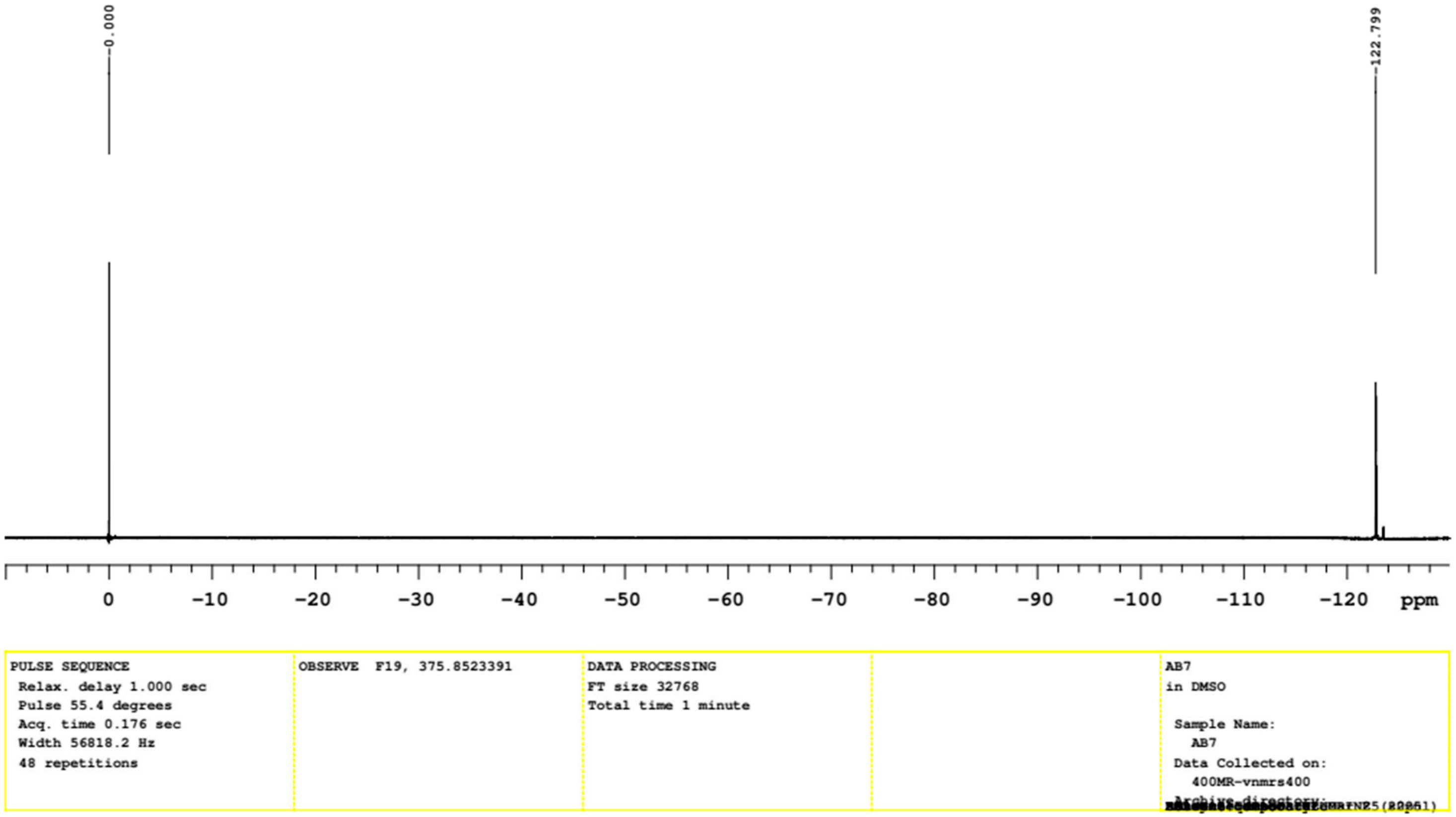

Figure S21: ^{19}F-NMR spectrum of compound **AB7.**

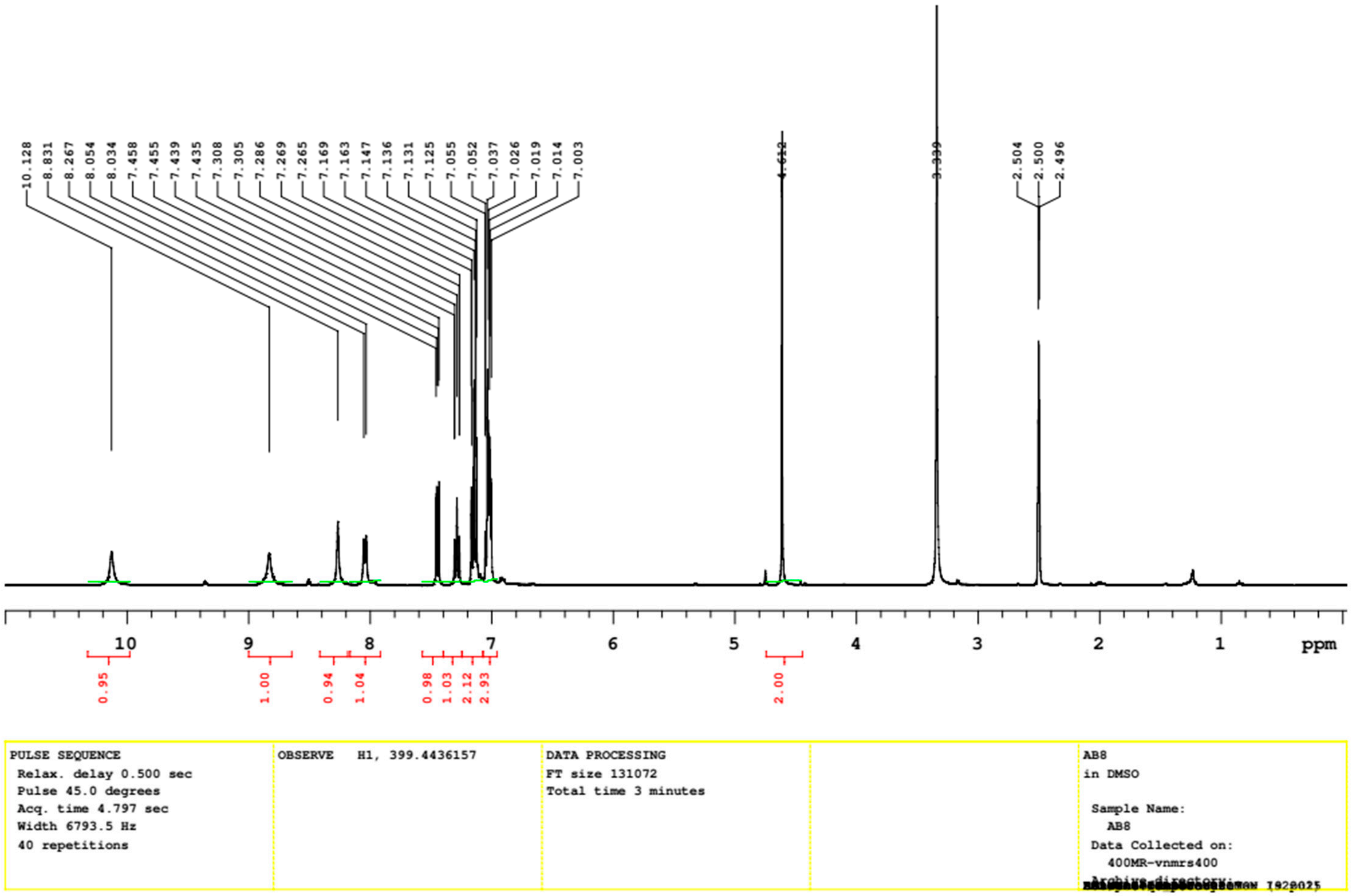

Figure S22: ^{1}H-NMR spectrum of compound **AB8**.

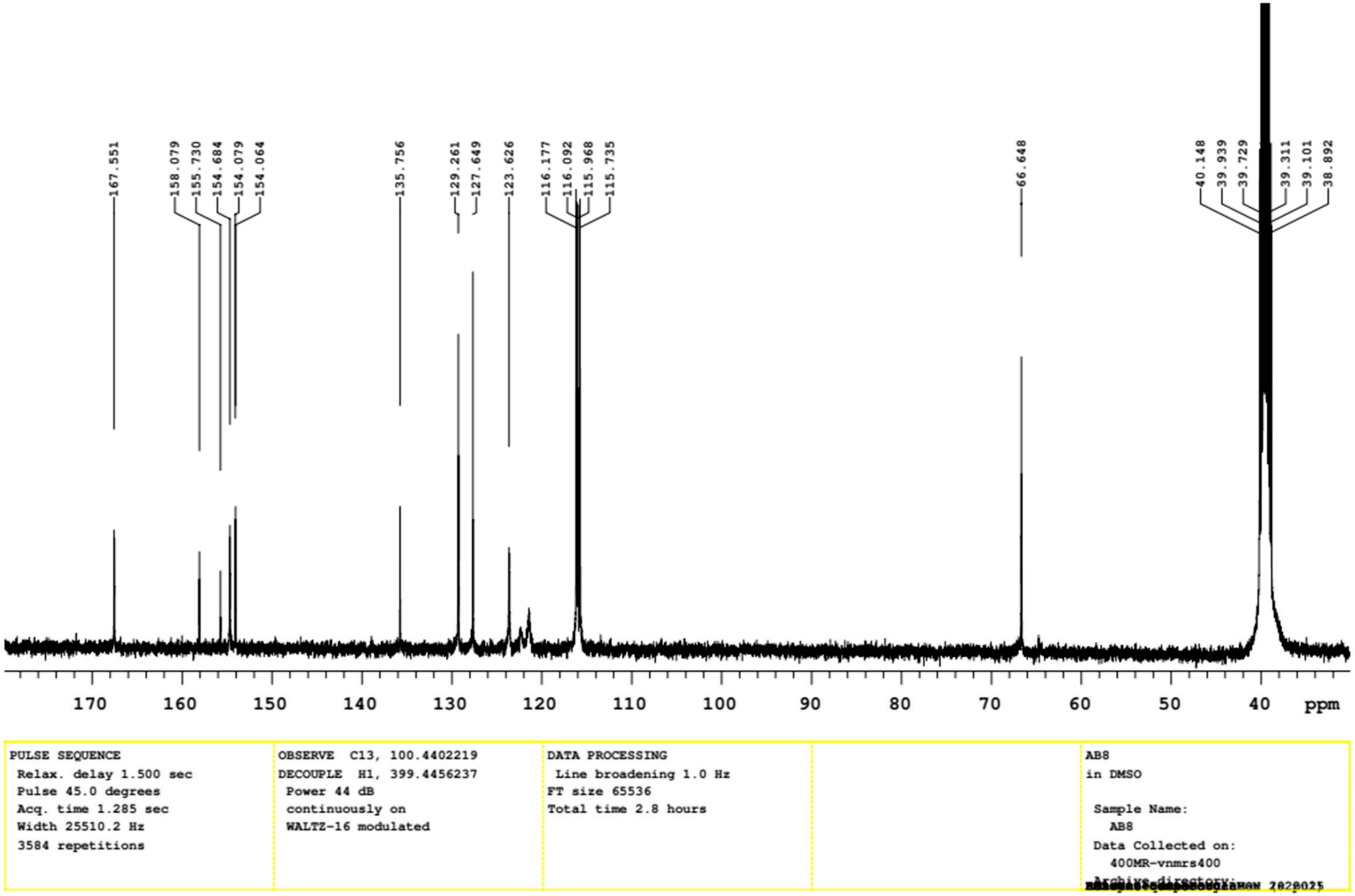

Figure S23: ^{13}C-NMR spectrum of compound **AB8**.

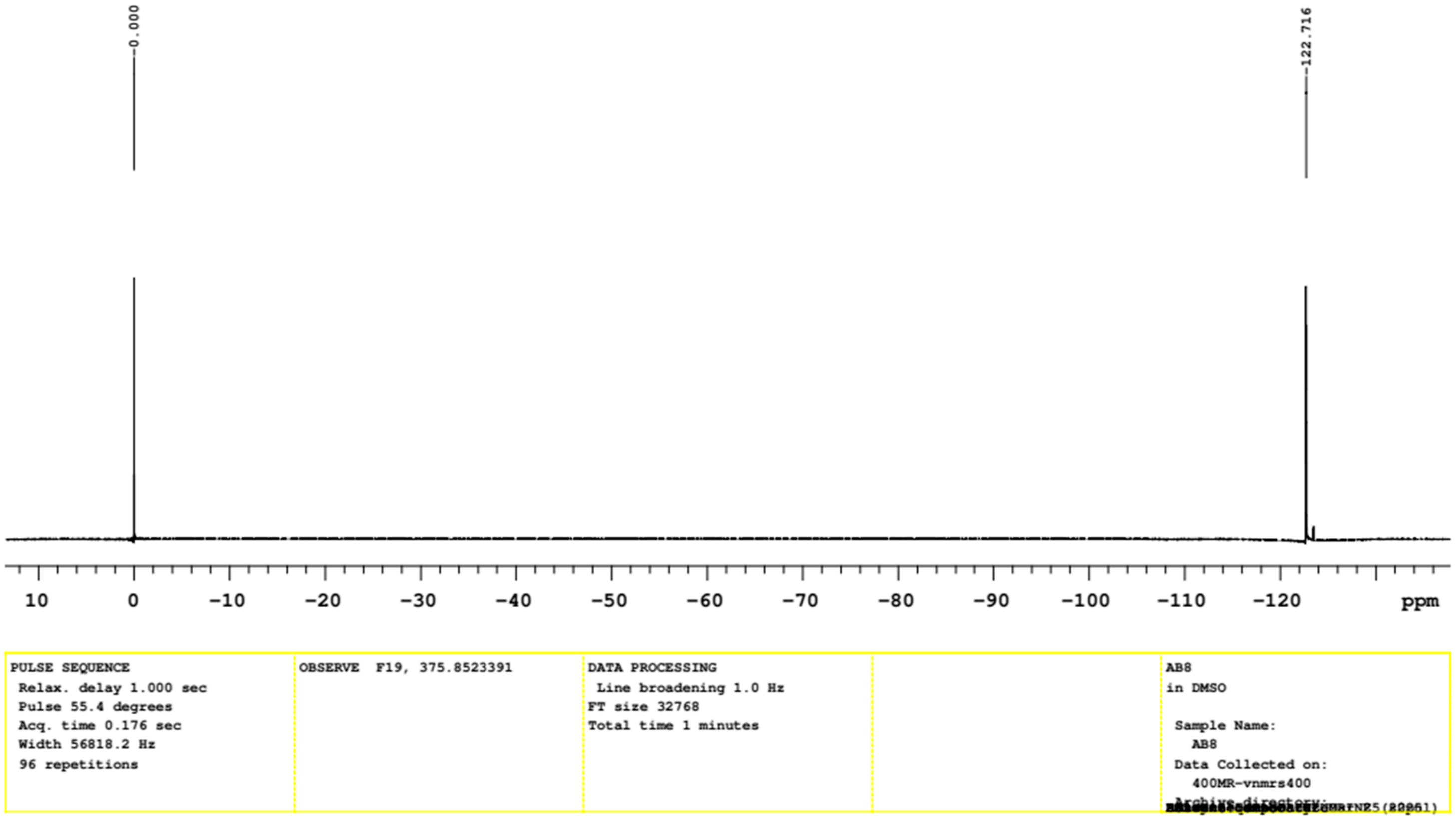

Figure S24: ^{19}F-NMR spectrum of compound **AB8.**

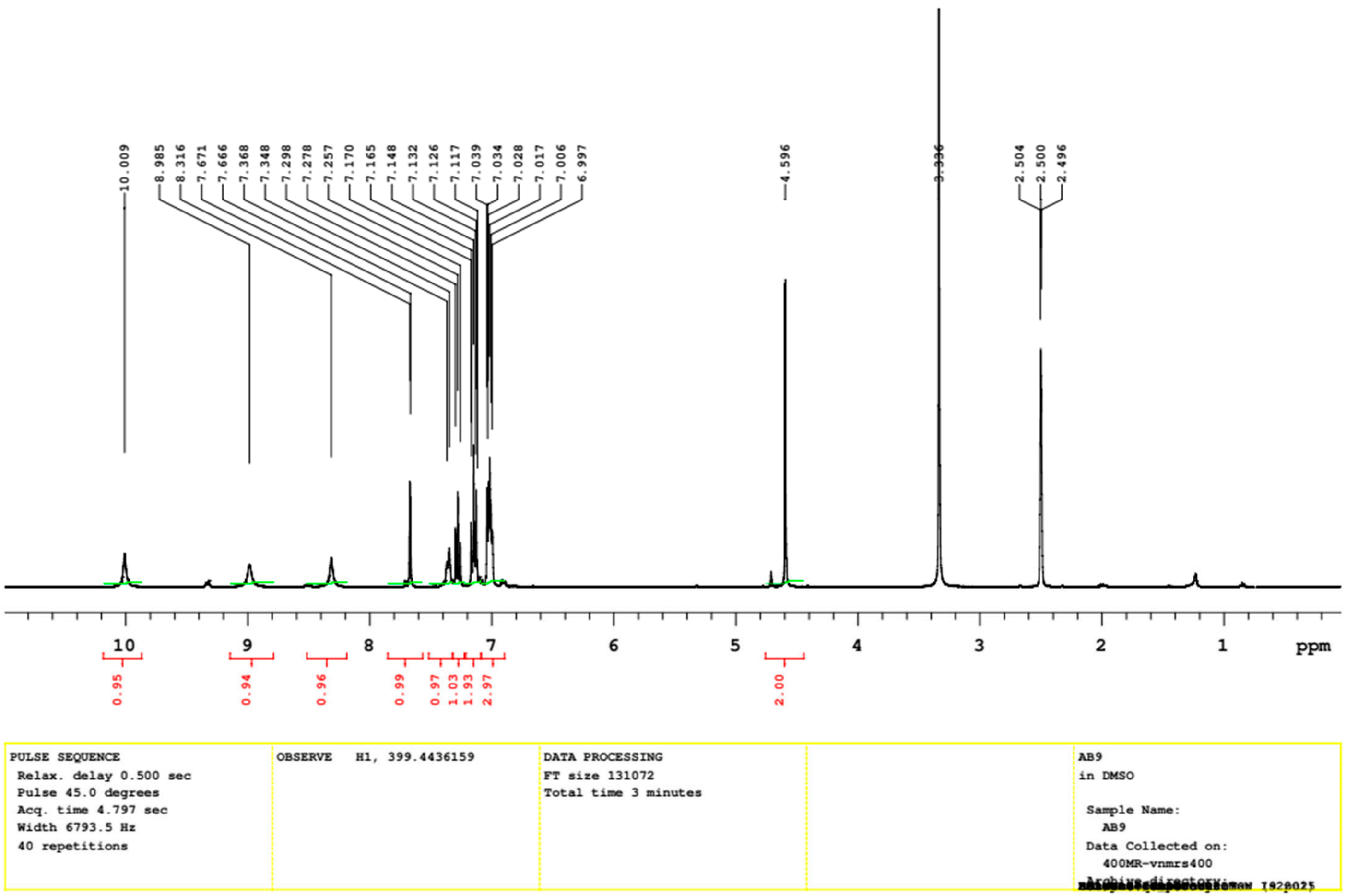

Figure S25: ^{1}H-NMR spectrum of compound **AB9**.

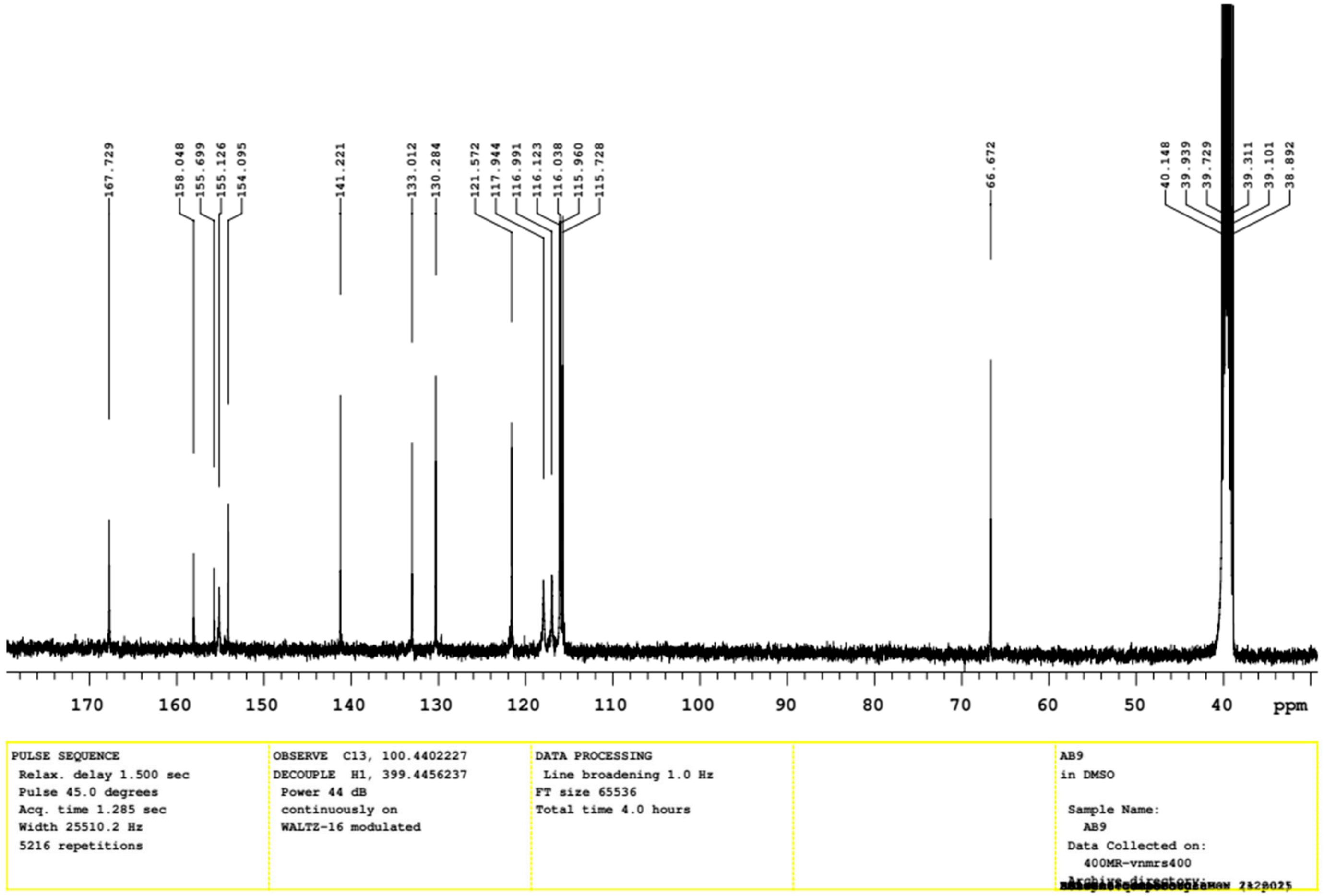

Figure S26: ^{13}C-NMR spectrum of compound **AB9**.

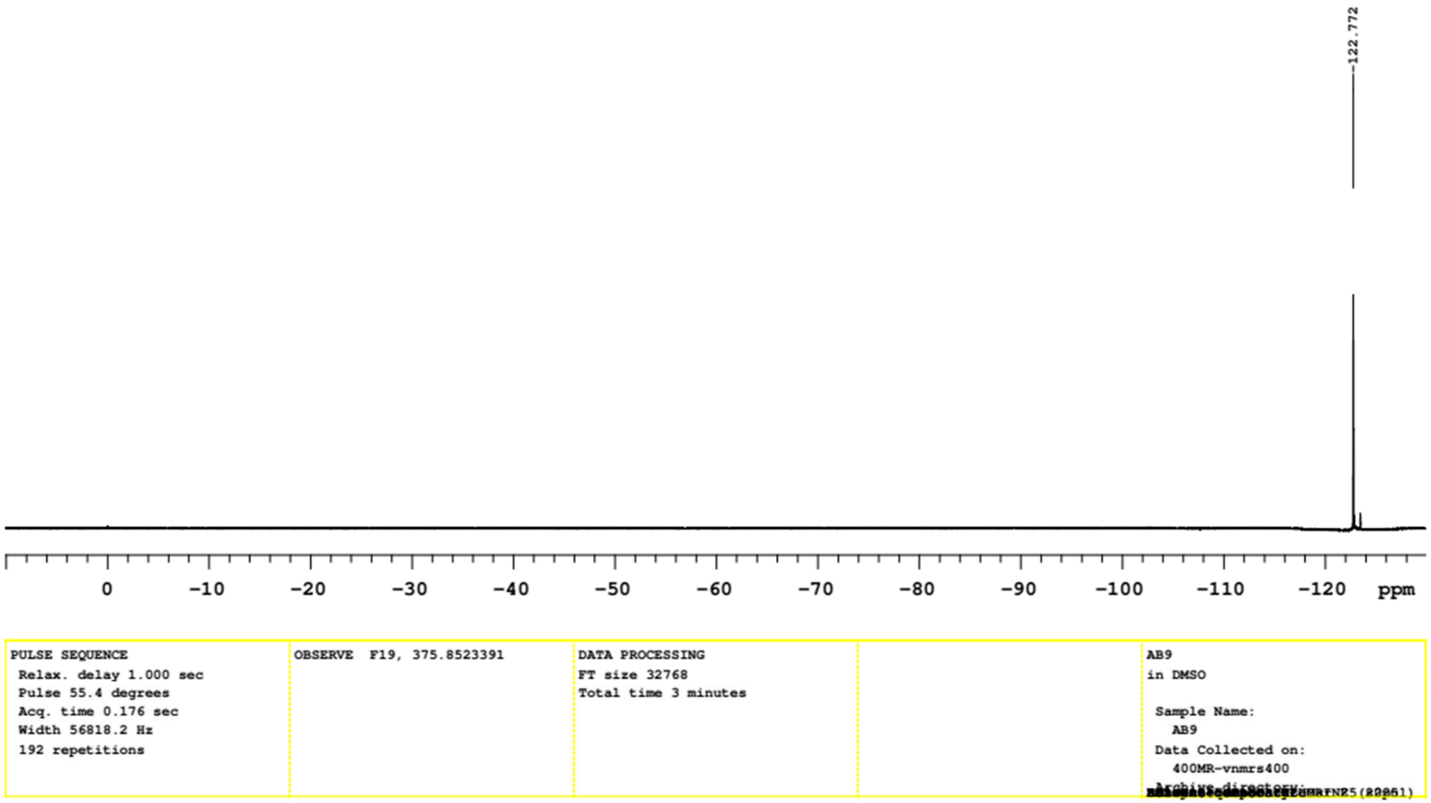

Figure S27: ^{19}F-NMR spectrum of compound **AB9.**

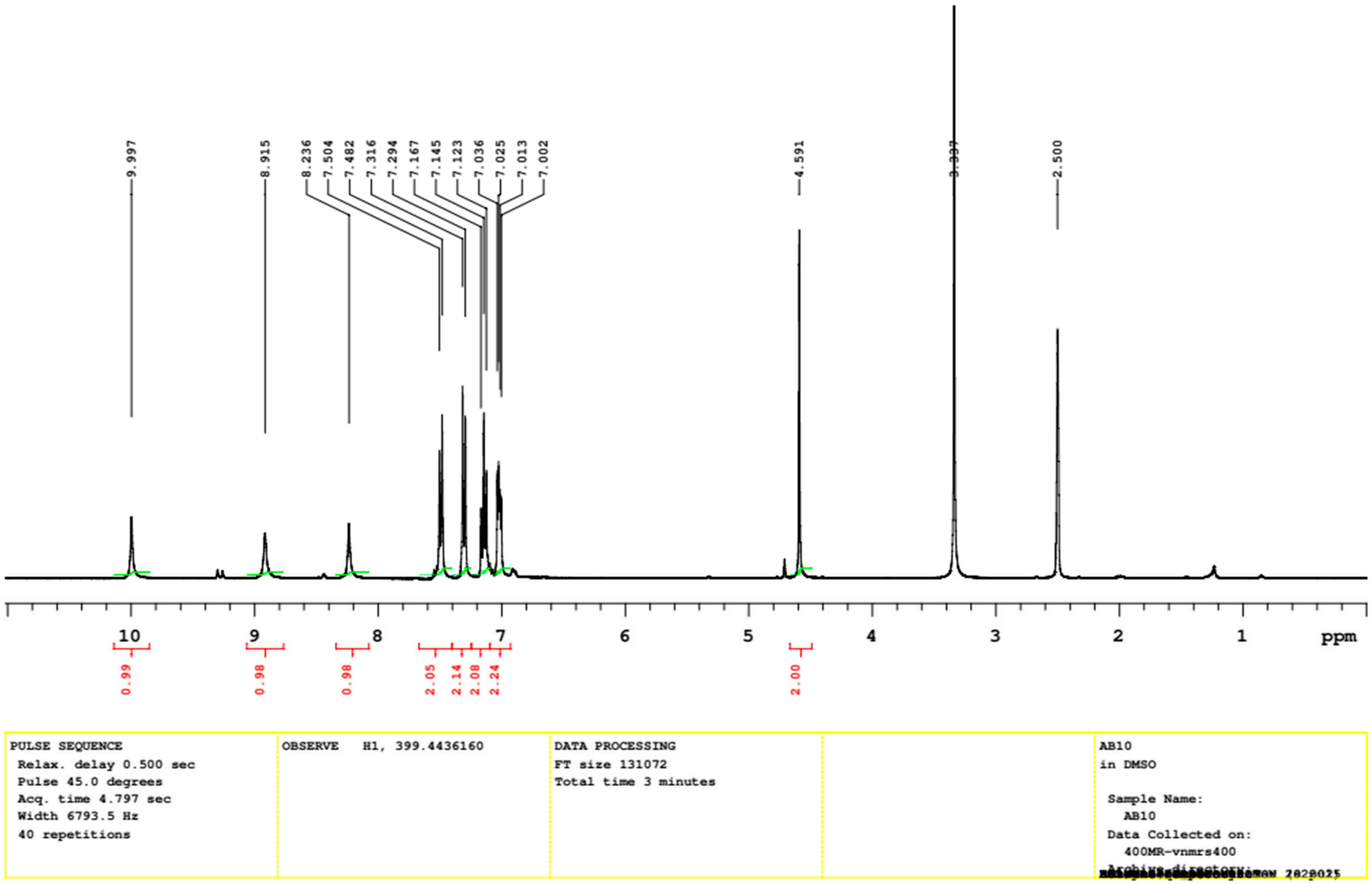

Figure S28: ^{1}H-NMR spectrum of compound **AB10**.

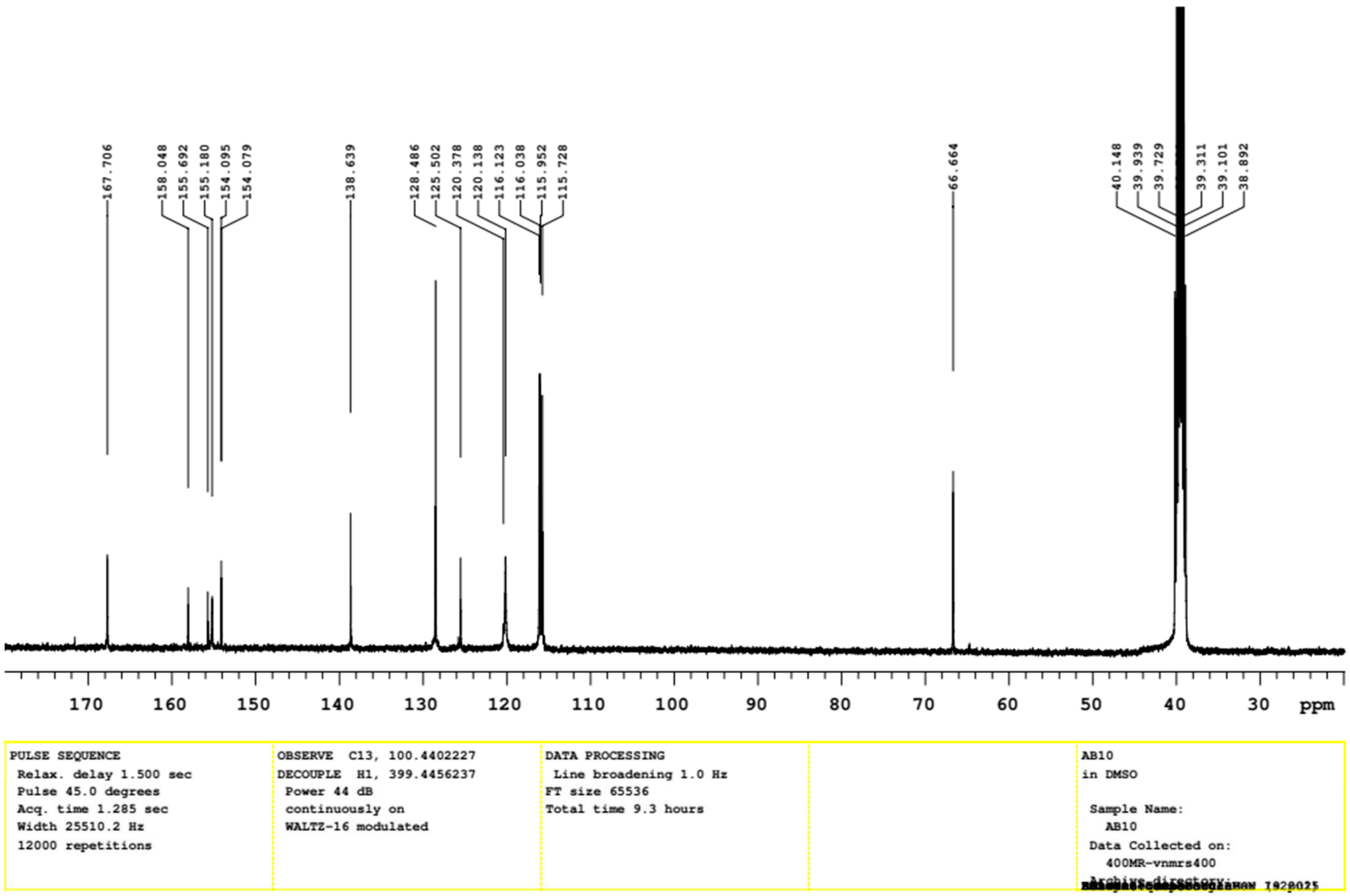

Figure S29: ^{13}C-NMR spectrum of compound **AB10**.

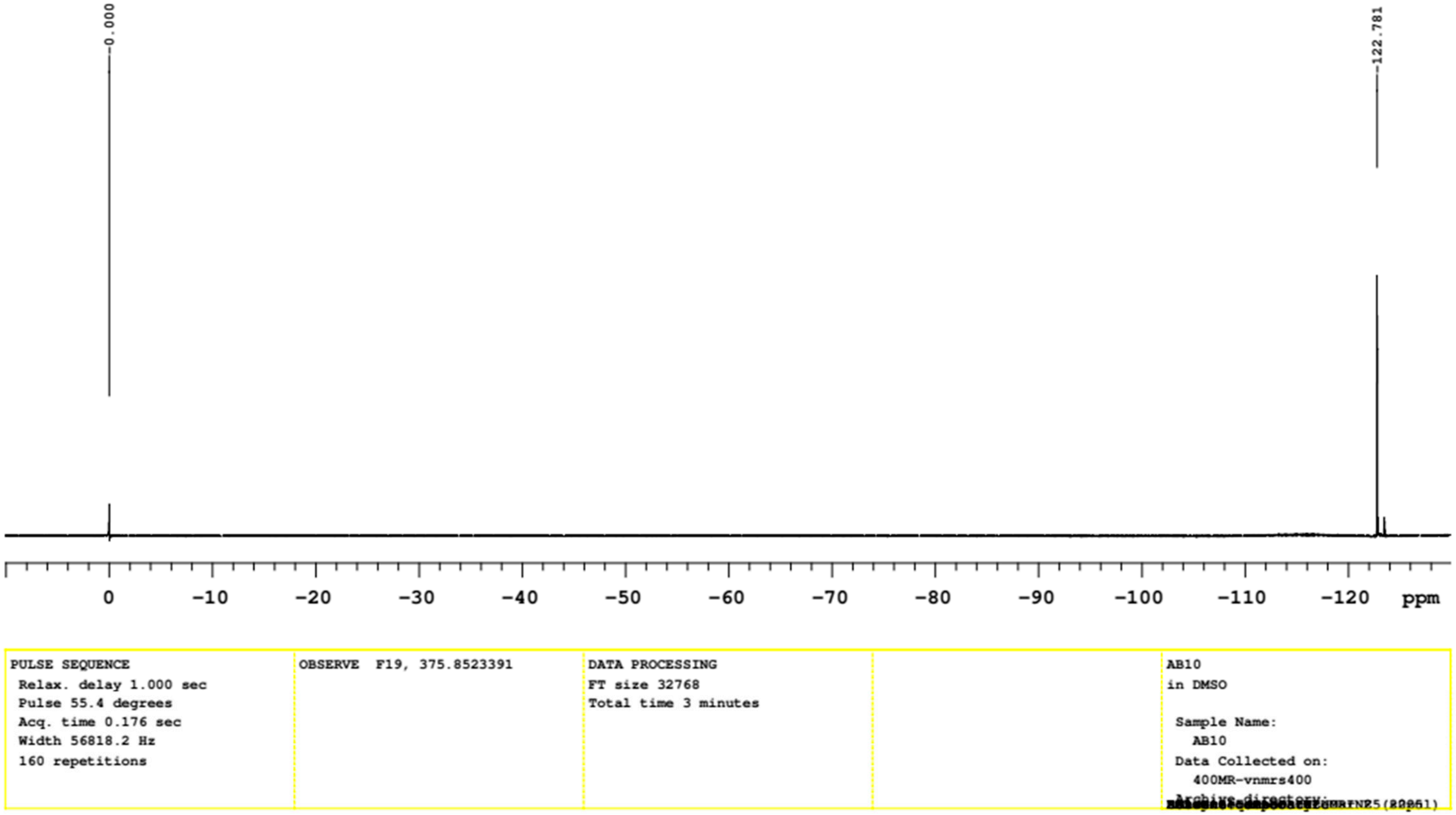

Figure S30: ^{19}F-NMR spectrum of compound **AB10.**

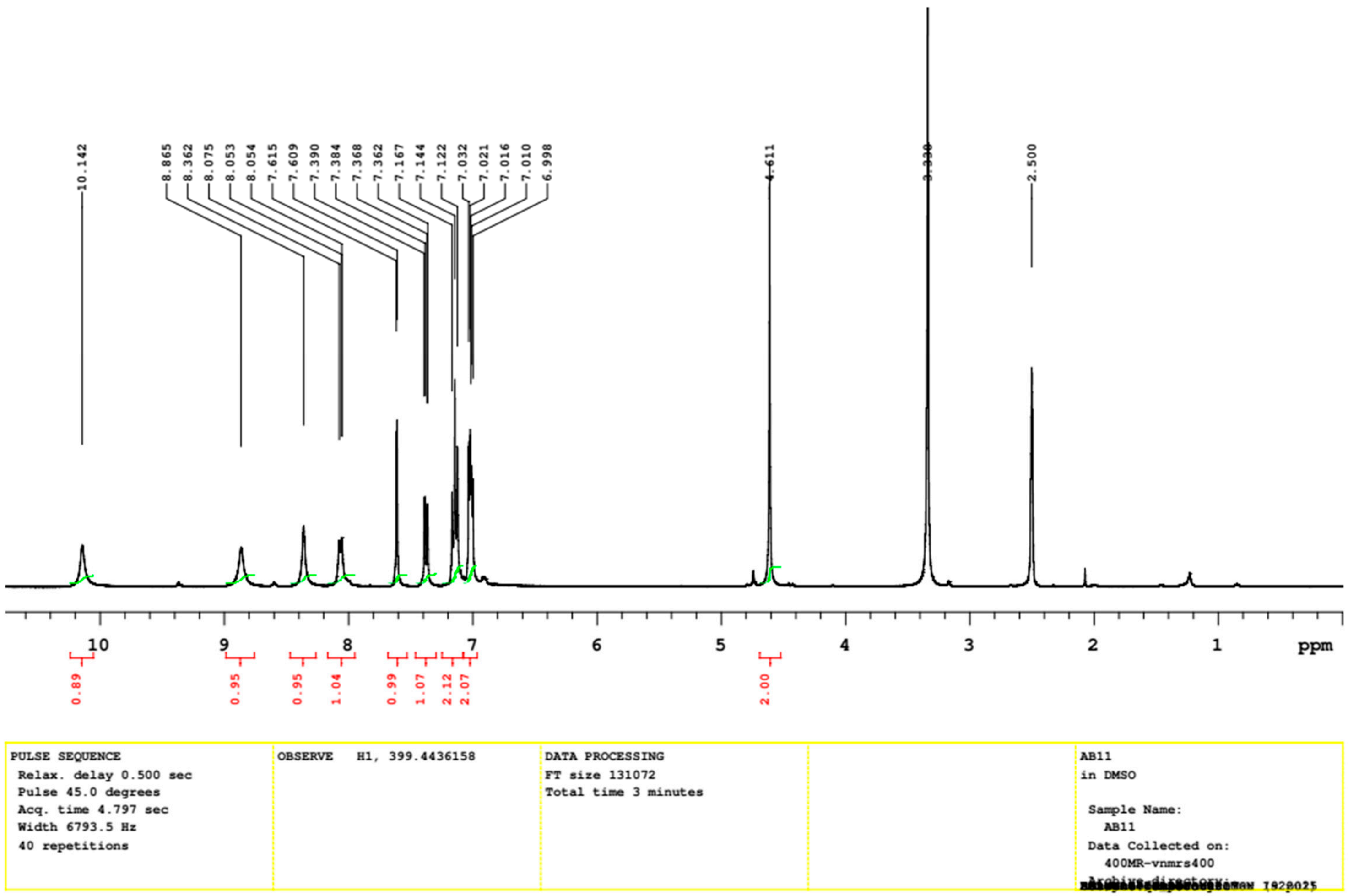

Figure S31: ^{1}H-NMR spectrum of compound **AB11**.

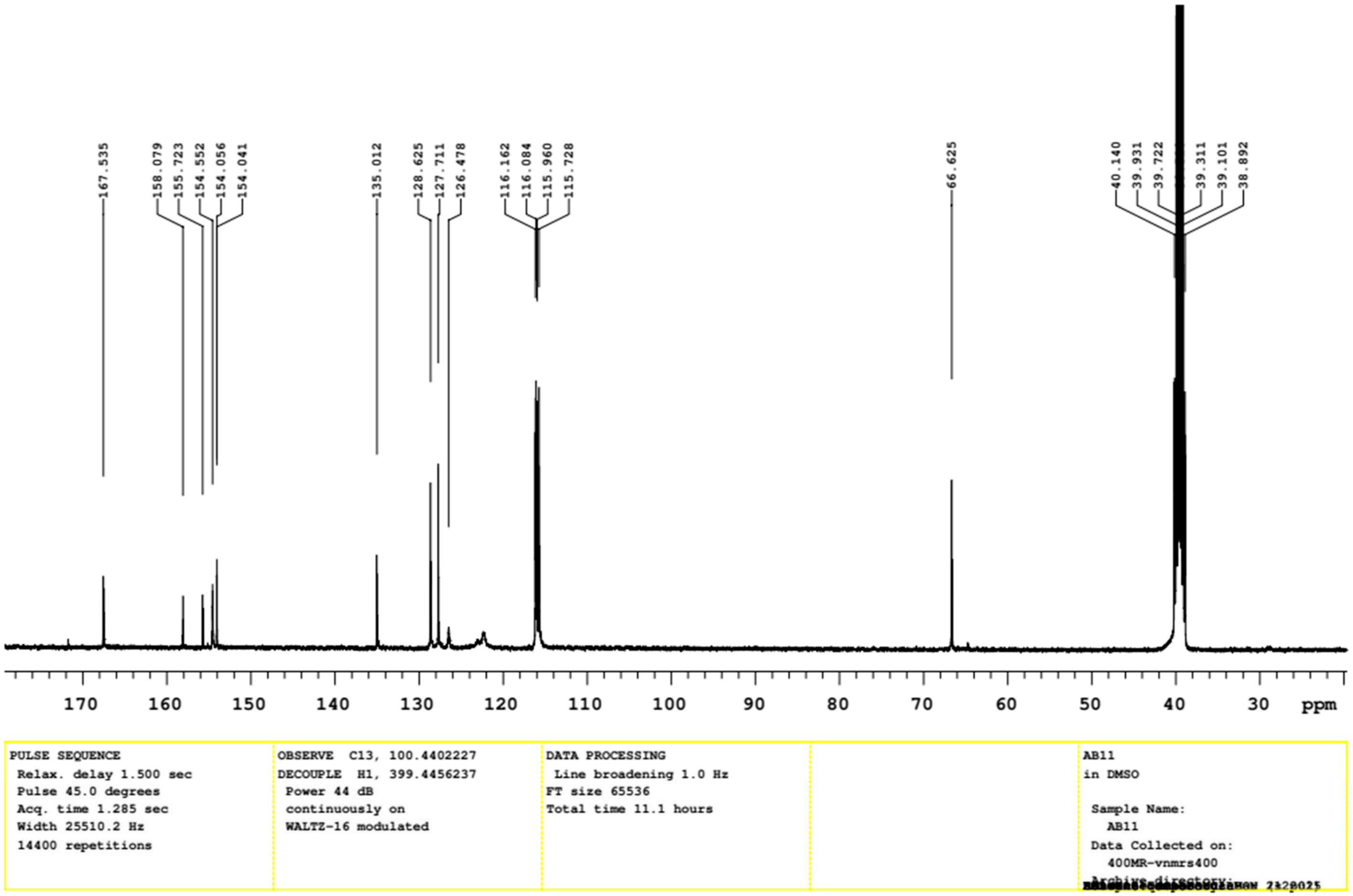

Figure S32: ^{13}C-NMR spectrum of compound **AB11**.

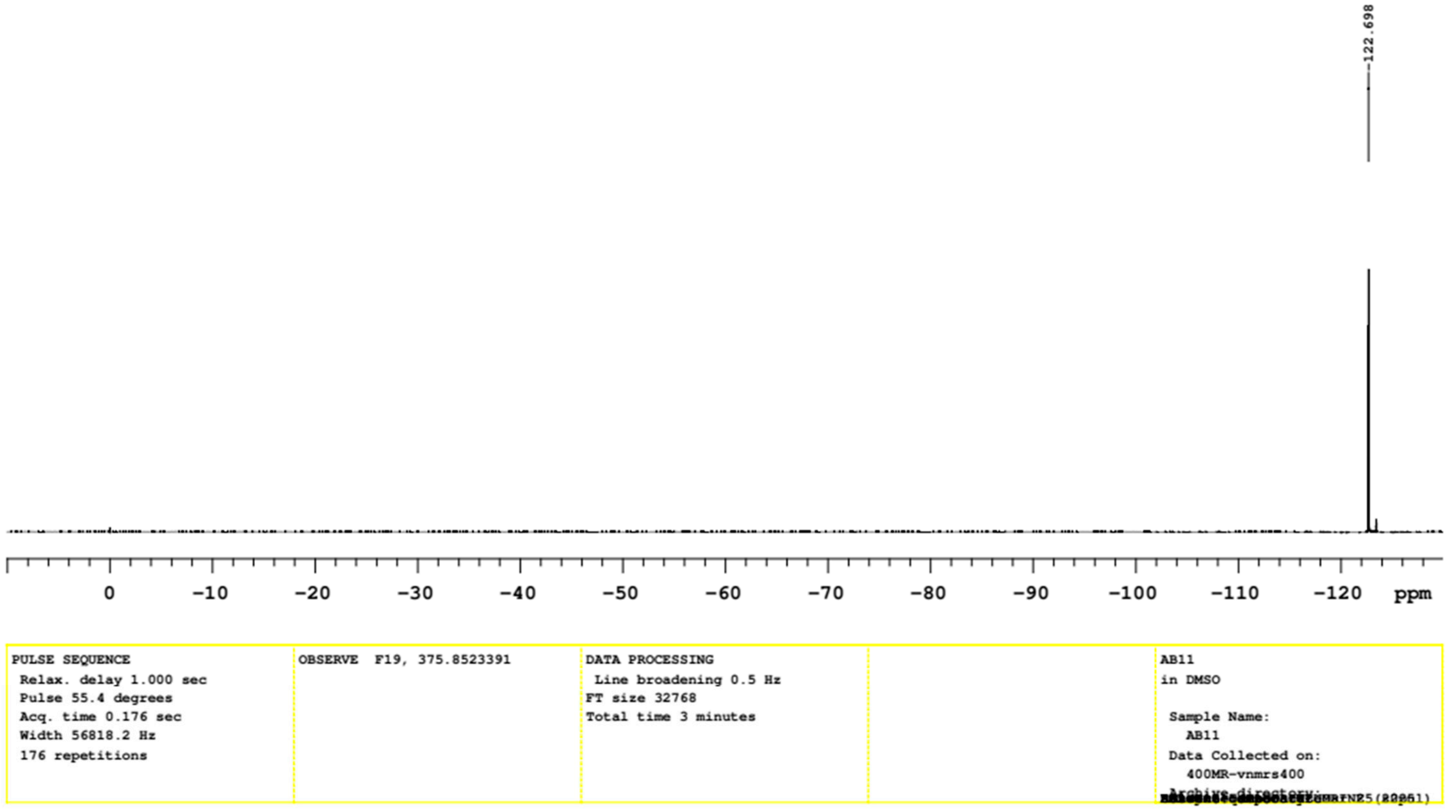

Figure S33: ^{19}F-NMR spectrum of compound **AB11.**

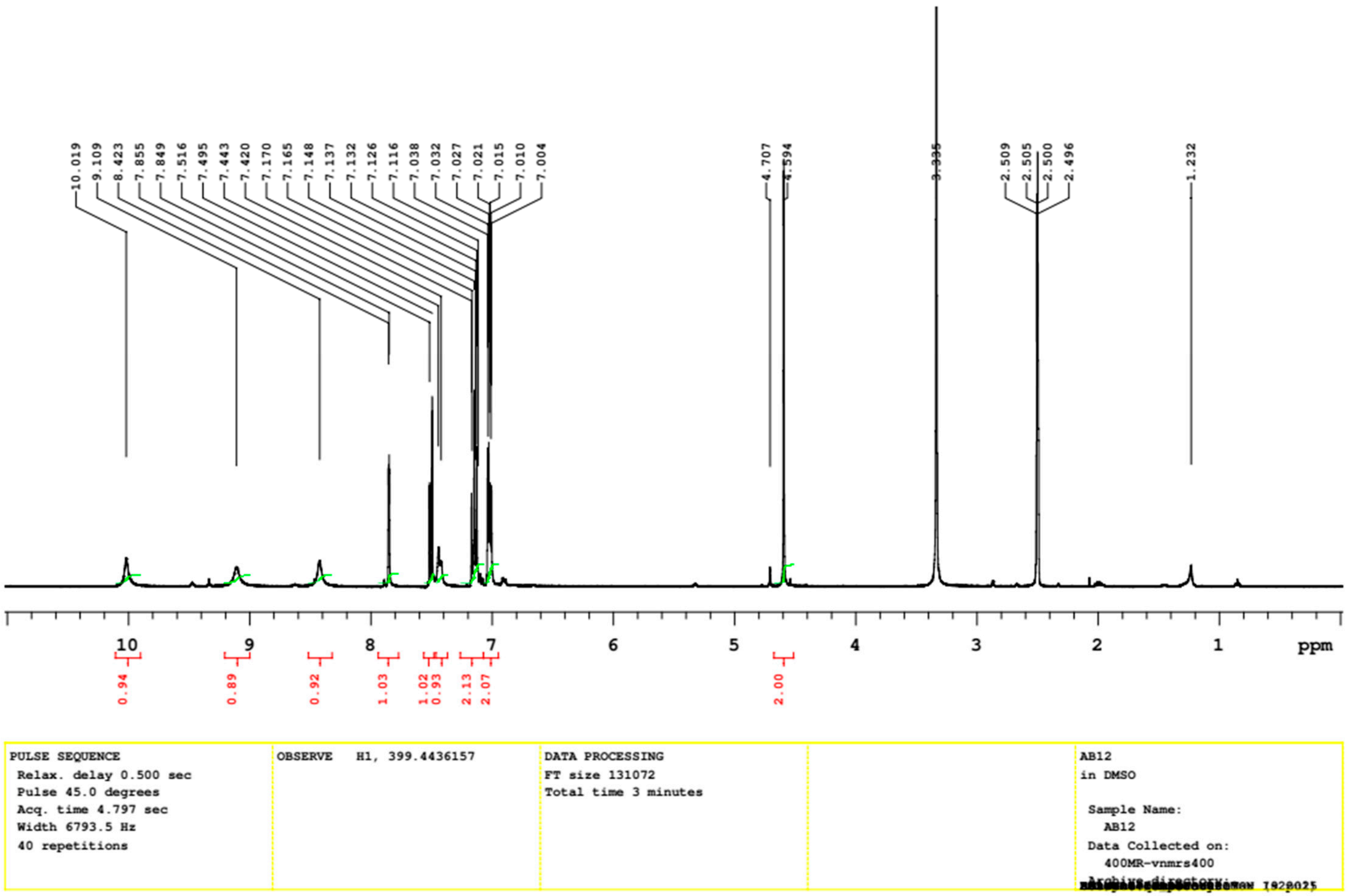

Figure S34: ^{1}H-NMR spectrum of compound **AB12**.

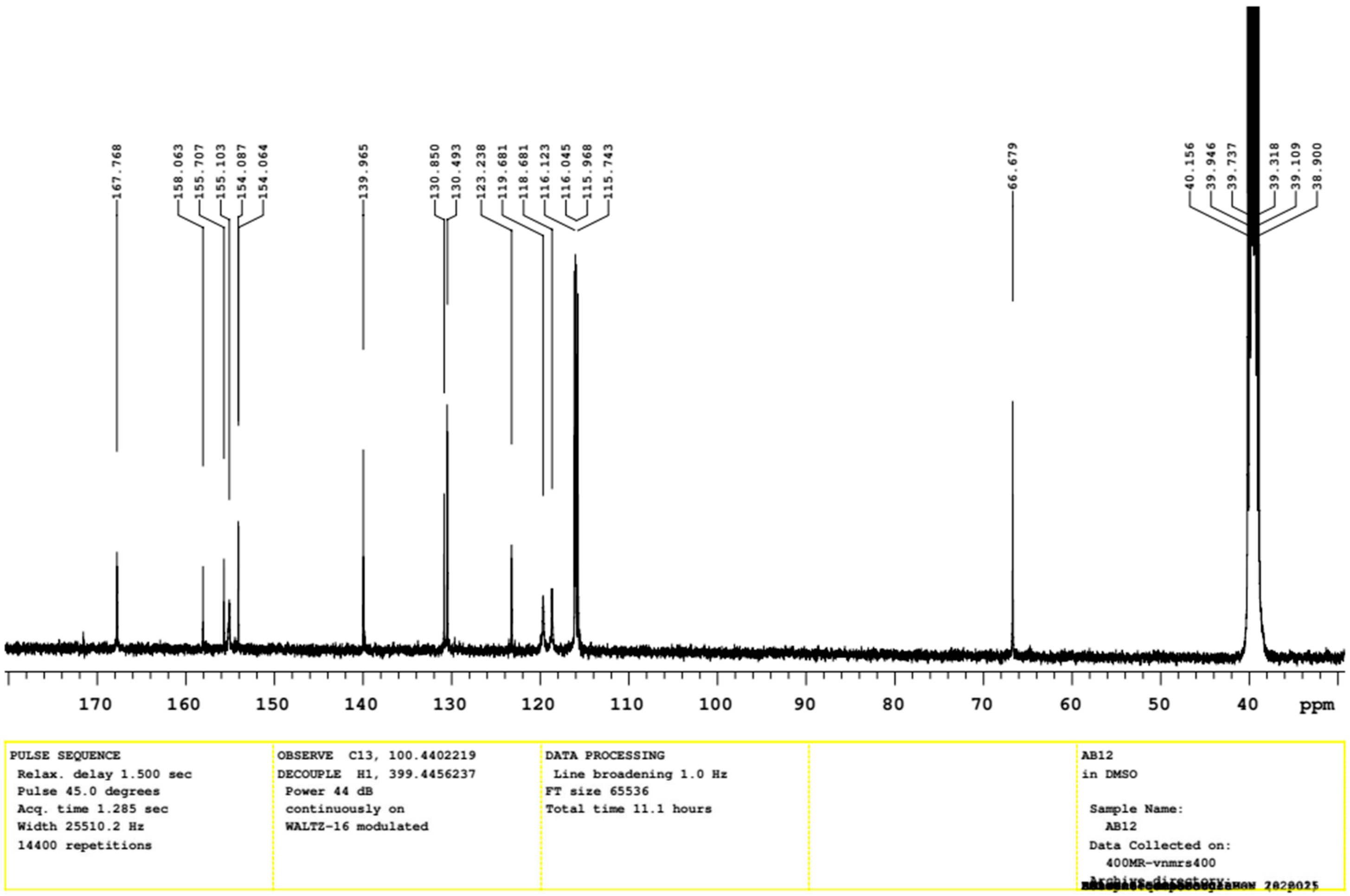

Figure S35: ^{13}C-NMR spectrum of compound **AB12**.

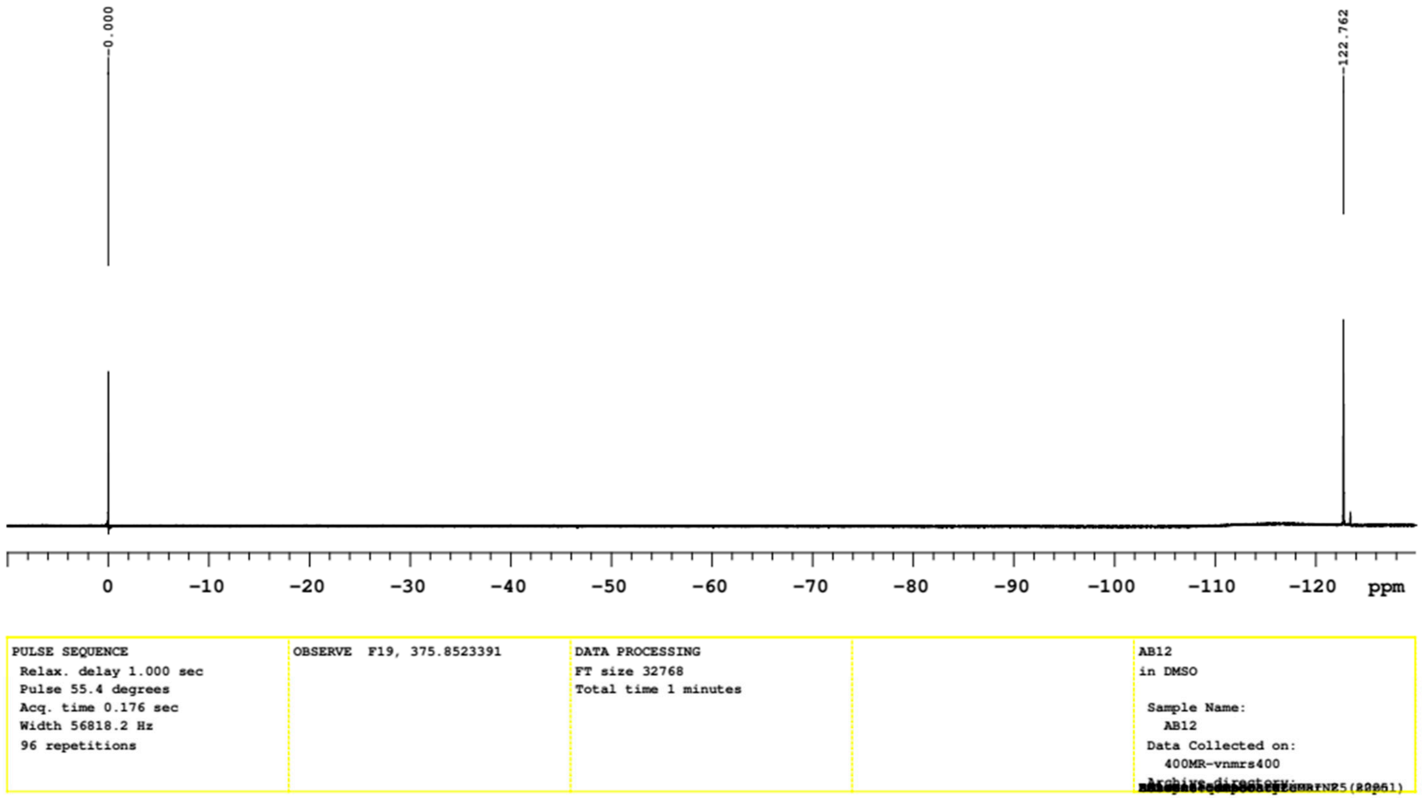

Figure S36: ^{19}F-NMR spectrum of compound **AB12.**

MS analysis

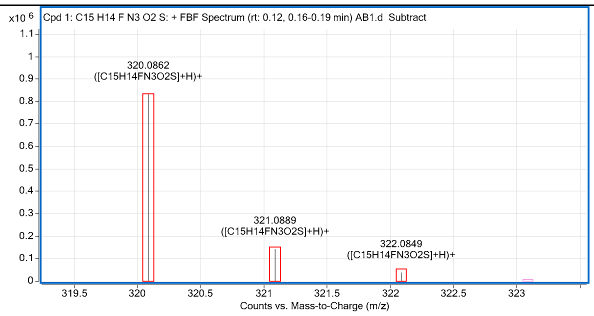

x10 6
Cpd 1: C15 H14 F N3 O2 S: + FBF Spectrum (rt: 0.12, 0.16-0.19 min) AB1.d Subtract
320.0862
([C15H14FN3O2S]+H)+
321.0889
([C15H14FN3O2S]+H)+
322.0849
([C15H14FN3O2S]+H)+
Counts vs. Mass-to-Charge (m/z)

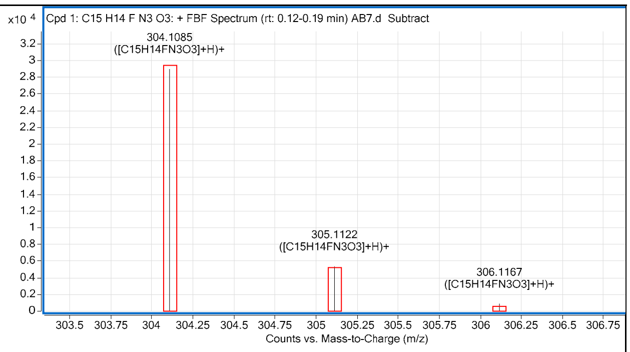

x10 4
Cpd 1: C15 H14 F N3 O3: + FBF Spectrum (rt: 0.12-0.19 min) AB7.d Subtract
304.1085
([C15H14FN3O3]+H)+
305.1122
([C15H14FN3O3]+H)+
306.1167
([C15H14FN3O3]+H)+
Counts vs. Mass-to-Charge (m/z)

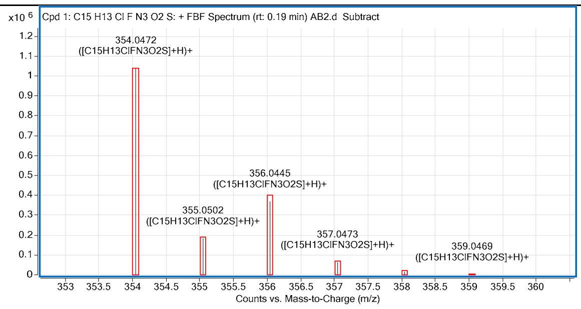

x10 6
Cpd 1: C15 H13 Cl F N3 O2 S: + FBF Spectrum (rt: 0.19 min) AB2.d Subtract
354.0472
([C15H13ClFN3O2S]+H)+
356.0445
([C15H13ClFN3O2S]+H)+
355.0502
([C15H13ClFN3O2S]+H)+
357.0473
([C15H13ClFN3O2S]+H)+
359.0469
([C15H13ClFN3O2S]+H)+
Counts vs. Mass-to-Charge (m/z)

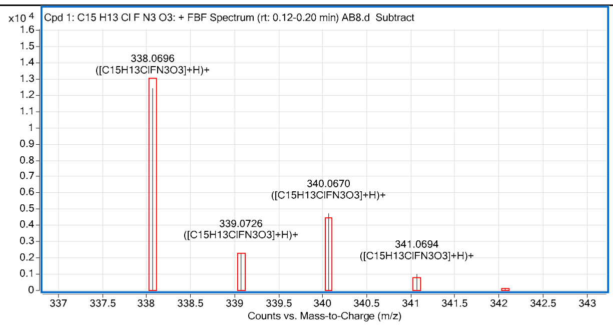

x10 4
Cpd 1: C15 H13 Cl F N3 O3: + FBF Spectrum (rt: 0.12-0.20 min) AB8.d Subtract
338.0696
([C15H13ClFN3O3]+H)+
340.0670
([C15H13ClFN3O3]+H)+
339.0726
([C15H13ClFN3O3]+H)+
341.0694
([C15H13ClFN3O3]+H)+
Counts vs. Mass-to-Charge (m/z)

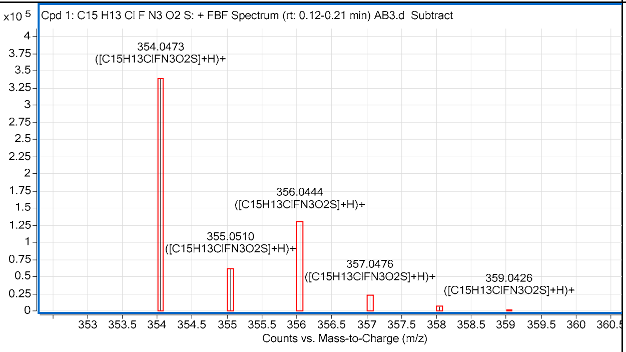

x10 5
Cpd 1: C15 H13 Cl F N3 O2 S: + FBF Spectrum (rt: 0.12-0.21 min) AB3.d Subtract
354.0473
([C15H13ClFN3O2S]+H)+
356.0444
([C15H13ClFN3O2S]+H)+
355.0510
([C15H13ClFN3O2S]+H)+
357.0476
([C15H13ClFN3O2S]+H)+
359.0426
([C15H13ClFN3O2S]+H)+
Counts vs. Mass-to-Charge (m/z)

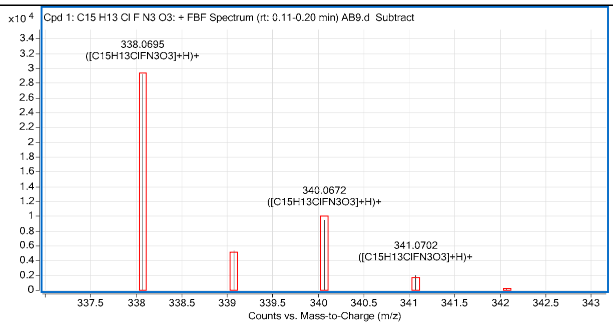

x10 4
Cpd 1: C15 H13 Cl F N3 O3: + FBF Spectrum (rt: 0.11-0.20 min) AB9.d Subtract
338.0695
([C15H13ClFN3O3]+H)+
340.0672
([C15H13ClFN3O3]+H)+
341.0702
([C15H13ClFN3O3]+H)+
Counts vs. Mass-to-Charge (m/z)

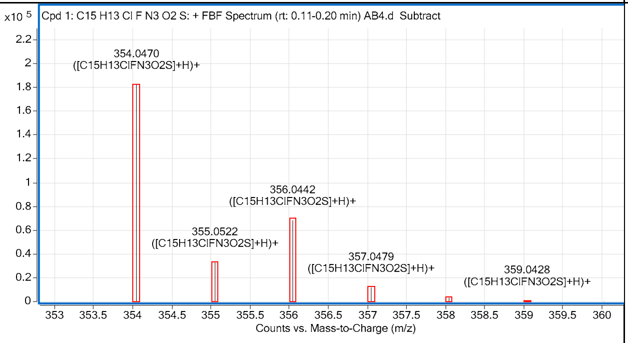

x10 5
Cpd 1: C15 H13 Cl F N3 O2 S: + FBF Spectrum (rt: 0.11-0.20 min) AB4.d Subtract
354.0470
([C15H13ClFN3O2S]+H)+
356.0442
([C15H13ClFN3O2S]+H)+
355.0522
([C15H13ClFN3O2S]+H)+
357.0479
([C15H13ClFN3O2S]+H)+
359.0428
([C15H13ClFN3O2S]+H)+
Counts vs. Mass-to-Charge (m/z)

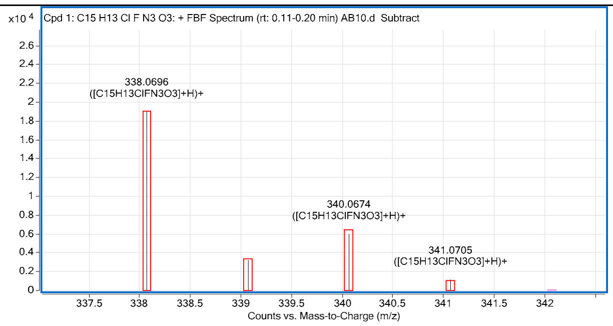

x10 4
Cpd 1: C15 H13 Cl F N3 O3: + FBF Spectrum (rt: 0.11-0.20 min) AB10.d Subtract
338.0696
([C15H13ClFN3O3]+H)+
340.0674
([C15H13ClFN3O3]+H)+
341.0705
([C15H13ClFN3O3]+H)+
Counts vs. Mass-to-Charge (m/z)

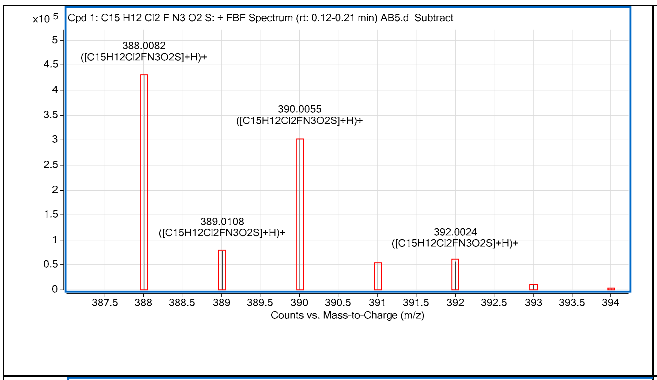

Cpd 1: C15 H12 Cl2 F N3 O2 S: + FBF Spectrum (rt: 0.12-0.21 min) AB5.d Subtract
388.0082 ([C15H12Cl2FN3O2S]+H)+
389.0108 ([C15H12Cl2FN3O2S]+H)+
390.0055 ([C15H12Cl2FN3O2S]+H)+
392.0024 ([C15H12Cl2FN3O2S]+H)+
Counts vs. Mass-to-Charge (m/z)

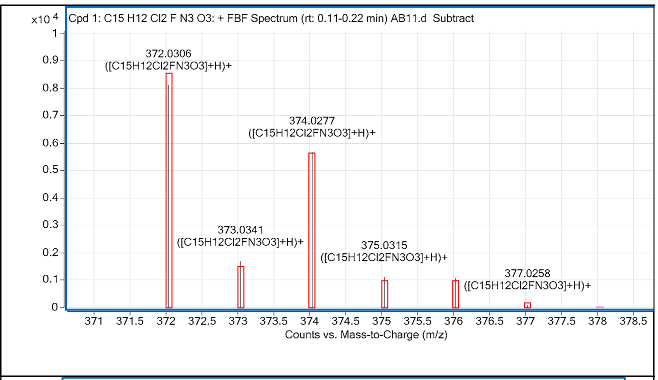

Cpd 1: C15 H12 Cl2 F N3 O3: + FBF Spectrum (rt: 0.11-0.22 min) AB11.d Subtract
372.0306 ([C15H12Cl2FN3O3]+H)+
373.0341 ([C15H12Cl2FN3O3]+H)+
374.0277 ([C15H12Cl2FN3O3]+H)+
375.0315 ([C15H12Cl2FN3O3]+H)+
377.0258 ([C15H12Cl2FN3O3]+H)+
Counts vs. Mass-to-Charge (m/z)

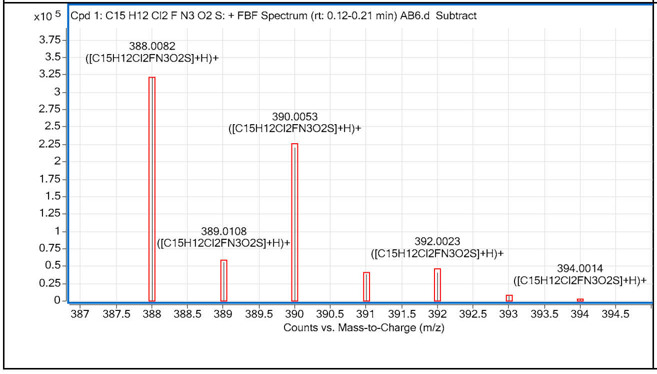

Cpd 1: C15 H12 Cl2 F N3 O2 S: + FBF Spectrum (rt: 0.12-0.21 min) AB6.d Subtract
388.0082 ([C15H12Cl2FN3O2S]+H)+
389.0108 ([C15H12Cl2FN3O2S]+H)+
390.0053 ([C15H12Cl2FN3O2S]+H)+
392.0023 ([C15H12Cl2FN3O2S]+H)+
394.0014 ([C15H12Cl2FN3O2S]+H)+
Counts vs. Mass-to-Charge (m/z)

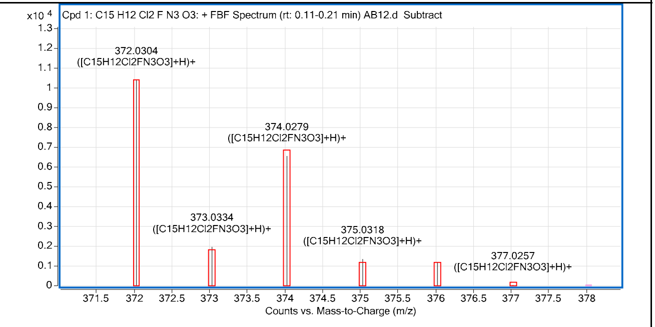

Cpd 1: C15 H12 Cl2 F N3 O3: + FBF Spectrum (rt: 0.11-0.21 min) AB12.d Subtract
372.0304 ([C15H12Cl2FN3O3]+H)+
373.0334 ([C15H12Cl2FN3O3]+H)+
374.0279 ([C15H12Cl2FN3O3]+H)+
375.0318 ([C15H12Cl2FN3O3]+H)+
377.0257 ([C15H12Cl2FN3O3]+H)+
Counts vs. Mass-to-Charge (m/z)

4.7 MOLECULAR DOCKING

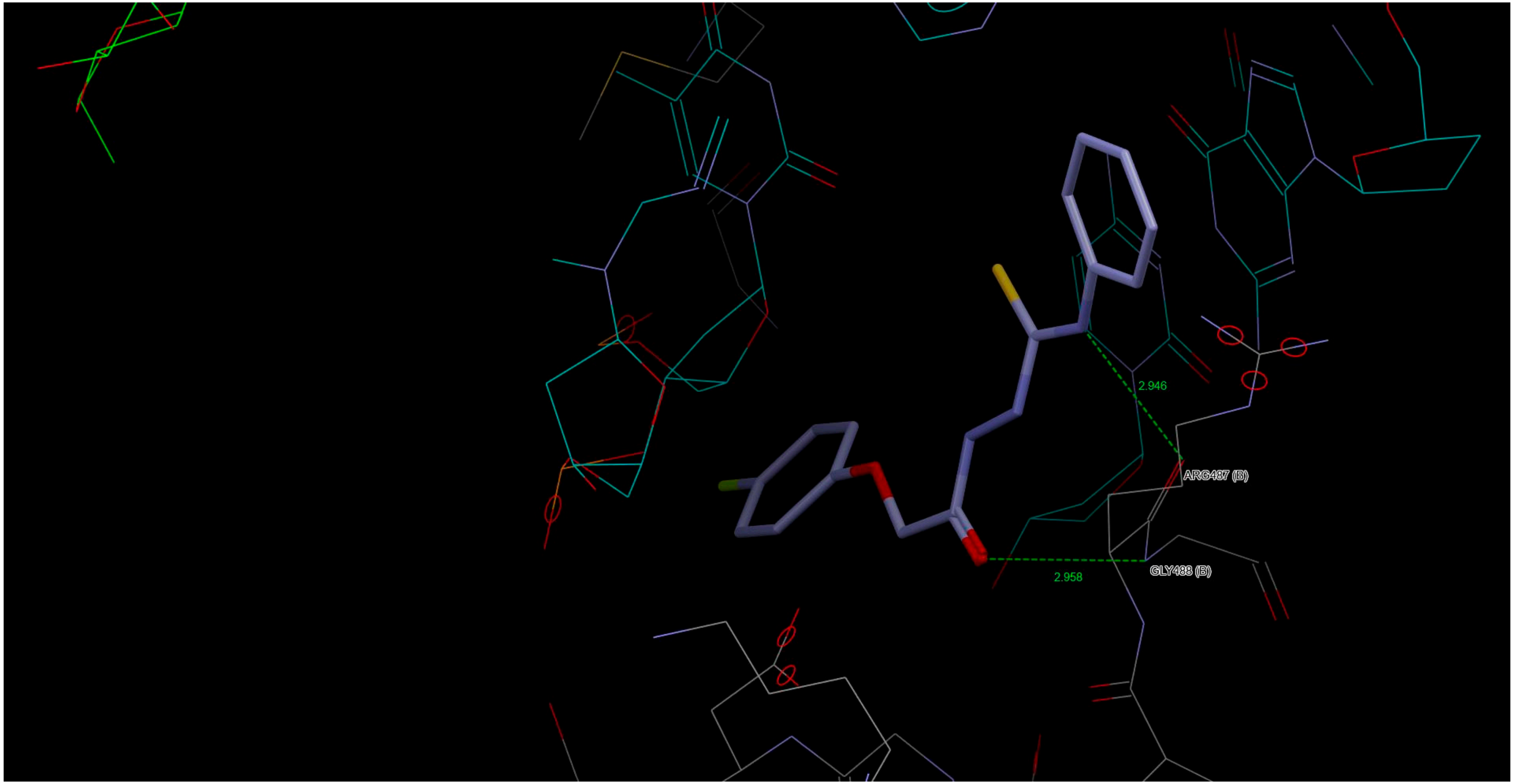

Figure S37. Interactions of **AB1** with the active site of Topoisomerase IIα.

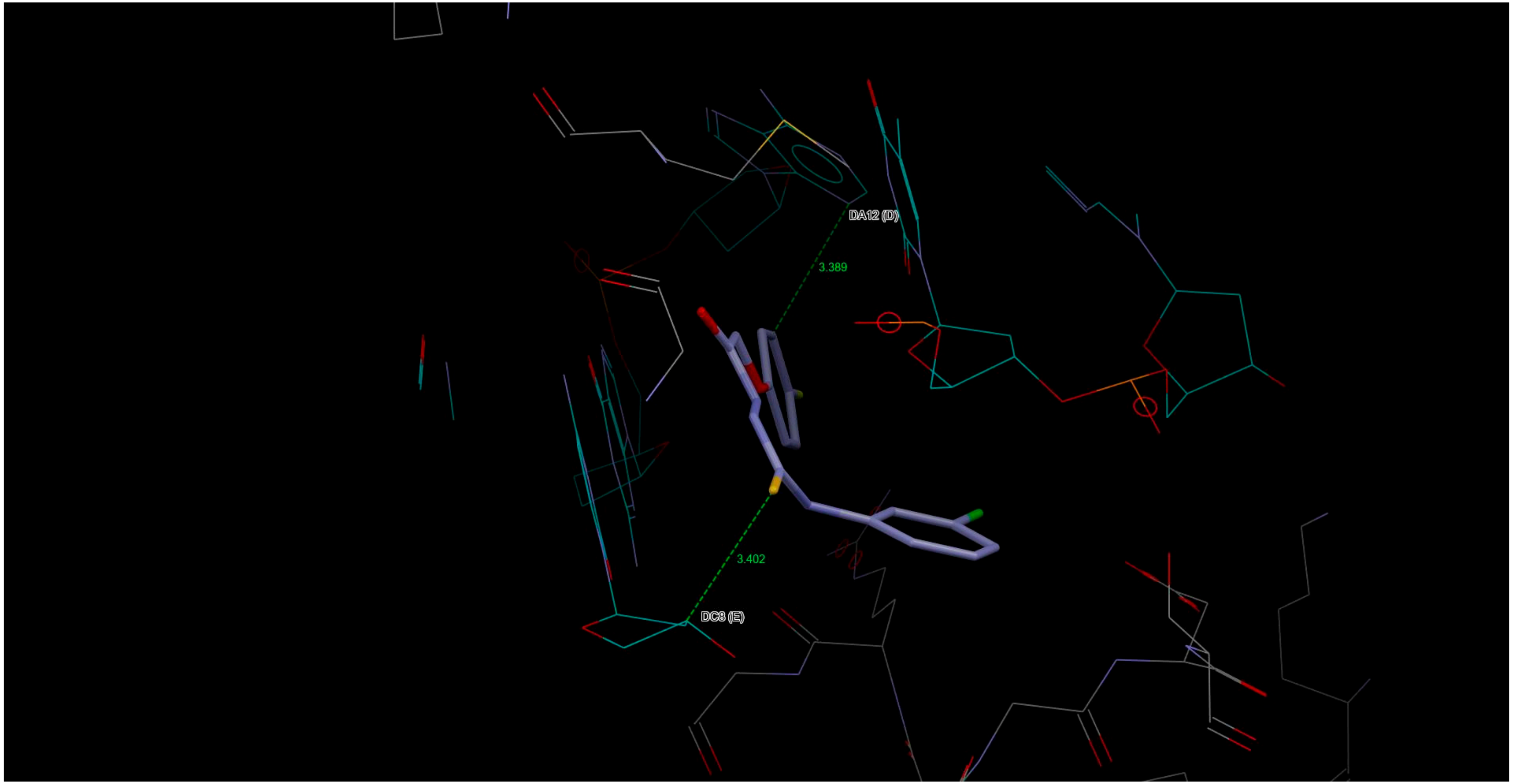

Figure S38. Interactions of **AB3** with the active site of Topoisomerase IIα.

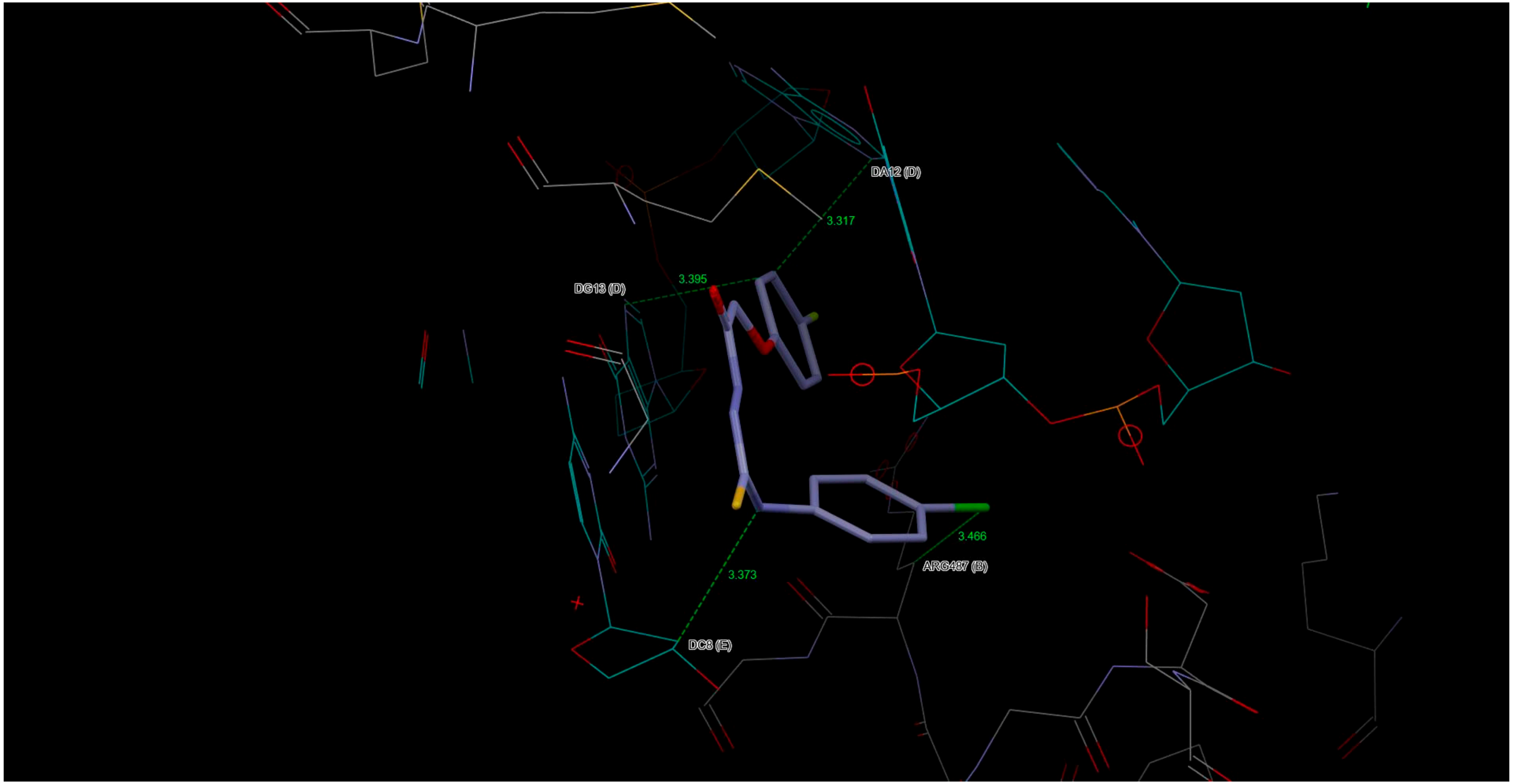

Figure S39. Interactions of **AB4** with the active site of Topoisomerase IIα.

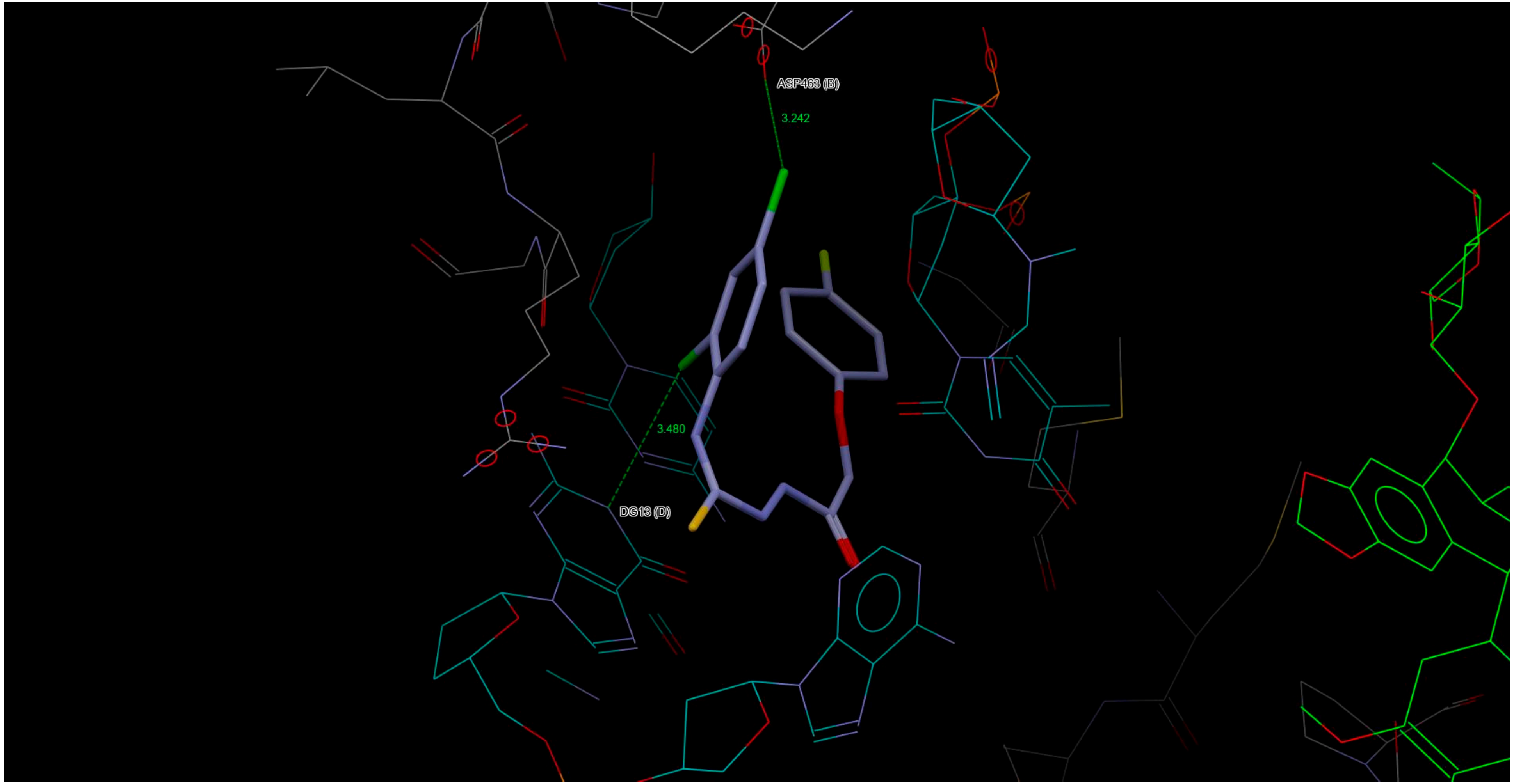

Figure S40. Interactions of **AB5** with the active site of Topoisomerase IIα.

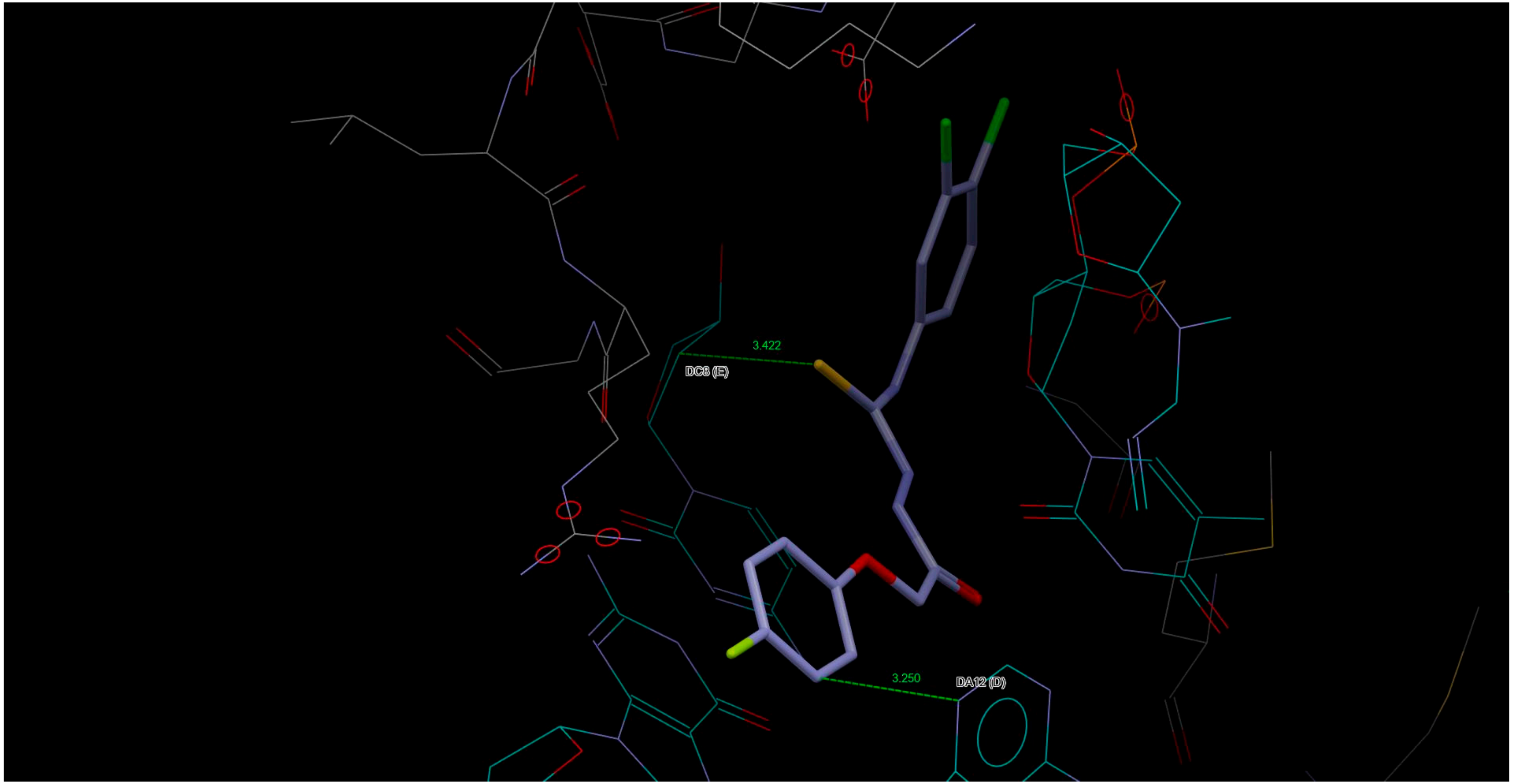

Figure S41. Interactions of **AB6** with the active site of Topoisomerase IIα.

CIF AB2

data_shelx

_audit_creation_method 'SHELXL-2014/7'

_shelx_SHELXL_version_number '2014/7'

_chemical_name_systematic ?

_chemical_name_common ?

_chemical_melting_point ?

_chemical_formula_moiety 'C15 H13 Cl F N3 O2 S'

_chemical_formula_sum 'C15 H13 Cl F N3 O2 S'

_chemical_formula_weight 353.79

loop_

_atom_type_symbol

_atom_type_description

_atom_type_scat_dispersion_real

_atom_type_scat_dispersion_imag

_atom_type_scat_source

'C' 'C' 0.0033 0.0016

'International Tables Vol C Tables 4.2.6.8 and 6.1.1.4'

'H' 'H' 0.0000 0.0000

'International Tables Vol C Tables 4.2.6.8 and 6.1.1.4'

'N' 'N' 0.0061 0.0033

'International Tables Vol C Tables 4.2.6.8 and 6.1.1.4'

'O' 'O' 0.0106 0.0060

'International Tables Vol C Tables 4.2.6.8 and 6.1.1.4'

'F' 'F' 0.0171 0.0103

'International Tables Vol C Tables 4.2.6.8 and 6.1.1.4'

'S' 'S' 0.1246 0.1234

'International Tables Vol C Tables 4.2.6.8 and 6.1.1.4'

'Cl' 'Cl' 0.1484 0.1585

'International Tables Vol C Tables 4.2.6.8 and 6.1.1.4'

_space_group_crystal_system triclinic

_space_group_IT_number 2

_space_group_name_H-M_alt 'P -1'

_space_group_name_Hall '-P 1'

_shelx_space_group_comment

;

The symmetry employed for this shelxl refinement is uniquely defined

by the following loop, which should always be used as a source of

symmetry information in preference to the above space-group names.

They are only intended as comments.

;

loop_

 _space_group_symop_operation_xyz

 'x, y, z'

 '-x, -y, -z'

_cell_length_a 8.6773(15)

_cell_length_b 9.3488(16)

_cell_length_c 10.6905(15)

_cell_angle_alpha 89.835(13)

_cell_angle_beta 70.387(14)

_cell_angle_gamma 76.972(15)

_cell_volume 793.4(2)

_cell_formula_units_Z 2

_cell_measurement_temperature 293(2)

_cell_measurement_reflns_used 785

_cell_measurement_theta_min 2.6370

_cell_measurement_theta_max 24.2380

_exptl_crystal_description plate

_exptl_crystal_colour colourless

_exptl_crystal_density_meas ?

_exptl_crystal_density_method ?

_exptl_crystal_density_diffrn 1.481

_exptl_crystal_F_000 364

_exptl_transmission_factor_min ?

_exptl_transmission_factor_max ?

_exptl_crystal_size_max 0.30

_exptl_crystal_size_mid 0.20

_exptl_crystal_size_min 0.10

_exptl_absorpt_coefficient_mu 0.395

_shelx_estimated_absorpt_T_min ?

_shelx_estimated_absorpt_T_max ?

_exptl_absorpt_correction_T_min 0.60935

_exptl_absorpt_correction_T_max 1.00000

_exptl_absorpt_correction_type multi-scan

_exptl_absorpt_process_details

;

CrysAlisPro 1.171.39.46 (Rigaku Oxford Diffraction, 2018)

Empirical absorption correction using spherical harmonics,

implemented in SCALE3 ABSPACK scaling algorithm.

;

_exptl_absorpt_special_details ?

_diffrn_ambient_temperature 293(2)

_diffrn_radiation_wavelength 0.71073

_diffrn_radiation_type MoK\a

_diffrn_source ?

_diffrn_measurement_device_type 'KM4 CCD four-circle diffractometer'

_diffrn_measurement_method '\w scans'

_diffrn_detector_area_resol_mean ?

_diffrn_reflns_number 6495

_diffrn_reflns_av_unetI/netI 0.0761

_diffrn_reflns_av_R_equivalents ?

_diffrn_reflns_limit_h_min -11

_diffrn_reflns_limit_h_max 11

_diffrn_reflns_limit_k_min -9

_diffrn_reflns_limit_k_max 11

_diffrn_reflns_limit_l_min -12

_diffrn_reflns_limit_l_max 13

_diffrn_reflns_theta_min 2.029

_diffrn_reflns_theta_max 28.744

_diffrn_reflns_theta_full 25.242

_diffrn_measured_fraction_theta_max 0.847

_diffrn_measured_fraction_theta_full 1.000

_diffrn_reflns_Laue_measured_fraction_max 0.847

_diffrn_reflns_Laue_measured_fraction_full 1.000

_diffrn_reflns_point_group_measured_fraction_max 0.847

_diffrn_reflns_point_group_measured_fraction_full 1.000

_reflns_number_total 6495

_reflns_number_gt 3395

_reflns_threshold_expression 'I > 2\s(I)'

_reflns_Friedel_coverage 0.000

_reflns_Friedel_fraction_max .

_reflns_Friedel_fraction_full .

_reflns_special_details

;

_reflns_Friedel_fraction is defined as the number of unique

Friedel pairs measured divided by the number that would be

possible theoretically, ignoring centric projections and

systematic absences.

;

_computing_data_collection

;

CrysAlisPro, Agilent Technologies,

Version 1.171.37.35h (release 09-02-2015 CrysAlis171 .NET)

(compiled Feb 9 2015,16:26:32)

;

_computing_cell_refinement

;

CrysAlisPro, Agilent Technologies,

Version 1.171.37.35h (release 09-02-2015 CrysAlis171 .NET)

(compiled Feb 9 2015,16:26:32)

;

_computing_data_reduction

;

CrysAlisPro, Agilent Technologies,

Version 1.171.37.35h (release 09-02-2015 CrysAlis171 .NET)

(compiled Feb 9 2015,16:26:32)

;

_computing_structure_solution 'SHELXS-2013/1 (Sheldrick, 2013)'

_computing_structure_refinement 'SHELXL-2014/7 (Sheldrick, 2014)'

_computing_molecular_graphics 'ORTEP3 for Windows (Farrugia, 2012)'

_computing_publication_material 'SHELXL-2014/7 and WINGX (Farrugia, 2012)'

_refine_special_details

;

Refined as a 2-component twin.

;

_refine_ls_structure_factor_coef Fsqd

_refine_ls_matrix_type full

_refine_ls_weighting_scheme calc

_refine_ls_weighting_details

'w=1/[\s^2^(Fo^2^)+(0.0703P)^2^] where P=(Fo^2^+2Fc^2^)/3'

_atom_sites_solution_primary difmap

_atom_sites_solution_secondary difmap

_atom_sites_solution_hydrogens mixed

_refine_ls_hydrogen_treatment mixed

_refine_ls_extinction_method 'SHELXL-2014/7 (Sheldrick 2014'

_refine_ls_extinction_coef 0.017(4)

_refine_ls_extinction_expression

'Fc^*^=kFc[1+0.001xFc^2^\l^3^/sin(2\q)]^-1/4^'

_refine_ls_number_reflns 6495

_refine_ls_number_parameters 219

_refine_ls_number_restraints 0

_refine_ls_R_factor_all 0.1047

_refine_ls_R_factor_gt 0.0523

_refine_ls_wR_factor_ref 0.1320

_refine_ls_wR_factor_gt 0.1190

_refine_ls_goodness_of_fit_ref 0.861

_refine_ls_restrained_S_all 0.861

_refine_ls_shift/su_max 0.000

_refine_ls_shift/su_mean 0.000

loop_

_atom_site_label

_atom_site_type_symbol

_atom_site_fract_x

_atom_site_fract_y

_atom_site_fract_z

_atom_site_U_iso_or_equiv

_atom_site_adp_type

_atom_site_occupancy

_atom_site_site_symmetry_order

_atom_site_calc_flag

_atom_site_refinement_flags_posn

_atom_site_refinement_flags_adp

_atom_site_refinement_flags_occupancy

_atom_site_disorder_assembly

_atom_site_disorder_group

C2 C 0.0253(3) 0.8201(3) 0.1089(3) 0.0352(7) Uani 1 1 d . . . . .

C6 C 0.3043(3) 0.5085(3) -0.1058(3) 0.0365(7) Uani 1 1 d . . . . .

C8 C 0.4908(3) 0.4649(3) -0.1845(3) 0.0425(8) Uani 1 1 d . . . . .

H8A H 0.5411 0.3715 -0.1583 0.064 Uiso 1 1 calc R U . . .

H8B H 0.5085 0.4537 -0.2789 0.064 Uiso 1 1 calc R U . . .

C21 C -0.2381(3) 0.9676(3) 0.2784(3) 0.0379(7) Uani 1 1 d . . . . .

C22 C -0.4021(4) 1.0326(3) 0.2860(3) 0.0431(8) Uani 1 1 d . . . . .

C23 C -0.5025(4) 1.1474(4) 0.3814(4) 0.0599(10) Uani 1 1 d . . . . .

H23 H -0.6117 1.1903 0.3848 0.090 Uiso 1 1 calc R U . . .

C24 C -0.4396(5) 1.1969(4) 0.4703(4) 0.0689(11) Uani 1 1 d . . . . .

H24 H -0.5053 1.2748 0.5331 0.103 Uiso 1 1 calc R U . . .

C25 C -0.2789(5) 1.1311(4) 0.4664(4) 0.0667(11) Uani 1 1 d . . . . .

H25 H -0.2379 1.1633 0.5284 0.100 Uiso 1 1 calc R U . . .

C26 C -0.1777(4) 1.0174(3) 0.3710(3) 0.0515(9) Uani 1 1 d . . . . .

H26 H -0.0692 0.9744 0.3691 0.077 Uiso 1 1 calc R U . . .

C31 C 0.7400(3) 0.5555(3) -0.2255(3) 0.0354(7) Uani 1 1 d . . . . .

C32 C 0.8084(4) 0.6693(3) -0.2033(3) 0.0415(8) Uani 1 1 d . . . . .

H32 H 0.7393 0.7531 -0.1492 0.062 Uiso 1 1 calc R U . . .

C33 C 0.9798(4) 0.6584(4) -0.2617(3) 0.0470(8) Uani 1 1 d . . . . .

H33 H 1.0270 0.7343 -0.2480 0.070 Uiso 1 1 calc R U . . .

C34 C 1.0781(4) 0.5329(4) -0.3401(3) 0.0458(8) Uani 1 1 d . . . . .

C35 C 1.0145(4) 0.4189(4) -0.3641(3) 0.0478(8) Uani 1 1 d . . . . .

H35 H 1.0848 0.3356 -0.4182 0.072 Uiso 1 1 calc R U . . .

C36 C 0.8423(3) 0.4301(3) -0.3057(3) 0.0423(8) Uani 1 1 d . . . . .

H36 H 0.7960 0.3540 -0.3204 0.063 Uiso 1 1 calc R U . . .

N1 N -0.1421(3) 0.8482(3) 0.1818(3) 0.0401(7) Uani 1 1 d . . . . .

H1 H -0.199(4) 0.798(3) 0.163(3) 0.060 Uiso 1 1 d . U . . .

N4 N 0.0755(3) 0.6945(3) 0.0276(3) 0.0429(7) Uani 1 1 d . . . . .

H4 H 0.010(4) 0.632(3) 0.030(3) 0.064 Uiso 1 1 d . U . . .

N5 N 0.2439(3) 0.6456(3) -0.0503(3) 0.0429(7) Uani 1 1 d . . . . .

H5 H 0.303(4) 0.718(3) -0.050(3) 0.064 Uiso 1 1 d . U . . .

O7 O 0.2157(2) 0.4198(2) -0.0979(2) 0.0478(6) Uani 1 1 d . . . . .

O9 O 0.5680(2) 0.5762(2) -0.1598(2) 0.0457(6) Uani 1 1 d . . . . .

F34 F 1.2484(2) 0.5205(2) -0.3975(2) 0.0720(6) Uani 1 1 d . . . . .

S3 S 0.15909(10) 0.92194(9) 0.11151(9) 0.0535(3) Uani 1 1 d . . . . .

Cl22 Cl -0.48819(10) 0.96503(10) 0.17952(9) 0.0627(3) Uani 1 1 d . . . . .

loop_

_atom_site_aniso_label

_atom_site_aniso_U_11

_atom_site_aniso_U_22

_atom_site_aniso_U_33

_atom_site_aniso_U_23

_atom_site_aniso_U_13

_atom_site_aniso_U_12

C2 0.0300(16) 0.0378(18) 0.0408(17) 0.0014(14) -0.0151(13) -0.0096(13)

C6 0.0311(16) 0.0432(19) 0.0386(18) -0.0015(14) -0.0139(14) -0.0124(15)

C8 0.0318(17) 0.0451(19) 0.0507(19) -0.0076(15) -0.0098(14) -0.0160(14)

C21 0.0333(17) 0.0347(17) 0.0438(18) 0.0011(14) -0.0067(14) -0.0142(14)

C22 0.0342(17) 0.0413(19) 0.0502(19) 0.0049(15) -0.0087(14) -0.0106(15)

C23 0.043(2) 0.047(2) 0.069(3) 0.0001(18) -0.0010(18) 0.0017(17)

C24 0.068(3) 0.052(2) 0.067(3) -0.0190(19) 0.001(2) -0.012(2)

C25 0.067(3) 0.066(3) 0.065(2) -0.020(2) -0.010(2) -0.030(2)

C26 0.044(2) 0.053(2) 0.060(2) -0.0088(17) -0.0164(17) -0.0199(17)

C31 0.0256(16) 0.0403(18) 0.0401(17) 0.0041(14) -0.0098(13) -0.0097(14)

C32 0.0336(17) 0.0379(18) 0.053(2) 0.0003(14) -0.0142(15) -0.0104(14)

C33 0.0365(19) 0.051(2) 0.059(2) 0.0087(17) -0.0190(16) -0.0184(16)

C34 0.0266(17) 0.058(2) 0.052(2) 0.0132(16) -0.0090(15) -0.0143(16)

C35 0.0352(18) 0.047(2) 0.051(2) 0.0029(16) -0.0068(15) -0.0024(16)

C36 0.0349(18) 0.0426(19) 0.0472(19) -0.0036(15) -0.0104(15) -0.0108(15)

N1 0.0276(14) 0.0395(16) 0.0542(17) -0.0088(12) -0.0128(12) -0.0120(11)

N4 0.0235(14) 0.0471(17) 0.0558(17) -0.0099(13) -0.0067(12) -0.0146(12)

N5 0.0243(13) 0.0447(17) 0.0572(17) -0.0085(13) -0.0065(12) -0.0146(12)

O7 0.0318(12) 0.0514(14) 0.0614(15) -0.0086(11) -0.0110(10) -0.0197(10)

O9 0.0245(11) 0.0435(13) 0.0643(14) -0.0117(10) -0.0059(10) -0.0129(9)

F34 0.0266(10) 0.0889(16) 0.0912(15) 0.0113(12) -0.0068(10) -0.0160(10)

S3 0.0347(5) 0.0474(5) 0.0795(7) -0.0082(4) -0.0135(4) -0.0212(4)

Cl22 0.0384(5) 0.0781(7) 0.0719(6) -0.0015(5) -0.0252(4) -0.0049(4)

_geom_special_details

;

All esds (except the esd in the dihedral angle between two l.s. planes)

are estimated using the full covariance matrix. The cell esds are taken

into account individually in the estimation of esds in distances, angles

and torsion angles; correlations between esds in cell parameters are only

used when they are defined by crystal symmetry. An approximate (isotropic)

treatment of cell esds is used for estimating esds involving l.s. planes.

;

loop_

_geom_bond_atom_site_label_1

_geom_bond_atom_site_label_2

_geom_bond_distance

_geom_bond_site_symmetry_2

_geom_bond_publ_flag

C2 N4 1.358(3) . ?

C2 N1 1.362(3) . ?

C2 S3 1.666(3) . ?

C6 O7 1.237(3) . ?

C6 N5 1.327(3) . ?

C6 C8 1.511(4) . ?

C8 O9 1.427(3) . ?

C8 H8A 0.9700 . ?

C8 H8B 0.9700 . ?

C21 C26 1.390(4) . ?

C21 C22 1.391(4) . ?

C21 N1 1.421(4) . ?

C22 C23 1.393(4) . ?

C22 Cl22 1.743(3) . ?

C23 C24 1.371(5) . ?

C23 H23 0.9300 . ?

C24 C25 1.378(5) . ?

C24 H24 0.9300 . ?

C25 C26 1.387(4) . ?

C25 H25 0.9300 . ?

C26 H26 0.9300 . ?

C31 C32 1.386(4) . ?

C31 C36 1.387(4) . ?

C31 O9 1.387(3) . ?

C32 C33 1.385(4) . ?

C32 H32 0.9300 . ?

C33 C34 1.371(4) . ?

C33 H33 0.9300 . ?

C34 C35 1.367(4) . ?

C34 F34 1.374(3) . ?

C35 C36 1.392(4) . ?

C35 H35 0.9300 . ?

C36 H36 0.9300 . ?

N1 H1 0.83(3) . ?

N4 N5 1.386(3) . ?

N4 H4 0.90(3) . ?

N5 H5 0.94(3) . ?

loop_

_geom_angle_atom_site_label_1

_geom_angle_atom_site_label_2

_geom_angle_atom_site_label_3

_geom_angle

_geom_angle_site_symmetry_1

_geom_angle_site_symmetry_3

_geom_angle_publ_flag

N4 C2 N1 111.3(2) . . ?

N4 C2 S3 121.7(2) . . ?

N1 C2 S3 127.0(2) . . ?

O7 C6 N5 123.4(3) . . ?

O7 C6 C8 120.4(3) . . ?

N5 C6 C8 116.3(2) . . ?

O9 C8 C6 109.2(2) . . ?

O9 C8 H8A 109.8 . . ?

C6 C8 H8A 109.8 . . ?

O9 C8 H8B 109.8 . . ?

C6 C8 H8B 109.8 . . ?

H8A C8 H8B 108.3 . . ?

C26 C21 C22 118.3(3) . . ?

C26 C21 N1 122.5(3) . . ?

C22 C21 N1 119.1(3) . . ?

C21 C22 C23 121.2(3) . . ?

C21 C22 Cl22 119.9(2) . . ?

C23 C22 Cl22 118.9(3) . . ?

C24 C23 C22 119.6(3) . . ?

C24 C23 H23 120.2 . . ?

C22 C23 H23 120.2 . . ?

C23 C24 C25 119.9(3) . . ?

C23 C24 H24 120.0 . . ?

C25 C24 H24 120.0 . . ?

C24 C25 C26 120.7(3) . . ?

C24 C25 H25 119.6 . . ?

C26 C25 H25 119.6 . . ?

C25 C26 C21 120.2(3) . . ?

C25 C26 H26 119.9 . . ?

C21 C26 H26 119.9 . . ?

C32 C31 C36 120.3(3) . . ?

C32 C31 O9 115.3(2) . . ?

C36 C31 O9 124.3(2) . . ?

C33 C32 C31 120.0(3) . . ?

C33 C32 H32 120.0 . . ?

C31 C32 H32 120.0 . . ?

C34 C33 C32 118.4(3) . . ?

C34 C33 H33 120.8 . . ?

C32 C33 H33 120.8 . . ?

C35 C34 C33 123.0(3) . . ?

C35 C34 F34 118.1(3) . . ?

C33 C34 F34 118.9(3) . . ?

C34 C35 C36 118.6(3) . . ?

C34 C35 H35 120.7 . . ?

C36 C35 H35 120.7 . . ?

C31 C36 C35 119.7(3) . . ?

C31 C36 H36 120.2 . . ?

C35 C36 H36 120.2 . . ?

C2 N1 C21 127.4(2) . . ?

C2 N1 H1 118(2) . . ?

C21 N1 H1 114(2) . . ?

C2 N4 N5 119.3(2) . . ?

C2 N4 H4 124(2) . . ?

N5 N4 H4 116(2) . . ?

C6 N5 N4 120.6(2) . . ?

C6 N5 H5 128.1(19) . . ?

N4 N5 H5 111.2(19) . . ?

C31 O9 C8 117.2(2) . . ?

loop_

_geom_torsion_atom_site_label_1

_geom_torsion_atom_site_label_2

_geom_torsion_atom_site_label_3

_geom_torsion_atom_site_label_4

_geom_torsion

_geom_torsion_site_symmetry_1

_geom_torsion_site_symmetry_2

_geom_torsion_site_symmetry_3

_geom_torsion_site_symmetry_4

_geom_torsion_publ_flag

O7 C6 C8 O9 -170.3(3) . . . . ?

N5 C6 C8 O9 11.1(4) . . . . ?

C26 C21 C22 C23 1.8(4) . . . . ?

N1 C21 C22 C23 178.3(3) . . . . ?

C26 C21 C22 Cl22 -175.5(2) . . . . ?

N1 C21 C22 Cl22 1.1(4) . . . . ?

C21 C22 C23 C24 -0.5(5) . . . . ?

Cl22 C22 C23 C24 176.7(3) . . . . ?

C22 C23 C24 C25 -1.2(6) . . . . ?

C23 C24 C25 C26 1.7(6) . . . . ?

C24 C25 C26 C21 -0.5(5) . . . . ?

C22 C21 C26 C25 -1.3(5) . . . . ?

N1 C21 C26 C25 -177.7(3) . . . . ?

C36 C31 C32 C33 0.2(4) . . . . ?

O9 C31 C32 C33 178.5(3) . . . . ?

C31 C32 C33 C34 -0.4(5) . . . . ?

C32 C33 C34 C35 0.5(5) . . . . ?

C32 C33 C34 F34 -179.5(3) . . . . ?

C33 C34 C35 C36 -0.4(5) . . . . ?

F34 C34 C35 C36 179.6(3) . . . . ?

C32 C31 C36 C35 0.0(5) . . . . ?

O9 C31 C36 C35 -178.2(3) . . . . ?

C34 C35 C36 C31 0.1(5) . . . . ?

N4 C2 N1 C21 178.9(3) . . . . ?

S3 C2 N1 C21 -2.0(5) . . . . ?

C26 C21 N1 C2 -41.5(5) . . . . ?

C22 C21 N1 C2 142.1(3) . . . . ?

N1 C2 N4 N5 -177.5(3) . . . . ?

S3 C2 N4 N5 3.3(4) . . . . ?

O7 C6 N5 N4 3.6(5) . . . . ?

C8 C6 N5 N4 -177.8(3) . . . . ?

C2 N4 N5 C6 164.0(3) . . . . ?

C32 C31 O9 C8 177.2(3) . . . . ?

C36 C31 O9 C8 -4.6(4) . . . . ?

C6 C8 O9 C31 -179.2(2) . . . . ?

_refine_diff_density_max   0.290

_refine_diff_density_min   -0.266

_refine_diff_density_rms   0.062

_shelx_res_file

;

shelx.res created by SHELXL-2014/7

TITL A2_twin1 in P-1

CELL 0.71073   8.6773   9.3488 10.6905   89.835   70.387   76.972

ZERR   2.00   0.0015   0.0016   0.0015   0.013   0.014   0.015

LATT   1

SFAC C   H   N   O   F   S   CL

UNIT 30 26 6 4 2 2 2

MERG   2

```
FMAP   2

ACTA

BOND   $H

PLAN  -2

CONF

L.S.  40

WGHT    0.070300

EXTI    0.017198

BASF   0.63052

FVAR       4.37651

MOLE    1

C2    1    0.025347    0.820073    0.108883    11.00000    0.03003    0.03781 =

         0.04081    0.00141   -0.01510   -0.00962

C6    1    0.304284    0.508504   -0.105820    11.00000    0.03106    0.04322 =

         0.03860   -0.00148   -0.01391   -0.01243

C8    1    0.490832    0.464949   -0.184544    11.00000    0.03180    0.04512 =

         0.05073   -0.00762   -0.00977   -0.01596

AFIX  23
```

H8A 2 0.541090 0.371511 -0.158305 11.00000 -1.50000

H8B 2 0.508530 0.453666 -0.278870 11.00000 -1.50000

AFIX 0

C21 1 -0.238119 0.967633 0.278390 11.00000 0.03330 0.03472 =

0.04377 0.00107 -0.00670 -0.01420

C22 1 -0.402138 1.032583 0.286012 11.00000 0.03419 0.04126 =

0.05015 0.00492 -0.00865 -0.01064

C23 1 -0.502541 1.147411 0.381399 11.00000 0.04279 0.04718 =

0.06910 0.00006 -0.00103 0.00173

AFIX 43

H23 2 -0.611689 1.190298 0.384792 11.00000 -1.50000

AFIX 0

C24 1 -0.439592 1.196895 0.470288 11.00000 0.06768 0.05248 =

0.06688 -0.01902 0.00088 -0.01216

AFIX 43

H24 2 -0.505279 1.274771 0.533126 11.00000 -1.50000

AFIX 0

C25 1 -0.278945 1.131127 0.466408 11.00000 0.06689 0.06579 =

0.06503 -0.02040 -0.01002 -0.03012

AFIX 43

H25 2 -0.237873 1.163298 0.528411 11.00000 -1.50000

AFIX 0

C26 1 -0.177748 1.017429 0.371026 11.00000 0.04444 0.05312 =

0.06009 -0.00883 -0.01638 -0.01988

AFIX 43

H26 2 -0.069228 0.974408 0.369055 11.00000 -1.50000

AFIX 0

C31 1 0.739985 0.555549 -0.225476 11.00000 0.02556 0.04028 =

0.04014 0.00409 -0.00979 -0.00971

C32 1 0.808429 0.669335 -0.203309 11.00000 0.03357 0.03792 =

0.05348 0.00028 -0.01418 -0.01043

AFIX 43

H32 2 0.739303 0.753074 -0.149241 11.00000 -1.50000

AFIX 0

C33 1 0.979774 0.658365 -0.261666 11.00000 0.03650 0.05127 =

0.05911 0.00874 -0.01902 -0.01838

AFIX 43

H33 2 1.026978 0.734283 -0.247967 11.00000 -1.50000

AFIX 0

C34 1 1.078145 0.532948 -0.340103 11.00000 0.02662 0.05805 =

0.05152 0.01324 -0.00904 -0.01434

C35 1 1.014505 0.418883 -0.364089 11.00000 0.03520 0.04704 =

0.05105 0.00285 -0.00682 -0.00243

AFIX 43

H35 2 1.084784 0.335585 -0.418235 11.00000 -1.50000

AFIX 0

C36 1 0.842279 0.430141 -0.305718 11.00000 0.03495 0.04262 =

0.04724 -0.00364 -0.01036 -0.01084

AFIX 43

H36 2 0.796030 0.353966 -0.320444 11.00000 -1.50000

AFIX 0

N1 3 -0.142073 0.848168 0.181762 11.00000 0.02765 0.03950 =

0.05421 -0.00881 -0.01283 -0.01203

H1 2 -0.199401 0.797820 0.163166 11.00000 -1.50000

N4 3 0.075458 0.694473 0.027610 11.00000 0.02355 0.04707 =

0.05577 -0.00993 -0.00669 -0.01459

H4 2 0.009903 0.632040 0.029781 11.00000 -1.50000

N5 3 0.243859 0.645555 -0.050251 11.00000 0.02429 0.04469 =

0.05719 -0.00851 -0.00646 -0.01465

H5 2 0.303357 0.717844 -0.050391 11.00000 -1.50000

O7 4 0.215712 0.419756 -0.097853 11.00000 0.03181 0.05143 =

0.06144 -0.00863 -0.01103 -0.01972

O9 4 0.568034 0.576198 -0.159790 11.00000 0.02447 0.04351 =

0.06430 -0.01170 -0.00586 -0.01287

F34 5 1.248404 0.520460 -0.397550 11.00000 0.02655 0.08893 =

0.09124 0.01133 -0.00683 -0.01601

S3 6 0.159092 0.921939 0.111511 11.00000 0.03472 0.04739 =

0.07955 -0.00820 -0.01348 -0.02120

CL22 7 -0.488189 0.965030 0.179525 11.00000 0.03836 0.07809 =

0.07192 -0.00152 -0.02523 -0.00490

HKLF 5

REM A2_twin1 in P-1

REM R1 = 0.0523 for   3395 Fo > 4sig(Fo) and 0.1047 for all   6495 data

REM   219 parameters refined using   0 restraints

END

WGHT   0.0704   0.0000

REM Highest difference peak 0.290, deepest hole -0.266, 1-sigma level 0.062

Q1   1 -0.4634 1.0398 0.2107 11.00000 0.05   0.25

Q2   1   0.0924 1.0394 0.1013 11.00000 0.05   0.25

;

CIF AB5

data_shelx

_audit_creation_method   'SHELXL-2014/7'

_shelx_SHELXL_version_number '2014/7'

_chemical_name_systematic ?

_chemical_name_common ?

_chemical_melting_point ?

_chemical_formula_moiety 'C15 H12 Cl2 F N3 O2 S'

_chemical_formula_sum 'C15 H12 Cl2 F N3 O2 S'

_chemical_formula_weight 388.24

loop_

_atom_type_symbol

_atom_type_description

_atom_type_scat_dispersion_real

_atom_type_scat_dispersion_imag

_atom_type_scat_source

'C' 'C' 0.0033 0.0016

'International Tables Vol C Tables 4.2.6.8 and 6.1.1.4'

'H' 'H' 0.0000 0.0000

'International Tables Vol C Tables 4.2.6.8 and 6.1.1.4'

'N' 'N' 0.0061 0.0033

'International Tables Vol C Tables 4.2.6.8 and 6.1.1.4'

'O' 'O' 0.0106 0.0060

'International Tables Vol C Tables 4.2.6.8 and 6.1.1.4'

'F' 'F' 0.0171 0.0103

'International Tables Vol C Tables 4.2.6.8 and 6.1.1.4'

'S' 'S' 0.1246 0.1234

'International Tables Vol C Tables 4.2.6.8 and 6.1.1.4'

'Cl' 'Cl' 0.1484 0.1585

'International Tables Vol C Tables 4.2.6.8 and 6.1.1.4'

_space_group_crystal_system triclinic

_space_group_IT_number 2

_space_group_name_H-M_alt 'P -1'

_space_group_name_Hall '-P 1'

_shelx_space_group_comment

;

The symmetry employed for this shelxl refinement is uniquely defined

by the following loop, which should always be used as a source of

symmetry information in preference to the above space-group names.

They are only intended as comments.

;

loop_

_space_group_symop_operation_xyz

'x, y, z'

'-x, -y, -z'

_cell_length_a 8.7626(8)

_cell_length_b 9.5122(6)

_cell_length_c 10.7018(9)

_cell_angle_alpha 86.446(6)

_cell_angle_beta 72.915(7)

_cell_angle_gamma 77.803(6)

_cell_volume 833.40(12)

_cell_formula_units_Z 2

_cell_measurement_temperature 293(2)

_cell_measurement_reflns_used 1797

_cell_measurement_theta_min 2.0080

_cell_measurement_theta_max 27.6380

_exptl_crystal_description prism

_exptl_crystal_colour colourless

_exptl_crystal_density_meas ?

_exptl_crystal_density_method ?

_exptl_crystal_density_diffrn 1.547

_exptl_crystal_F_000 396

_exptl_transmission_factor_min ?

_exptl_transmission_factor_max ?

_exptl_crystal_size_max 0.40

_exptl_crystal_size_mid 0.20

_exptl_crystal_size_min 0.10

_exptl_absorpt_coefficient_mu 0.538

_shelx_estimated_absorpt_T_min   ?

_shelx_estimated_absorpt_T_max   ?

_exptl_absorpt_correction_T_min   0.73016

_exptl_absorpt_correction_T_max   1.00000

_exptl_absorpt_correction_type   'multi-scan'

_exptl_absorpt_process_details

;

CrysAlisPro, Agilent Technologies,

Version 1.171.37.35h (release 09-02-2015 CrysAlis171 .NET)

(compiled Feb 9 2015,16:26:32)

Empirical absorption correction using spherical harmonics,

 implemented in SCALE3 ABSPACK scaling algorithm.

;

_exptl_absorpt_special_details   ?

_diffrn_ambient_temperature   293(2)

_diffrn_radiation_wavelength   0.71073

_diffrn_radiation_type   MoK\a

_diffrn_source   ?

_diffrn_measurement_device_type 'KM4 CCD four-circle diffractometer'

_diffrn_measurement_method '\w scans'

_diffrn_detector_area_resol_mean ?

_diffrn_reflns_number 5484

_diffrn_reflns_av_unetI/netI 0.0504

_diffrn_reflns_av_R_equivalents 0.0268

_diffrn_reflns_limit_h_min -4

_diffrn_reflns_limit_h_max 11

_diffrn_reflns_limit_k_min -12

_diffrn_reflns_limit_k_max 12

_diffrn_reflns_limit_l_min -13

_diffrn_reflns_limit_l_max 14

_diffrn_reflns_theta_min 2.190

_diffrn_reflns_theta_max 28.517

_diffrn_reflns_theta_full 25.242

_diffrn_measured_fraction_theta_max 0.864

_diffrn_measured_fraction_theta_full 0.999

_diffrn_reflns_Laue_measured_fraction_max 0.864

_diffrn_reflns_Laue_measured_fraction_full   0.999

_diffrn_reflns_point_group_measured_fraction_max   0.864

_diffrn_reflns_point_group_measured_fraction_full 0.999

_reflns_number_total   3654

_reflns_number_gt   2650

_reflns_threshold_expression   'I > 2\s(I)'

_reflns_Friedel_coverage   0.000

_reflns_Friedel_fraction_max   .

_reflns_Friedel_fraction_full   .

_reflns_special_details

;

Reflections were merged by SHELXL according to the crystal

class for the calculation of statistics and refinement.

_reflns_Friedel_fraction is defined as the number of unique

Friedel pairs measured divided by the number that would be

possible theoretically, ignoring centric projections and

systematic absences.

;

_computing_data_collection

;

CrysAlisPro, Agilent Technologies,

Version 1.171.37.35h (release 09-02-2015 CrysAlis171 .NET)

(compiled Feb 9 2015,16:26:32)

;

_computing_cell_refinement

;

CrysAlisPro, Agilent Technologies,

Version 1.171.37.35h (release 09-02-2015 CrysAlis171 .NET)

(compiled Feb 9 2015,16:26:32)

;

_computing_data_reduction

;

CrysAlisPro, Agilent Technologies,

Version 1.171.37.35h (release 09-02-2015 CrysAlis171 .NET)

(compiled Feb 9 2015,16:26:32)

;

_computing_structure_solution 'SHELXS-2013/1 (Sheldrick, 2013)'

_computing_structure_refinement 'SHELXL-2014/7 (Sheldrick, 2014)'

_computing_molecular_graphics 'ORTEP3 for Windows (Farrugia, 2012)'

_computing_publication_material 'SHELXL-2014/7 and WINGX (Farrugia, 2012)'

_refine_special_details ?

_refine_ls_structure_factor_coef Fsqd

_refine_ls_matrix_type full

_refine_ls_weighting_scheme calc

_refine_ls_weighting_details

'w=1/[\s^2^(Fo^2^)+(0.0593P)^2^+0.2635P] where P=(Fo^2^+2Fc^2^)/3'

_atom_sites_solution_primary difmap

_atom_sites_solution_secondary difmap

_atom_sites_solution_hydrogens mixed

_refine_ls_hydrogen_treatment mixed

_refine_ls_extinction_method 'SHELXL-2014/7 (Sheldrick 2014'

_refine_ls_extinction_coef 0.013(3)

_refine_ls_extinction_expression

'Fc^*^=kFc[1+0.001xFc^2^\l^3^/sin(2\q)]^-1/4^'

_refine_ls_number_reflns 3654

_refine_ls_number_parameters 227

_refine_ls_number_restraints 0

_refine_ls_R_factor_all 0.0759

_refine_ls_R_factor_gt 0.0499

_refine_ls_wR_factor_ref 0.1396

_refine_ls_wR_factor_gt 0.1167

_refine_ls_goodness_of_fit_ref 1.041

_refine_ls_restrained_S_all 1.041

_refine_ls_shift/su_max 0.000

_refine_ls_shift/su_mean 0.000

loop_

_atom_site_label

_atom_site_type_symbol

_atom_site_fract_x

_atom_site_fract_y

_atom_site_fract_z

_atom_site_U_iso_or_equiv

_atom_site_adp_type

_atom_site_occupancy

_atom_site_site_symmetry_order

_atom_site_calc_flag

_atom_site_refinement_flags_posn

_atom_site_refinement_flags_adp

_atom_site_refinement_flags_occupancy

_atom_site_disorder_assembly

_atom_site_disorder_group

C2 C 0.0259(3) 0.8054(3) 0.1121(3) 0.0406(6) Uani 1 1 d . . . . .

C6 C 0.2883(3) 0.5245(3) -0.0998(3) 0.0380(6) Uani 1 1 d . . . . .

C8 C 0.4655(3) 0.4919(3) -0.1740(3) 0.0439(7) Uani 1 1 d . . . . .

H8A H 0.5166 0.3982 -0.1486 0.066 Uiso 1 1 calc R U . . .

H8B H 0.4779 0.4899 -0.2670 0.066 Uiso 1 1 calc R U . . .

C21 C -0.2245(3) 0.9386(3) 0.2665(3) 0.0402(6) Uani 1 1 d . . . . .

C22 C -0.3833(3) 0.9974(3) 0.2636(3) 0.0401(6) Uani 1 1 d . . . . .

C23 C -0.4797(3) 1.1087(3) 0.3461(3) 0.0473(7) Uani 1 1 d . . . . .

H23 H -0.5857 1.1463 0.3436 0.071 Uiso 1 1 calc R U . . .

C24 C -0.4169(4) 1.1632(3) 0.4317(3) 0.0468(7) Uani 1 1 d . . . . .

C25 C -0.2632(4) 1.1051(3) 0.4402(3) 0.0543(8) Uani 1 1 d . . . . .

H25 H -0.2231 1.1407 0.5005 0.081 Uiso 1 1 calc R U . . .

C26 C -0.1681(3) 0.9927(3) 0.3579(3) 0.0522(8) Uani 1 1 d . . . . .

H26 H -0.0642 0.9527 0.3642 0.078 Uiso 1 1 calc R U . . .

C31 C 0.7061(3) 0.5875(3) -0.2085(3) 0.0372(6) Uani 1 1 d . . . . .

C32 C 0.7726(3) 0.6999(3) -0.1843(3) 0.0428(6) Uani 1 1 d . . . . .

H32 H 0.7065 0.7761 -0.1311 0.064 Uiso 1 1 calc R U . . .

C33 C 0.9367(3) 0.6984(3) -0.2395(3) 0.0486(7) Uani 1 1 d . . . . .

H33 H 0.9824 0.7733 -0.2246 0.073 Uiso 1 1 calc R U . . .

C34 C 1.0305(3) 0.5837(3) -0.3169(3) 0.0493(7) Uani 1 1 d . . . . .

C35 C 0.9680(3) 0.4719(3) -0.3412(3) 0.0489(7) Uani 1 1 d . . . . .

H35 H 1.0352 0.3952 -0.3931 0.073 Uiso 1 1 calc R U . . .

C36 C 0.8029(3) 0.4742(3) -0.2874(3) 0.0445(6) Uani 1 1 d . . . . .

H36 H 0.7579 0.3998 -0.3044 0.067 Uiso 1 1 calc R U . . .

N1 N -0.1333(3) 0.8239(2) 0.1823(3) 0.0441(6) Uani 1 1 d . . . . .

H1 H -0.185(4) 0.770(4) 0.163(3) 0.066 Uiso 1 1 d . U . . .

N4 N 0.0726(3) 0.6897(3) 0.0333(2) 0.0446(6) Uani 1 1 d . . . . .

H4 H 0.011(4) 0.632(4) 0.036(3) 0.067 Uiso 1 1 d . U . . .

N5 N 0.2329(3) 0.6507(3) -0.0409(2) 0.0445(6) Uani 1 1 d . . . . .

H5 H 0.296(4) 0.712(3) -0.041(3) 0.067 Uiso 1 1 d . U . . .

O7 O 0.2011(2) 0.4368(2) -0.0955(2) 0.0494(5) Uani 1 1 d . . . . .

O9 O 0.5421(2) 0.5992(2) -0.14666(19) 0.0472(5) Uani 1 1 d . . . . .

F34 F 1.1925(2) 0.5810(2) -0.3698(2) 0.0747(6) Uani 1 1 d . . . . .

S3 S 0.15623(9) 0.90936(9) 0.11458(10) 0.0618(3) Uani 1 1 d . . . . .

Cl22 Cl -0.46645(9) 0.92838(9) 0.15866(8) 0.0589(3) Uani 1 1 d . . . . .

Cl24 Cl -0.53803(11) 1.30669(9) 0.53041(9) 0.0706(3) Uani 1 1 d . . . . .

loop_

_atom_site_aniso_label

_atom_site_aniso_U_11

_atom_site_aniso_U_22

_atom_site_aniso_U_33

_atom_site_aniso_U_23

_atom_site_aniso_U_13

_atom_site_aniso_U_12

C2 0.0301(13) 0.0378(14) 0.0559(17) -0.0064(12) -0.0124(12) -0.0091(11)

C6 0.0307(12) 0.0415(14) 0.0448(15) -0.0061(12) -0.0105(11) -0.0125(11)

C8 0.0322(13) 0.0465(15) 0.0548(17) -0.0128(13) -0.0080(12) -0.0148(12)

C21 0.0318(13) 0.0373(14) 0.0527(16) -0.0062(12) -0.0090(11) -0.0121(11)

C22 0.0340(13) 0.0382(14) 0.0488(16) -0.0024(12) -0.0115(12) -0.0088(11)

C23 0.0367(14) 0.0435(15) 0.0550(17) 0.0015(13) -0.0049(13) -0.0059(12)

C24 0.0493(16) 0.0355(14) 0.0508(16) -0.0081(12) -0.0018(13) -0.0141(13)

C25 0.0531(18) 0.0547(18) 0.0593(19) -0.0144(15) -0.0124(15) -0.0209(15)

C26 0.0387(15) 0.0531(17) 0.069(2) -0.0130(15) -0.0162(14) -0.0129(14)

C31 0.0260(12) 0.0410(14) 0.0456(15) -0.0026(11) -0.0092(11) -0.0097(11)

C32 0.0337(13) 0.0409(14) 0.0546(17) -0.0055(12) -0.0096(12) -0.0118(12)

C33 0.0374(14) 0.0498(17) 0.0624(19) -0.0008(14) -0.0139(13) -0.0176(13)

C34 0.0271(13) 0.0581(18) 0.0589(18) 0.0077(15) -0.0050(12) -0.0132(13)

C35 0.0372(15) 0.0478(16) 0.0544(17) -0.0039(13) -0.0022(13) -0.0074(13)

C36 0.0364(14) 0.0429(15) 0.0529(16) -0.0072(12) -0.0061(12) -0.0125(12)

N1 0.0269(11) 0.0397(12) 0.0671(16) -0.0160(11) -0.0092(10) -0.0111(9)

N4 0.0253(10) 0.0452(13) 0.0639(15) -0.0160(11) -0.0052(10) -0.0145(10)

N5 0.0264(11) 0.0448(13) 0.0617(15) -0.0130(11) -0.0040(10) -0.0147(10)

O7 0.0341(10) 0.0506(11) 0.0665(13) -0.0124(10) -0.0086(9) -0.0194(9)

O9 0.0276(9) 0.0465(11) 0.0665(13) -0.0190(9) -0.0027(8) -0.0152(8)

F34 0.0294(9) 0.0791(13) 0.1035(15) -0.0023(11) 0.0046(9) -0.0178(9)

S3 0.0371(4) 0.0520(5) 0.0976(7) -0.0244(4) -0.0066(4) -0.0227(3)

Cl22 0.0432(4) 0.0664(5) 0.0726(5) -0.0178(4) -0.0255(4) -0.0049(4)

Cl24 0.0735(6) 0.0509(5) 0.0722(6) -0.0223(4) 0.0062(4) -0.0119(4)

_geom_special_details

;

All esds (except the esd in the dihedral angle between two l.s. planes)

are estimated using the full covariance matrix. The cell esds are taken

into account individually in the estimation of esds in distances, angles

and torsion angles; correlations between esds in cell parameters are only

used when they are defined by crystal symmetry. An approximate (isotropic)

treatment of cell esds is used for estimating esds involving l.s. planes.

;

loop_

_geom_bond_atom_site_label_1

_geom_bond_atom_site_label_2

_geom_bond_distance

_geom_bond_site_symmetry_2

_geom_bond_publ_flag

C2 N4 1.345(3) . ?

C2 N1 1.356(3) . ?

C2 S3 1.668(3) . ?

C6 O7 1.236(3) . ?

C6 N5 1.320(3) . ?

C6 C8 1.496(3) . ?

C8 O9 1.421(3) . ?

C8 H8A 0.9700 . ?

C8 H8B 0.9700 . ?

C21 C26 1.386(4) . ?

C21 C22 1.395(3) . ?

C21 N1 1.404(3) . ?

C22 C23 1.384(4) . ?

C22 Cl22 1.731(3) . ?

C23 C24 1.373(4) . ?

C23 H23 0.9300 . ?

C24 C25 1.371(4) . ?

C24 Cl24 1.736(3) . ?

C25 C26 1.387(4) . ?

C25 H25 0.9300 . ?

C26 H26 0.9300 . ?

C31 O9 1.378(3) . ?

C31 C36 1.379(4) . ?

C31 C32 1.391(3) . ?

C32 C33 1.382(4) . ?

C32 H32 0.9300 . ?

C33 C34 1.373(4) . ?

C33 H33 0.9300 . ?

C34 F34 1.360(3) . ?

C34 C35 1.364(4) . ?

C35 C36 1.387(4) . ?

C35 H35 0.9300 . ?

C36 H36 0.9300 . ?

N1 H1 0.83(3) . ?

N4 N5 1.380(3) . ?

N4 H4 0.84(3) . ?

N5 H5 0.88(3) . ?

loop_

_geom_angle_atom_site_label_1

_geom_angle_atom_site_label_2

_geom_angle_atom_site_label_3

_geom_angle

_geom_angle_site_symmetry_1

_geom_angle_site_symmetry_3

_geom_angle_publ_flag

N4 C2 N1 112.0(2) . . ?

N4 C2 S3 121.0(2) . . ?

N1 C2 S3 127.0(2) . . ?

O7 C6 N5 123.0(2) . . ?

O7 C6 C8 120.9(2) . . ?

N5 C6 C8 116.2(2) . . ?

O9 C8 C6 109.4(2) . . ?

O9 C8 H8A 109.8 . . ?

C6 C8 H8A 109.8 . . ?

O9 C8 H8B 109.8 . . ?

C6 C8 H8B 109.8 . . ?

H8A C8 H8B 108.2 . . ?

C26 C21 C22 117.6(2) . . ?

C26 C21 N1 123.4(2) . . ?

C22 C21 N1 118.9(2) . . ?

C23 C22 C21 121.3(3) . . ?

C23 C22 Cl22 118.4(2) . . ?

C21 C22 Cl22 120.3(2) . . ?

C24 C23 C22 119.2(3) . . ?

C24 C23 H23 120.4 . . ?

C22 C23 H23 120.4 . . ?

C25 C24 C23 121.0(3) . . ?

C25 C24 Cl24 120.7(2) . . ?

C23 C24 Cl24 118.3(2) . . ?

C24 C25 C26 119.3(3) . . ?

C24 C25 H25 120.4 . . ?

C26 C25 H25 120.4 . . ?

C21 C26 C25 121.4(3) . . ?

C21 C26 H26 119.3 . . ?

C25 C26 H26 119.3 . . ?

O9 C31 C36 124.6(2) . . ?

O9 C31 C32 115.0(2) . . ?

C36 C31 C32 120.4(2) . . ?

C33 C32 C31 120.0(2) . . ?

C33 C32 H32 120.0 . . ?

C31 C32 H32 120.0 . . ?

C34 C33 C32 118.3(3) . . ?

C34 C33 H33 120.8 . . ?

C32 C33 H33 120.8 . . ?

F34 C34 C35 119.0(3) . . ?

F34 C34 C33 118.4(3) . . ?

C35 C34 C33 122.6(3) . . ?

C34 C35 C36 119.2(3) . . ?

C34 C35 H35 120.4 . . ?

C36 C35 H35 120.4 . . ?

C31 C36 C35 119.4(3) . . ?

C31 C36 H36 120.3 . . ?

C35 C36 H36 120.3 . . ?

C2 N1 C21 127.1(2) . . ?

C2 N1 H1 116(2) . . ?

C21 N1 H1 116(2) . . ?

C2 N4 N5 119.8(2) . . ?

C2 N4 H4 122(2) . . ?

N5 N4 H4 118(2) . . ?

C6 N5 N4 120.9(2) . . ?

C6 N5 H5 123(2) . . ?

N4 N5 H5 116(2) . . ?

C31 O9 C8 117.41(19) . . ?

loop_

_geom_torsion_atom_site_label_1

_geom_torsion_atom_site_label_2

_geom_torsion_atom_site_label_3

_geom_torsion_atom_site_label_4

_geom_torsion

_geom_torsion_site_symmetry_1

_geom_torsion_site_symmetry_2

_geom_torsion_site_symmetry_3

_geom_torsion_site_symmetry_4

_geom_torsion_publ_flag

O7 C6 C8 O9 -171.0(2) . . . . ?

N5 C6 C8 O9 9.1(4) . . . . ?

C26 C21 C22 C23 1.9(4) . . . . ?

N1 C21 C22 C23 178.8(2) . . . . ?

C26 C21 C22 Cl22 -176.2(2) . . . . ?

N1 C21 C22 Cl22 0.6(4) . . . . ?

C21 C22 C23 C24 0.6(4) . . . . ?

Cl22 C22 C23 C24 178.8(2) . . . . ?

C22 C23 C24 C25 -2.7(4) . . . . ?

C22 C23 C24 Cl24 177.9(2) . . . . ?

C23 C24 C25 C26 2.1(4) . . . . ?

Cl24 C24 C25 C26 -178.5(2) . . . . ?

C22 C21 C26 C25 -2.5(4) . . . . ?

N1 C21 C26 C25 -179.2(3) . . . . ?

C24 C25 C26 C21 0.6(5) . . . . ?

O9 C31 C32 C33 178.9(2) . . . . ?

C36 C31 C32 C33 -0.1(4) . . . . ?

C31 C32 C33 C34 -0.4(4) . . . . ?

C32 C33 C34 F34 -179.1(3) . . . . ?

C32 C33 C34 C35 0.0(5) . . . . ?

F34 C34 C35 C36 180.0(3) . . . . ?

C33 C34 C35 C36 0.8(5) . . . . ?

O9 C31 C36 C35 -178.0(3) . . . . ?

C32 C31 C36 C35 1.0(4) . . . . ?

C34 C35 C36 C31 -1.3(4) . . . . ?

N4 C2 N1 C21 -175.1(3) . . . . ?

S3 C2 N1 C21 3.7(4) . . . . ?

C26 C21 N1 C2 -45.4(4) . . . . ?

C22 C21 N1 C2 137.9(3) . . . . ?

N1 C2 N4 N5 -177.0(2) . . . . ?

S3 C2 N4 N5 4.1(4) . . . . ?

O7 C6 N5 N4 2.1(4) . . . . ?

C8 C6 N5 N4 -178.1(2) . . . . ?

C2 N4 N5 C6 167.4(3) . . . . ?

C36 C31 O9 C8 -4.7(4) . . . . ?

C32 C31 O9 C8 176.3(2) . . . . ?

C6 C8 O9 C31 -179.1(2) . . . . ?

_refine_diff_density_max   0.316

_refine_diff_density_min   -0.347

_refine_diff_density_rms   0.069

_shelx_res_file

;

shelx.res created by SHELXL-2014/7

TITL A5 in space group P -1

CELL 0.71073   8.7626   9.5122 10.7018   86.446   72.915   77.803

ZERR   2.00   0.0008   0.0006   0.0009   0.006   0.007   0.006

LATT   1

SFAC C   H   N   O   F   S   CL

UNIT 30 24 6 4 2 2 4

MERG   2

```
FMAP   2

OMIT   0  0  1

ACTA

BOND   $H

CONF

L.S.  40

PLAN  -2

WGHT    0.059300    0.263500

EXTI    0.012854

FVAR       3.62535

MOLE    1

C2    1    0.025930    0.805353    0.112124    11.00000    0.03011    0.03779 =

         0.05588   -0.00635   -0.01240   -0.00913

C6    1    0.288344    0.524495   -0.099785    11.00000    0.03067    0.04148 =

         0.04475   -0.00606   -0.01048   -0.01250

C8    1    0.465511    0.491907   -0.173950    11.00000    0.03221    0.04654 =

         0.05485   -0.01280   -0.00805   -0.01481

AFIX  23
```

H8A 2 0.516558 0.398226 -0.148569 11.00000 -1.50000

H8B 2 0.477884 0.489909 -0.266958 11.00000 -1.50000

AFIX 0

C21 1 -0.224527 0.938601 0.266506 11.00000 0.03180 0.03728 =

0.05272 -0.00617 -0.00901 -0.01209

C22 1 -0.383325 0.997384 0.263564 11.00000 0.03402 0.03821 =

0.04880 -0.00235 -0.01151 -0.00881

C23 1 -0.479683 1.108738 0.346116 11.00000 0.03672 0.04353 =

0.05504 0.00155 -0.00490 -0.00593

AFIX 43

H23 2 -0.585734 1.146274 0.343633 11.00000 -1.50000

AFIX 0

C24 1 -0.416908 1.163167 0.431736 11.00000 0.04929 0.03554 =

0.05079 -0.00807 -0.00183 -0.01408

C25 1 -0.263190 1.105078 0.440218 11.00000 0.05307 0.05467 =

0.05928 -0.01443 -0.01237 -0.02091

AFIX 43

H25 2 -0.223123 1.140670 0.500526 11.00000 -1.50000

AFIX 0

C26 1 -0.168087 0.992673 0.357901 11.00000 0.03871 0.05307 =

0.06860 -0.01298 -0.01617 -0.01293

AFIX 43

H26 2 -0.064154 0.952705 0.364171 11.00000 -1.50000

AFIX 0

C31 1 0.706096 0.587537 -0.208451 11.00000 0.02599 0.04098 =

0.04559 -0.00258 -0.00921 -0.00974

C32 1 0.772561 0.699946 -0.184345 11.00000 0.03373 0.04085 =

0.05461 -0.00547 -0.00964 -0.01184

AFIX 43

H32 2 0.706546 0.776141 -0.131110 11.00000 -1.50000

AFIX 0

C33 1 0.936689 0.698444 -0.239483 11.00000 0.03745 0.04983 =

0.06240 -0.00080 -0.01393 -0.01759

AFIX 43

H33 2 0.982439 0.773261 -0.224566 11.00000 -1.50000

AFIX 0

C34 1 1.030502 0.583665 -0.316857 11.00000 0.02709 0.05810 =

0.05885 0.00769 -0.00504 -0.01321

C35 1 0.967969 0.471942 -0.341158 11.00000 0.03722 0.04777 =

0.05439 -0.00387 -0.00217 -0.00743

AFIX 43

H35 2 1.035203 0.395245 -0.393136 11.00000 -1.50000

AFIX 0

C36 1 0.802904 0.474165 -0.287388 11.00000 0.03643 0.04290 =

0.05285 -0.00718 -0.00613 -0.01252

AFIX 43

H36 2 0.757884 0.399832 -0.304421 11.00000 -1.50000

AFIX 0

N1 3 -0.133295 0.823933 0.182324 11.00000 0.02692 0.03972 =

0.06708 -0.01600 -0.00923 -0.01107

H1 2 -0.185429 0.769834 0.163314 11.00000 -1.50000

N4 3 0.072557 0.689722 0.033339 11.00000 0.02527 0.04522 =

0.06390 -0.01604 -0.00521 -0.01449

H4 2 0.011068 0.632020 0.036475 11.00000 -1.50000

N5 3 0.232939 0.650675 -0.040852 11.00000 0.02644 0.04478 =

0.06168 -0.01297 -0.00399 -0.01466

H5 2 0.295612 0.711775 -0.040848 11.00000 -1.50000

O7 4 0.201101 0.436766 -0.095518 11.00000 0.03408 0.05055 =

0.06646 -0.01235 -0.00856 -0.01944

O9 4 0.542105 0.599204 -0.146662 11.00000 0.02763 0.04654 =

0.06650 -0.01902 -0.00269 -0.01522

F34 5 1.192478 0.580973 -0.369781 11.00000 0.02936 0.07912 =

0.10352 -0.00229 0.00455 -0.01777

S3 6 0.156234 0.909364 0.114582 11.00000 0.03705 0.05201 =

0.09763 -0.02442 -0.00661 -0.02270

CL22 7 -0.466447 0.928384 0.158664 11.00000 0.04317 0.06636 =

0.07264 -0.01780 -0.02554 -0.00490

CL24 7 -0.538027 1.306689 0.530412 11.00000 0.07354 0.05094 =

0.07224 -0.02231 0.00615 -0.01187

HKLF 4

REM A5 in space group P -1

REM R1 = 0.0499 for 2650 Fo > 4sig(Fo) and 0.0759 for all 3654 data

REM 227 parameters refined using 0 restraints

END

WGHT 0.0426 0.3870

REM Highest difference peak 0.316, deepest hole -0.347, 1-sigma level 0.069

Q1 1 -0.4909 0.9833 0.0462 11.00000 0.05 0.32

Q2 1 -0.4665 1.2465 0.5867 11.00000 0.05 0.25

;

CHECKCIF AB2

checkCIF/PLATON (basic structural check)

Structure factors have been supplied for datablock(s) shelx

THIS REPORT IS FOR GUIDANCE ONLY. IF USED AS PART OF A REVIEW PROCEDURE FOR PUBLICATION, IT SHOULD NOT REPLACE THE EXPERTISE OF AN EXPERIENCED CRYSTALLOGRAPHIC REFEREE.

No syntax errors found. CIF dictionary
Please wait while processing .... Interpreting this report

Structure factor report

Datablock: shelx

Bond precision:	C-C = 0.0048 A		Wavelength=0.71073
Cell:	a=8.6773(15)	b=9.3488(16)	c=10.6905(15)
	alpha=89.835(13)	beta=70.387(14)	gamma=76.972(15)
Temperature:	293 K		

	Calculated	Reported
Volume	793.4(2)	793.4(2)
Space group	P -1	P -1
Hall group	-P 1	-P 1
Moiety formula	C15 H13 Cl F N3 O2 S	C15 H13 Cl F N3 O2 S
Sum formula	C15 H13 Cl F N3 O2 S	C15 H13 Cl F N3 O2 S

Mr	353.79	353.79
Dx,g cm-3	1.481	1.481
Z	2	2
Mu (mm-1)	0.395	0.395
F000	364.0	364.0
F000'	364.70	
h,k,lmax	11,12,14	11,11,13
Nref	4125	6495
Tmin,Tmax	0.910,0.961	0.609,1.000
Tmin'	0.888	

Correction method= # Reported T Limits: Tmin=0.609 Tmax=1.000
AbsCorr = MULTI-SCAN

Data completeness= 1.575 Theta(max)= 28.744

R(reflections)= 0.0523(3395) wR2(reflections)= 0.1320(6495)

S = 0.861 Npar= 219

The following ALERTS were generated. Each ALERT has the format

test-name_ALERT_alert-type_alert-level.

Click on the hyperlinks for more details of the test.

Alert level C

PLAT340_ALERT_3_C Low Bond Precision on C-C Bonds .... 0.00485 Ang.

PLAT906_ALERT_3_C Large K Value in the Analysis of Variance ...... 5.368 Check

PLAT918_ALERT_3_C Reflection(s) with I(obs) much Smaller I(calc) . 1 Check

---

Alert level G

PLAT199_ALERT_1_G Reported _cell_measurement_temperature ..... (K) 293 Check

PLAT200_ALERT_1_G Reported _diffrn_ambient_temperature ..... (K) 293 Check

PLAT870_ALERT_4_G ALERTS Related to Twinning Effects Suppressed .. ! Info

PLAT912_ALERT_4_G Missing # of FCF Reflections Above STh/L= 0.600 565 Note

PLAT941_ALERT_3_G Average HKL Measurement Multiplicity .... 1.9 Low

PLAT969_ALERT_5_G The 'Henn et al.' R-Factor-gap value .... 4.151 Note

Predicted wR2: Based on SigI**2 3.18 or SHELX Weight 15.33

---

0 **ALERT level A** = Most likely a serious problem - resolve or explain

0 **ALERT level B** = A potentially serious problem, consider carefully

3 **ALERT level C** = Check. Ensure it is not caused by an omission or oversight

6 **ALERT level G** = General information/check it is not something unexpected

2 ALERT type 1 CIF construction/syntax error, inconsistent or missing data

0 ALERT type 2 Indicator that the structure model may be wrong or deficient

4 ALERT type 3 Indicator that the structure quality may be low

2 ALERT type 4 Improvement, methodology, query or suggestion

1 ALERT type 5 Informative message, check

It is advisable to attempt to resolve as many as possible of the alerts in all categories. Often the minor alerts point to easily fixed oversights, errors and omissions in your CIF or refinement strategy, so attention to these fine details can be worthwhile. In order to resolve some of the more serious problems it may be necessary to carry out additional measurements or structure refinements. However, the purpose of your study may justify the reported deviations and the more serious of these should normally be commented upon in the discussion or experimental section of a paper or in the "special_details" fields of the CIF. checkCIF was carefully designed to identify outliers and unusual parameters, but every test has its limitations and alerts that are not important in a particular case may appear. Conversely, the absence of alerts does not guarantee there are no aspects of the results needing attention. It is up to the individual to critically assess their own results and, if necessary, seek expert advice.

Publication of your CIF in IUCr journals

A basic structural check has been run on your CIF. These basic checks will be run on all CIFs submitted for publication in IUCr journals (*Acta Crystallographica*, *Journal of Applied Crystallography*, *Journal of Synchrotron Radiation*); however, if you intend to submit to *Acta Crystallographica Section C* or *E* or *IUCrData*, you should make sure that full publication checks are run on the final version of your CIF prior to submission.

Publication of your CIF in other journals

Please refer to the *Notes for Authors* of the relevant journal for any special instructions relating to CIF submission.

PLATON version of 02/02/2025; check.def file version of 02/02/2025

Datablock shelx - ellipsoid plot

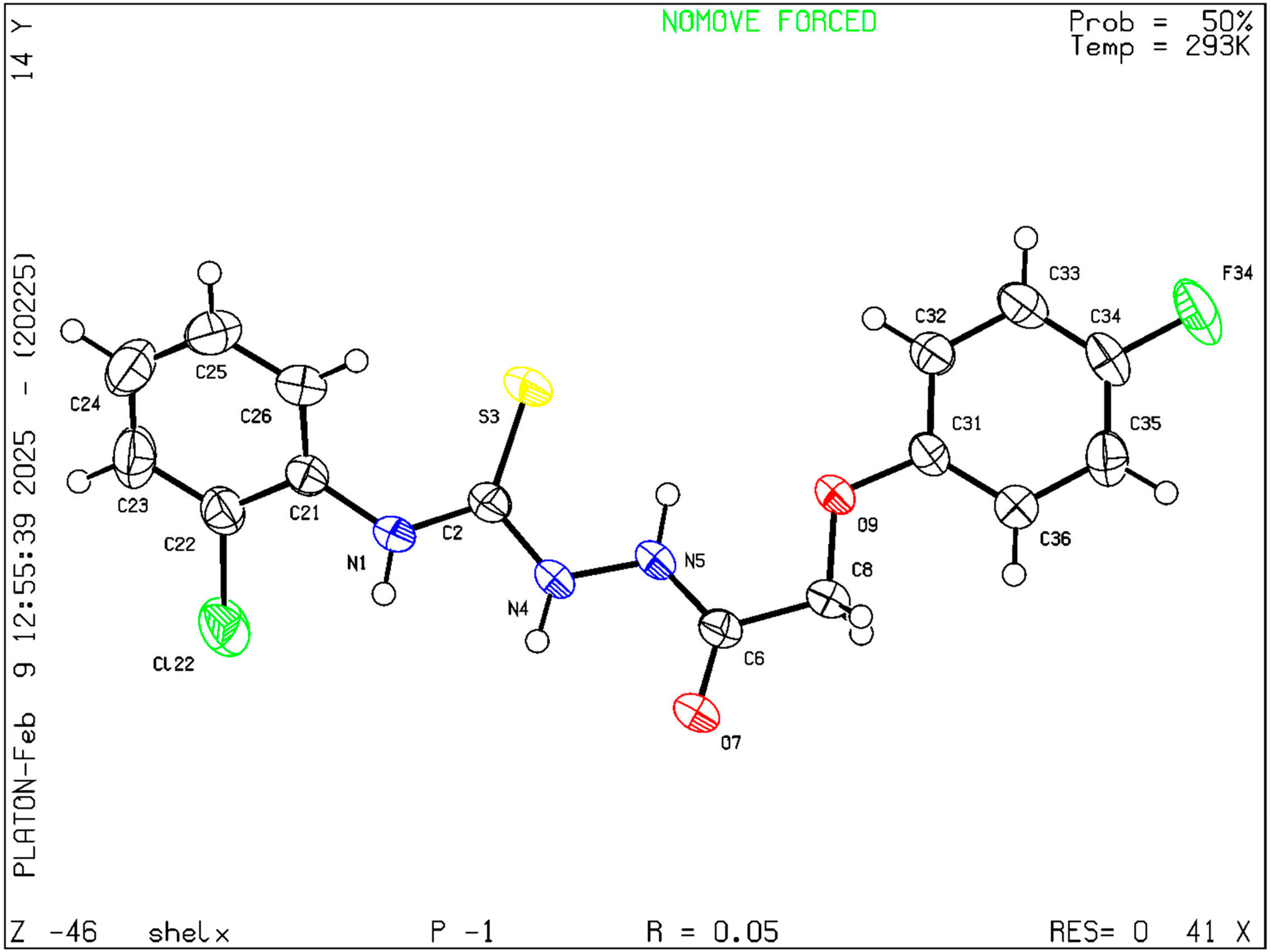
NOMOVE FORCED
Prob = 50%
Temp = 293K
14 Y
PLATON-Feb 9 12:55:39 2025 - (20225)
C25
C24
C26
S3
C33
F34
C32
C34
C31
C35
C23
C21
C2
O9
C36
C22
N1
N5
C8
N4
CL22
C6
O7
Z -46 shelx
P -1
R = 0.05
RES= 0 41 X

CHECKCIF AB5

checkCIF/PLATON (basic structural check)

Structure factors have been supplied for datablock(s) shelx

THIS REPORT IS FOR GUIDANCE ONLY. IF USED AS PART OF A REVIEW PROCEDURE FOR PUBLICATION, IT SHOULD NOT REPLACE THE EXPERTISE OF AN EXPERIENCED CRYSTALLOGRAPHIC REFEREE.

No syntax errors found. CIF dictionary
Please wait while processing .... Interpreting this report

Structure factor report

Datablock: shelx

Bond precision:	C-C = 0.0041 A		Wavelength=0.71073
Cell:	a=8.7626(8)	b=9.5122(6)	c=10.7018(9)
	alpha=86.446(6)	beta=72.915(7)	gamma=77.803(6)
Temperature:	293 K		

	Calculated	Reported
Volume	833.40(12)	833.40(12)

Space group	P -1	P -1
Hall group	-P 1	-P 1
Moiety formula	C15 H12 Cl2 F N3 O2 S	C15 H12 Cl2 F N3 O2 S
Sum formula	C15 H12 Cl2 F N3 O2 S	C15 H12 Cl2 F N3 O2 S
Mr	388.24	388.24
Dx,g cm-3	1.547	1.547
Z	2	2
Mu (mm-1)	0.538	0.538
F000	396.0	396.0
F000'	397.00	
h,k,lmax	11,12,14	11,12,14
Nref	4230	3654
Tmin,Tmax	0.879,0.948	0.730,1.000
Tmin'	0.806	

Correction method= # Reported T Limits: Tmin=0.730 Tmax=1.000
AbsCorr = MULTI-SCAN

Data completeness= 0.864 Theta(max)= 28.517

R(reflections)= 0.0499(2650)

wR2(reflections)= 0.1396(3654)

S = 1.041 Npar= 227

The following ALERTS were generated. Each ALERT has the format

test-name_ALERT_alert-type_alert-level.

Click on the hyperlinks for more details of the test.

Alert level C

PLAT250_ALERT_2_C Large U3/U1 Ratio for <U(i,j)> Tensor(Resd 1) 2.2 Note

PLAT340_ALERT_3_C Low Bond Precision on C-C Bonds .... 0.00408 Ang.

PLAT906_ALERT_3_C Large K Value in the Analysis of Variance ...... 3.547 Check

PLAT911_ALERT_3_C Missing FCF Refl Between Thmin & STh/L= 0.600 2 Report

6 4 7, 4 -1 12,

Alert level G

PLAT199_ALERT_1_G Reported _cell_measurement_temperature ..... (K) 293 Check

PLAT200_ALERT_1_G Reported _diffrn_ambient_temperature ..... (K) 293 Check

PLAT910_ALERT_3_G Missing # of FCF Reflection(s) Below Theta(Min). 1 Note

0 0 1,

PLAT912_ALERT_4_G Missing # of FCF Reflections Above STh/L= 0.600 573 Note

PLAT933_ALERT_2_G Number of HKL-OMIT Records in Embedded .res File 1 Note

0 0 1,

PLAT941_ALERT_3_G Average HKL Measurement Multiplicity .... 1.5 Low

PLAT965_ALERT_2_G The SHELXL WEIGHT Optimisation has not Converged Please Check

PLAT969_ALERT_5_G The 'Henn et al.' R-Factor-gap value .... 2.415 Note

Predicted wR2: Based on SigI**2 5.78 or SHELX Weight 13.41

PLAT978_ALERT_2_G Number C-C Bonds with Positive Residual Density. 4 Info

---

0 **ALERT level A** = Most likely a serious problem - resolve or explain

0 **ALERT level B** = A potentially serious problem, consider carefully

4 **ALERT level C** = Check. Ensure it is not caused by an omission or oversight

9 **ALERT level G** = General information/check it is not something unexpected

2 ALERT type 1 CIF construction/syntax error, inconsistent or missing data

4 ALERT type 2 Indicator that the structure model may be wrong or deficient

5 ALERT type 3 Indicator that the structure quality may be low

1 ALERT type 4 Improvement, methodology, query or suggestion

1 ALERT type 5 Informative message, check

It is advisable to attempt to resolve as many as possible of the alerts in all categories. Often the minor alerts point to easily fixed oversights, errors and omissions in your CIF or refinement strategy, so attention to these fine details can be worthwhile. In order to resolve some of the more serious problems it may be necessary to carry out additional measurements or structure refinements. However, the purpose of your study may justify the reported deviations and the more serious of these should normally be commented upon in the discussion or experimental section of a paper or in the "special_details" fields of the CIF. checkCIF was carefully designed to identify outliers and unusual parameters, but every test has its limitations and alerts that are not important in a particular case may appear. Conversely, the absence of alerts does not guarantee there are no aspects of the results needing attention. It is up to the individual to critically assess their own results and, if necessary, seek expert advice.

Publication of your CIF in IUCr journals

A basic structural check has been run on your CIF. These basic checks will be run on all CIFs submitted for publication in IUCr journals (*Acta Crystallographica*, *Journal of Applied Crystallography*, *Journal of Synchrotron Radiation*); however, if you intend to submit to *Acta Crystallographica Section C* or *E* or *IUCrData*, you should make sure that full publication checks are run on the final version of your CIF prior to submission.

Publication of your CIF in other journals

Please refer to the *Notes for Authors* of the relevant journal for any special instructions relating to CIF submission.

PLATON version of 02/02/2025; check.def file version of 02/02/2025

Datablock shelx - ellipsoid plot

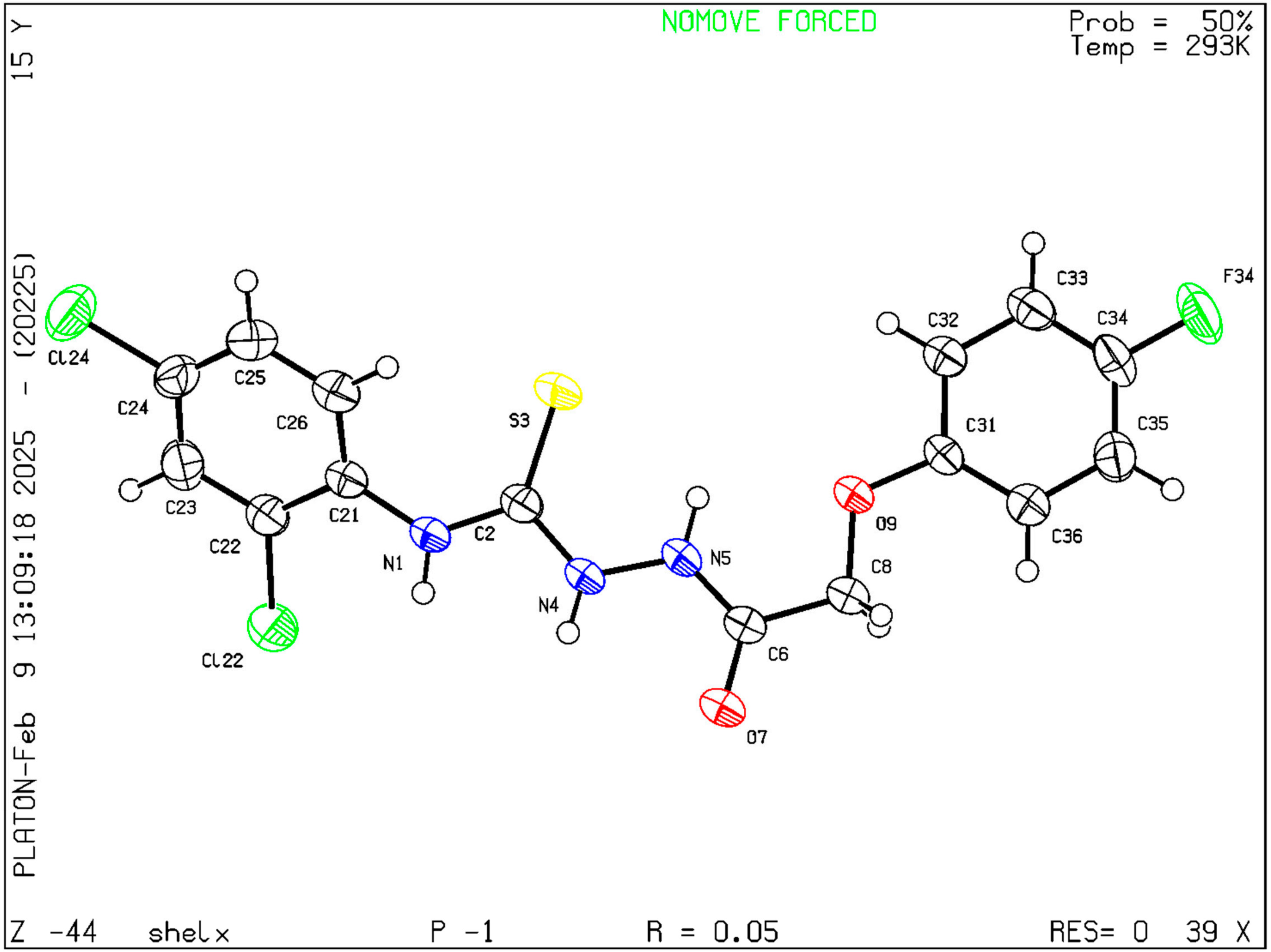
NOMOVE FORCED
Prob = 50%
Temp = 293K
15 Y
PLATON-Feb 9 13:09:18 2025 - (20225)
Cl24
C24
C25
C26
C23
C22
C21
Cl22
N1
C2
S3
N4
N5
C6
O7
C8
O9
C31
C32
C33
C34
F34
C35
C36
Z -44 shelx
P -1
R = 0.05
RES= 0 39 X
